# A structural-chemical explanation of fungal laccase activity

**DOI:** 10.1038/s41598-018-35633-8

**Published:** 2018-11-23

**Authors:** Rukmankesh Mehra, Jan Muschiol, Anne S. Meyer, Kasper P. Kepp

**Affiliations:** 1 Technical University of Denmark, DTU Chemistry, Building 206, 2800 Kgs Lyngby, Denmark; 2 Technical University of Denmark, DTU Bioengineering, Building 221, 2800 Kgs Lyngby, Denmark

## Abstract

Fungal laccases (EC 1.10.3.2) are multi-copper oxidases that oxidize a wide variety of substrates. Despite extensive studies, the molecular basis for their diverse activity is unclear. Notably, there is no current way to rationally predict the activity of a laccase toward a given substrate. Such knowledge would greatly facilitate the rational design of new laccases for technological purposes. We report a study of three datasets of experimental K_m_ values and activities for *Trametes versicolor* and *Cerrena unicolor* laccase, using a range of protein modeling techniques. We identify diverse binding modes of the various substrates and confirm an important role of Asp-206 and His-458 (*T*. *versicolor* laccase numbering) in guiding substrate recognition. Importantly, we demonstrate that experimental K_m_ values correlate with binding affinities computed by MMGBSA. This confirms the common assumption that the protein-substrate affinity is a major contributor to observed K_m_. From quantitative structure-activity relations (QSAR) we identify physicochemical properties that correlate with observed K_m_ and activities. In particular, the ionization potential, shape, and binding affinity of the substrate largely determine the enzyme’s K_m_ for the particular substrate. Our results suggest that K_m_ is not just a binding constant but also contains features of the enzymatic activity. In addition, we identify QSAR models with only a few descriptors showing that phenolic substrates employ optimal hydrophobic packing to reach the T1 site, but then require additional electronic properties to engage in the subsequent electron transfer. Our results advance our ability to model laccase activity and lend promise to future rational optimization of laccases toward phenolic substrates.

## Introduction

Laccases (EC 1.10.3.2) are multi-copper oxidoreductases that catalyze the one-electron (e^−^) oxidation of diverse substrates and sequentially transfer four electrons to the catalytic copper (Cu) atoms, which are used to reduce O_2_ to two water molecules^1–5^. Laccases are found in fungi, plants, bacteria and insects^6^ and catalyze the oxidation of a wide variety of organic and inorganic substrates including phenols, ketones, phosphates, ascorbate, amines and lignin^7–11^. Laccases are attractive industrial biocatalysts^12–15^ and thus the relationships between their specific structures and associated functions are of major interest, in particular to guide the design of new laccases for tailored purposes^7^.

**Figure 1:**
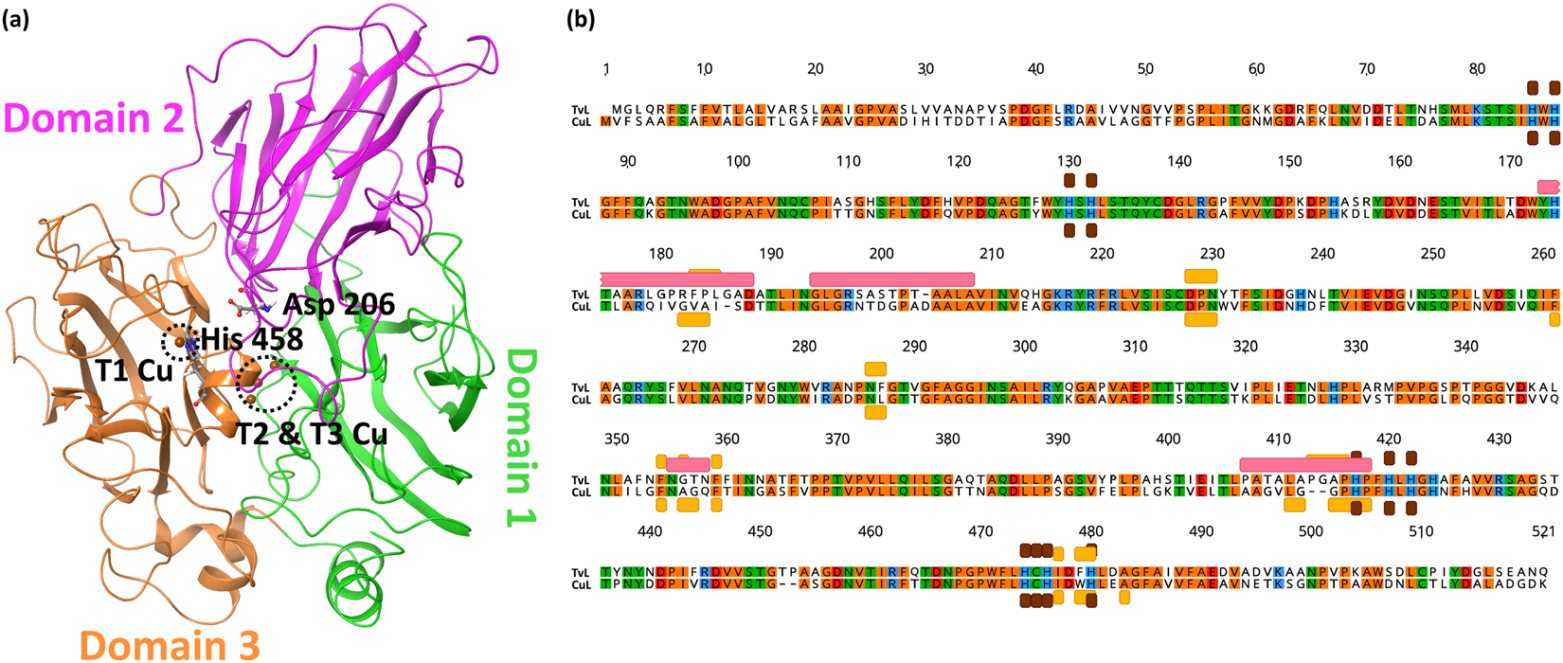
(**a**) TvL (Protein Data Bank code 1GYC) showing the three domains and the T1, T2 and T3 copper sites. (**b**) Sequence alignment of TvL and CuL: Amino acid residues in agreement with the consensus are colored based on polarity (positive charge: blue; negative charge: red; polar uncharged: green; apolar uncharged: orange); important structural features are highlighted below or above the sequence: copper-binding sites (brown), substrate-binding residues (orange), and flexible loops close to the substrate-binding site (red).

Fungal laccases contain four catalytic Cu atoms *viz*. the T1 Cu and the tri-nuclear Cu cluster (T2 Cu, T3α Cu and T3β Cu) at the T2/T3 site^3,4,16^. The substrates are consecutively one-electron-oxidized at the T1 site near the protein surface and the 4-electron reduction of O_2_ to water occurs at T2/T3 site that is buried within the protein^2,6,16,17^. Fungal laccases are extracellular and monomeric glycoproteins with ~520–550 amino acids and a typical weight of ~60–70 kDa in their glycosylated form^9,18^. These laccases contain an N-terminal signal peptide sequence of 20–22 residues^18^. Structurally, they consist of three tightly arranged cupredoxin-like domains, each of which possesses β-barrel symmetry^3,9,19,20^. The T1 Cu is situated in domain 3 close to the protein surface, and T2 and T3 (α and β) Cu atoms are present at the interface of domains 1 and 3 (Fig. 1a)^6,21^.

Some plant laccases are involved in lignin biosynthesis, whereas in bacteria and fungi they may be involved in lignin degradation^6,22^. Fungal laccases belonging to the *Basidomycota* division (white-rot fungi) are of particular importance in this regard^6^. Two widely studied white-rot fungal laccases are *Trametes versicolor* (TvL) and *Cerrena unicolor* laccase (CuL). They are sequentially and structurally similar with 68% sequence identity and a structural root mean square deviation (RMSD) of 0.25 Å, Fig. 1b. Many substrate reactions has been studied for TvL^7,11,23–28^ and also some for CuL^29^. 2,2′-azino-bis(3-ethylbenzothiazoline-6-sulphonic acid) (ABTS), syringaldazine (SGZ), catechol, dopamine, 2,6-dimethoxyphenol (DMP), vanillic acid, and syringic acid are substrates of TvL with available K_m_ data^7,24–28^. Sulistyaningdyah *et al*.^23^ studied the relative activities of TvL, *Myrothecium verrucaria* and *Trametes* sp. Ha-1 laccases on phenols, anilines, and ABTS. In another study, K_m_ values were reported for the activity of CuL toward phenols, acids, ketones, amines and phosphates^29^. Structure-guided computational design of laccases using both classical and quantum mechanical approaches is a viable and very useful path to discover new proficient laccases for turnover of specific substrates^30,31^. Quantitative models trained on actual turnover data would in principle offer more predictive accuracy, but depend critically on systematic experimental data on substrate turnover rates for their validation. The TvL and CuL data studied here are the most complete systematic data that we could identify, and we hypothesized that a comparative modeling study might provide new insight into the structure-activity relations of these well-studied enzymes.

To investigate whether this is possible, we explored the quantitative relationship between the binding score obtained from standard docking protocols and from more advanced MMGBSA (Molecular Mechanics Generalized Born Surface Area) computations and the reported relative activities and K_m_ values. Quantitative structure-activity relationship (QSAR) models were derived both from the conformations of the free ligands and the protein-bound ligands to identify the dependency of the results on the conformation of the substrates. Both proteins were also studied by Molecular Dynamics (MD) simulations to explore the dynamics of the binding sites and the overall dynamic stability of the enzymes in relation to our quantitative models.

## Materials and Methods

### Sequence analysis and hydrophobicity plots

Sequence alignment was performed using the Geneious software, version 10.2.3 (http://www.geneious.com)^32^. We applied the CLUSTALW alignment option^33^ with standard parameters. The accession numbers of the used sequences are ALE66001.1 for CuL and Q12718 for TvL. The overall laccase consensus was created by aligning 924 sequences of laccases downloaded from BioCatNet LccED v6.4 (12/01/2017)^34^. Hydrophobicity was estimated using the Kyte & Doolittle scale^35^ as calculated using the ProtScale tool of the ExPASy server^36^.

### Datasets used

The laccases are generally thought to follow Michaelis-Menten kinetics, and K_m_ data are thus available as discussed above^37^. Three datasets (named A, B and C) were used in the present study (Table 1). Dataset A is the smallest and is comprised of 11 well-known, structurally diverse laccase substrates with experimental K_m_ values determined for the commonly studied TvL^7,24,28^. This dataset includes four in-house evaluated compounds^37^. Dataset B consists of 15 congeneric phenolic substrates and one amine whose relative activities toward TvL were reported by Sulistyaningdyah *et al*.^23^. Ligand dataset C comprises 23 substrates with K_m_ values measured for CuL as reported by Polak *et al*.^29^. These datasets with their PubChem compound ID^38^ are presented in Supplementary Tables S1–S3. The K_m_ values of the datasets A and C were reported at low pH (~4.5) and the relative activities of dataset B were derived at comparatively high pH (9.0). To take this into account, it was necessary to test whether the protonation state of the protein at variable pH had any effect on the modeled activity, and thus the ligand states were prepared at both low and neutral pH (4.5 and 7.0) using LigPrep^39^. The K_m_ values (in µM) were first converted into molar units and then expressed as the negative of the 10-base logarithm (pK_m_), and the relative activity values of dataset B were converted into logarithmic values, in order to compare them to the modeled free energy terms.

**Table 1:** Substrate datasets used in the current study.

Dataset	Number of compounds	Protein	Activity values	Class	Series	References
A	11	TvL	K_m_ (derived by different groups)	Phenols, acids, amines	Diverse	^7, 24, 28, 37^
B	16	TvL	Relative activity (derived by same group)	Phenols	Congeneric	^23^
C	23	CuL	K_m_ (derived by same group)	Phenols, carboxylic acids, ketones, amines, phosphates	Diverse	^29^

### Preparation of laccase-substrate complexes

The crystal structure of TvL with code 1GYC was retrieved from the Protein Data Bank (PDB)^21,40^. A homology model of CuL was built based on 1GYC using the Prime Homology Modeling tool of Schrodinger^41^. The structure was validated by analyzing the associated Ramachandran plot. The binding states of both these proteins were prepared using the Protein Preparation Wizard in Schrodinger^42^. Water molecules within 5 Å of the Cu atoms were retained, and all other water molecules were removed. The crystal structures are presumably in the resting oxidized (RO) state of the protein (Supplementary Table S4). This state is thermodynamically stable and plausibly (or together with another fully oxidized catalytic state) involved in electron transfer upon contact with substrates^2,4^. Another fully oxidized native intermediate (NI) state may be the catalytically relevant active state; in the presence of sufficient reductant this state can convert to the fully reduced state of the protein^4^. However, when excess reductant is not available, NI slowly converts to the RO state^4^.

The protonation states of TvL and CuL were prepared at neutral pH (7.0) and low pH (4.5). Asp-206 is a crucial residue involved in the substrate binding and may be protonated (Ash) at very low pH. To test the impact of such a protonation on substrate binding, a TvL structure was prepared at pH 4.5 with this residue specifically protonated (Supplementary Table S5). The detailed oxidation state of the T2/T3 coppers will not affect the substrate binding since they are 12 Å or more from the substrate and the T1 Cu. We speculate that the experimental activity may be best reflected in the 3-electron-reduced protein state considering the expected lower half potential of this state. We thus prepared and studied this state, in which T2 and T3 Cu atoms were reduced and T1 Cu was oxidized. This state was also studied for both proteins (TvL and CuL) at pH 7.0, pH 4.5 and pH 4.5 with Ash 206 (Supplementary Table S5) and subjected to MD simulation to identify whether the protein oxidation state affects the dynamics of the substrate binding sites.

### Molecular docking and MMGBSA analysis

Molecular docking was performed for all substrates using the obtained protein structures (dataset C substrates were docked to CuL, the others to TvL). Five potential binding sites on the proteins were identified using Sitemap^43^, and a 20-Å grid was generated around the T1 Cu site. Substrates were subsequently docked on this grid using the XP scoring function of Glide^44^. For each ligand, a maximum of ten output poses were generated. All these calculations were performed for TvL and CuL at pH 7.0 and 4.5.

Each substrate-protein complex generated by Glide was subject to MMGBSA analysis^41,45^ at six different flexibilities of the protein residues around the ligand: 0 Å, 4 Å, 8 Å, 12 Å, 16 Å, and 20 Å. MMGBSA calculates the binding free energy as the difference between the energies of the minimized protein-ligand complex and minimized ligand plus minimized protein. The binding modes of the substrates at the T1 Cu binding pocket were analyzed. The docking scores and MMGBSA binding energies were compared with experimental relative activities and K_m_ values in order to understand the substrate binding quantitatively and structurally. Probably because quantitative experimental data are scarce, this is, as far as the authors know, the first study that quantitatively correlates K_m_ and relative activity values of substrates of laccases with the modeled docking scores and MMGBSA binding affinities.

### QSAR modeling

In order to identify the crucial substrate properties that explain experimental activities, QSAR modeling was performed^46–49^ using both the free and bound ligand conformations for computing the various descriptors. The free and the bound ligand states were generated by LigPrep^39^ and MMGBSA^41^, respectively. The ligands were prepared at pH 7.0 for dataset B and 4.5 for dataset C. To account for the shape, solubility, binding, and e^−^ transfer mechanism, three types of descriptors were generated: ADMET (Absorption, Distribution, Metabolism, Excretion and Toxicity), semi-empirical quantum mechanical (SE), and quantum-mechanical (QM) using QikProp^50^, NDDO (Neglect of Diatomic Differential Overlap)^51,52^ and Jaguar^53,54^ of the Schrodinger suite^55^. In Qikprop, in addition to the ADMET parameters, the ionization potential (IP) and electron affinity (EA) were also calculated using the semi-empirical PM3 method^50^. We hypothesized that the electronic descriptors of the substrates are important for explaining real laccase activity due to the involved electron transfer from the bound substrate to T1 of the laccase.

QM single point energy calculations were carried out using Density Functional Theory (DFT) with the B3LYP functional and the 6–31 G** basis set^56^. Dataset B contains three anions, which were excluded from B3LYP calculation as the orbital energies are unreliable. Similarly, dataset C contains mainly ions and therefore, these properties were not calculated for them. However, the solvation energy could play a role in defining the enzyme’s K_m_ for these substrates and was thus calculated using QM. Some additional QM parameters were manually calculated using the ligand energies determined by Jaguar, which included IP, EA, electronegativity, hardness, chemical potential and the energy gap between the highest-occupied and lowest unoccupied molecular orbitals (HOMO and LUMO)^57^. These QM properties plausibly play an important role in determining the electron transfer rate once they are bound to the laccase. Semi-empirical parameters were calculated using the RM1 method^52,58^. In addition to the above descriptors, the MMGBSA binding energy and the ADMET volume to solvent accessible surface area (SASA) ratio, which measures shape, were also included in the total descriptor set to probe requirements with regards to substrate fit within the binding site. For dataset C, the experimental oxidation potential (E_o_) reported by Polak and coworkers^29^ was included as an independent descriptor, as it may be assumed to be important for the activity of laccase substrates. The total driving force depends on both the substrate and laccase half potentials, viz. the Nernst Equation, and the e^−^ transfer rate may be affected by this driving force as well, as e.g. seen from Marcus theory^4^.

It became clear quickly that dataset A is too diverse and small. This was observed during MMGBSA binding affinity analysis, which revealed a very low R^2^ of 0.10, a high p-value of 0.34, and a diverse scatter of computed binding affinity vs. pK_m_. Therefore, this dataset was not used further for QSAR studies. For dataset B (16 compounds) and dataset C (23 compounds) all the compounds were used in the respective training sets. Multiple Linear Regression QSAR models were developed using Strike focusing on models with a maximum of three descriptors^59^. In this way, we developed global, ADMET, QM and SE QSAR models. The global models include descriptors calculated by Jaguar and QikProp, whereas the ADMET, QM and SE models contain descriptors from QikProp, Jaguar, and NDDO, respectively. The quality of the QSAR models was evaluated using the correlation coefficient (R^2^) and cross-validated R^2^ (Q^2^). Q^2^ values were derived by randomized leave-one-out analysis as implemented in Strike^59^. In addition, because Q^2^ only reflects internal dataset consistency, we also performed an explicit external validation of the predicted trend for some recently published data as described below.

The use of both the free and the bound ligand conformations, the use of electronic QM descriptors to model redox activity, the use of the MMGBSA binding energies to model K_m_ and relative activity make our study different from conventional QSAR studies applied to protein-ligand interactions. This is because we specifically sought to capture the chemically active conformations that explain the real experimental data for laccases, and these active states most likely relate to the electronic properties of the bound ligand state.

### Molecular dynamics simulations

MD simulations of 12 laccase systems (Supplementary Table S6) were performed for 50 nanoseconds (ns) each, to establish if the dynamics of the substrate binding sites were sensitive to protein type, pH, and protonation of Asp-206. The OPLS 2005 force field was applied^60–62^ and the systems were constructed using the System Builder tool of Desmond^63^. Each protein was solvated with SPC water in an orthorhombic box. Each box volume was ~500,000 Å^3^, with the order of ~47,000 atoms, including ~13,000 water molecules (Supplementary Table S6). The systems were neutralized by adding counter ions (Supplementary Table S6). Initial structure minimization was carried out using a combination of steepest descent and Broyden−Fletcher−Goldfarb−Shanno (BFGS) optimization for a maximum of 2000 iterations. For each protein, 50 ns MD simulation was performed within the NPT ensemble at 300 K and 1.01325 bar, using the standard multistep protocol of Desmond^64^. The integration time-step of 2.0 femtoseconds was used for bonded interactions, and energy and trajectory were recorded at intervals of 1.2 and 50 picoseconds, respectively. Long-range electrostatic interactions were calculated using Ewald mesh summation^65^. The Lennard-Jones potential^66^ was used to determine van der Walls interactions and an interpolating function was used for electrostatic interactions. A cut-off radius of 9.0 Å was used for short-range Coulomb and van der Walls interactions. Pressure and temperature conditions were maintained steady by using the Martyna-Tobias-Klein barostat^67^ and the Nose-Hoover chain thermostat^68^ as is default and commonly applied.

## Results and Discussion

### Sequential and structural comparison of the two laccases

As mentioned in the Methods section, the homology model of the CuL was developed using 1GYC as template, which is a TvL structure. The structural alignment of the CuL model and the TvL structure (1GYC) produced a low RMSD value of 0.25 Å, showing that the two protein models applied are essentially identical in fold structure (Fig. 1a); MD simulation changes this somehow, as expected (see below). The applied CuL model was validated by Ramachandran analysis showing that 98.5% of the residues are in the core and in additional allowed regions, with 1% in the generously allowed region and 0.5% residues in the disallowed region of the plot (Supplementary Fig. S1), which is very acceptable. For comparison, the Ramachandran plot of the experimental laccase structures (from PDB) were analyzed^40^. The laccase structures 1GYC, 2HRG, 2XYB, 1A65 and 1HFU showed 99–100% residues in the core and additional allowed regions, up to 0.7% residues in the generously allowed region and up to 0.2% residues in the disallowed regions of the plot.

Alignment of the TvL and CuL sequences show that the proteins are 68.3% identical. Especially the copper binding sites align perfectly as these parts are evolutionarily conserved, but also the residues involved in substrate binding are found in the same sequence regions (Fig. 1b). We estimate that approximately half of the substrate binding residues are conserved. The flexible loop regions produce most of the variability relevant to substrate binding. However, comparison of both sequences to an overall laccase consensus based on 924 sequences reveals that only three of these residues are conserved: His-393 and Pro-394 in CuL and His-458 in TvL (Supplementary Fig. S2). The histidines are also involved in binding of the copper ions, which explains their high conservation across all analyzed sequences. The Pro-394 adjacent to His-393 is probably involved in stabilization of the secondary structure leading to correct formation of the copper binding site, explaining why it is highly conserved throughout the laccase enzymes. The hydrophobicity of the enzymes is also very similar (Supplementary Fig. S3a). However, as seen in Supplementary Fig. S3b, nine regions differ by >|1| in their hydrophobicity index: regions 5–9, 26–35, 60–74, 178–185, 200–202, 316–324, 349–355, 423–431, and 495–499 (numbering based on consensus sequence of TvL and CuL). Three of these regions lie within or close to flexible loops near the substrate binding site (see MD data below): Region 178–185 is part of the first loop, region 200–202 is part of a second loop, and region 349–355 is very close to a third loop (Fig. 1b).

### Diversity of substrate binding conformations within laccases

**Figure 2:**
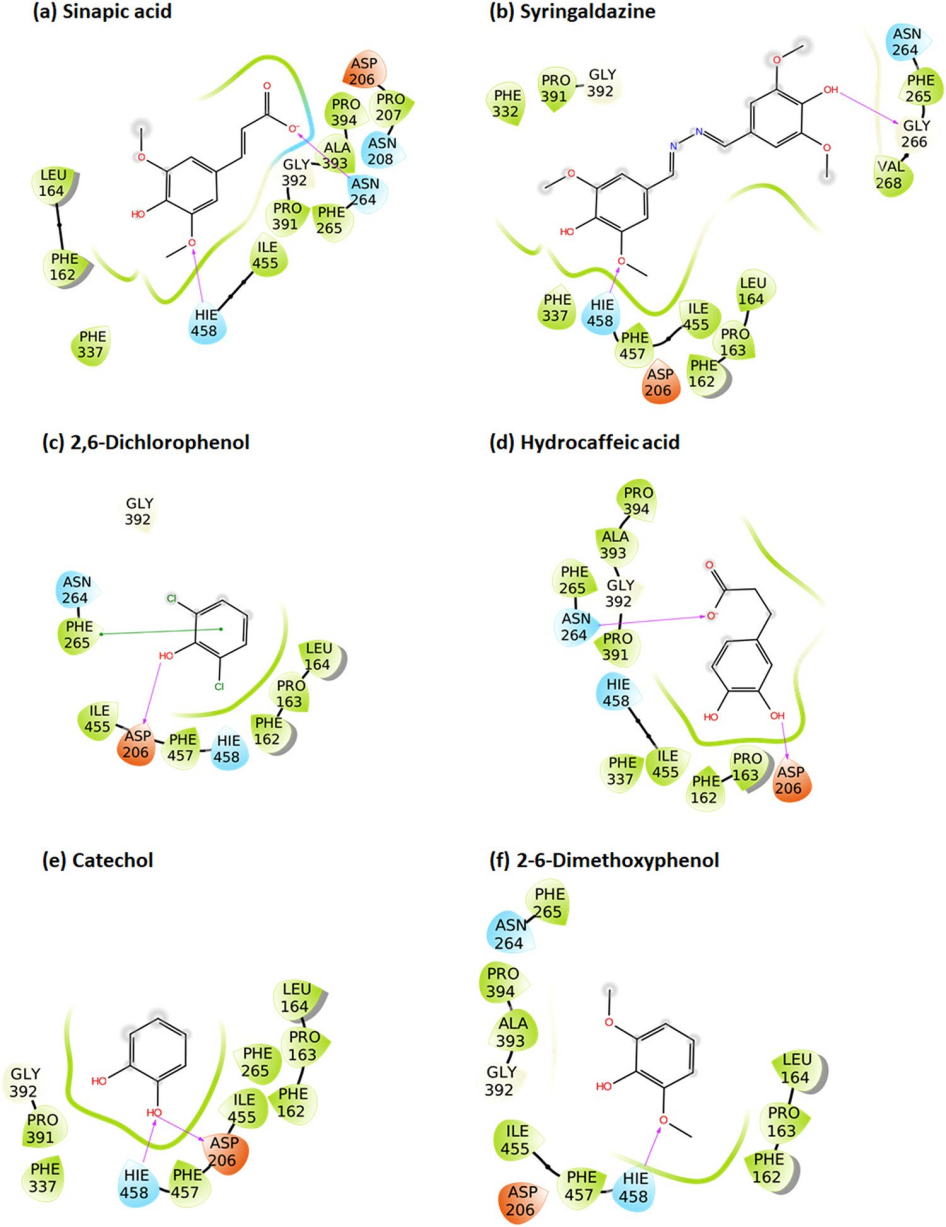
Binding modes of representative compounds of the TvL datasets A and B. Sinapic acid (**a**) and syringaldazine (**b**) are dataset A compounds. Compounds from (**c**) to (**f**) are dataset B substrates.

**Figure 3:**
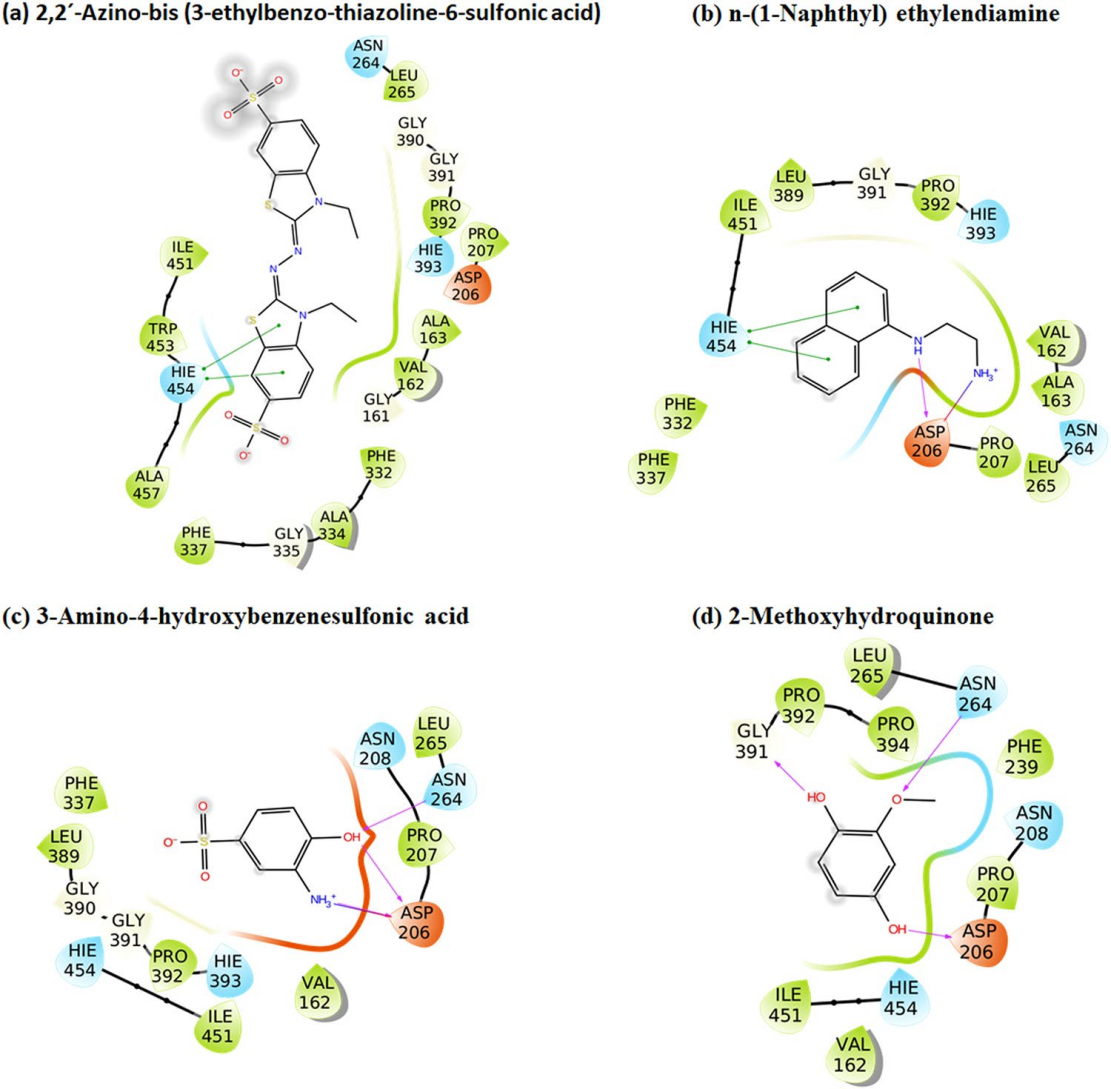
Representative bound conformations of substrates binding to CuL (dataset C).

All the studied ligands were assumed to bind near the T1 Cu, which is known to be the site of substrate binding and oxidation^2,4,6,69^. According to the theory of electron transfer, the proper orientation of the substrates probably involves a short distance between the donor and acceptor T1 site to enhance the e^−^ transfer rate^4^. We have previously suggested that laccase substrates need to have the atom to be oxidized close to T1 in the conformation that reflects experimental turnover, because the electron transfer rate decreases exponentially with the distance between the substrate donor orbitals and the T1-His acceptor orbitals^69^. Accordingly, the relevant active substrate-conformations should be selected for minimal distance to T1, a principle that we consider important to future laccase design^69^. The electron donor atom of the various substrates vary substantially and in some substrates such as ABTS, it is represented by a delocalized electron density on multiple atoms. Larger substrates such as ABTS and SGZ were found to be somewhat solvent-exposed. Most of the substrates interact with His-458 and Asp-206 (TvL numbering) residues and form hydrogen bond, salt bridges, or π-π stacking interactions with them (Fig. 2 and Fig. 3 and Supplementary Tables S7–S9).

It has been reported that His-458 (TvL numbering) and Asp-206 residues form crucial interactions with laccase substrates, with the N^ε^ H-atom of His-458 plausibly involved in the actual electron transfer. Asp-206 forms stable hydrogen bonds with the substrate to maintain this close-encounter active conformation with minimal electron transfer distance^6,69^. Because of this, we focused on ligand conformations that involved interactions with these residues and had a short distance between His-458 N^ε^ and the supposed e^−^ donor atoms. We assumed OH was the donor group of all phenolic substrates and analyzed the distances between the closest OH and His-458. For amines, the distance between NH_2_ and His-458 was calculated, and for other substrates, we performed visual inspection of the docked poses and selected the poses that were closest to the T1 site.

For ligand dataset A, which includes phenolic substrates, Glide and MMGBSA both produced relevant conformations that fulfil the distance requirements of e^−^ transfer, with distances <5 Å (except for *p*-coumaric acid) between the expected electron donor atom and His-458 N^ε^ H-atom (TvL) (Fig 2a,b and Supplementary Table S7). Out of the 11 compounds, nine were phenols, and in seven of these, a methoxy group was present ortho to the phenolic OH. Interestingly, we observed that the methoxy O-atom has a tendency to form hydrogen bond interactions with the N^ε^ H-atom of His-458, which is plausibly involved in the electron transfer pathway. In OH-dilignol, the methoxy group of the methoxyphenyl ring formed a hydrogen bond with His-458. In catechol and dopamine, where OH was present ortho to the phenolic OH, the hydroxyl groups had a tendency to form a hydrogen bond with Asp-206 and His-458. However, when a methoxy or hydroxyl group was absent in the ortho position of phenolic OH (*p*-coumaric acid), the hydrogen bond interaction with His-458 was missing and the distance between the phenolic O-atom and the His-458 N^ε^ H-atom was ~6 Å. We also observed that Asn-264 and Phe-265 are important TvL residues involved in hydrogen bonding and π-π stacking to a variable extent depending on the nature of the substrate.

Of the 16 compounds in dataset B, 15 are phenols and one is amine (*p*-toluidine). In 14 of these compounds, the phenolic OH group formed a hydrogen bond with Asp-206 as the hydrogen bond acceptor (Fig. 2c–f and Supplementary Table S8), strongly suggesting that this is the predominant active conformation of phenolic laccase substrates. We consider this consistently recurring interaction mode of general relevance for the real turnover rate catalyzed by the laccases as discussed further below. The NH_2_ group of *p*-toluidine also formed a hydrogen bond with Asp-206.

We also found that the N^ε^ H-atom of His-458 acted as a persistent hydrogen bond donor and participated in hydrogen bonding with the hydroxyl group of catechol, guaiacol, o-cresol, pyrogallol, and *p*-hydroxybenzoic acid. His-458 also formed a hydrogen bond with the methoxy group of 2,6-dimethoxyphenol. In all these ligand poses, the distance between phenolic OH and the His-458 N^ε^ H-atom was <5 Å. As a final recurring feature in many of the docking simulations, Phe-265 commonly formed π-π stacking interactions with the phenyl ring of many substrates including 2,6-dichlorophenol, 2,4-dichlorophenol, hydroquinone, caffeic acid, *o*-cresol and *p*-hydroxybenzoic acid.

For the CuL substrates of dataset C, we found, interestingly, similar poses, which indicates that the phenolic substrate conformations are generically important. One main difference was that His-454 (which is equivalent to TvL His-458) commonly formed π-π stacking interactions with the phenyl ring of the substrates (Fig. 3 and Supplementary Table S9). Out of the 23 substrates, 11 formed π-π stacking contacts with His-454. However, as TvL, CuL is also engaged in hydrogen bonding with the substrates, in this case with methoxy and phosphate groups of 2-amino-3-methoxybenzoic acid and sodium-1-naphtyl phosphate, respectively. Asp-206 was involved in hydrogen bonding and salt-bridge interactions with the ammonium groups of the twelve ligands: n-(1-naphthyl) ethylendiamine, 3-amino-4-hydroxybenzenesulfonic acid, epinephrine, 3-(3,4-dihydroxyphenyl)-L-alanine, norepinephrine, 2-amino-3-methoxybenzoic acid, 2-aminophenol, 3-aminobenzoic acid, 4-aminophenol, 4-aminobenzoic acid, anthranilamide, and anthranilic acid. Asp-206 also participated in hydrogen bonding with the phenolic OH groups of D-catechin, 3-amino-4-hydroxybenzenesulfonic acid, 2-aminophenol, 4,5-dihydroxy-1,3-benzene-disulfonic acid and 2-methoxyhydroquinone substrates, as we saw for TvL. Furthermore, Thr-168, Asn-264, Leu-265, and Gly-391 consistently formed hydrogen bonds with the substrates.

### Quantitative analysis of the docking scores and activities

In order to determine whether docking scores aid in understanding laccase activity, we attempted to correlate the XP docking scores from Glide with the experimentally reported K_m_ values and relative activities. For all three datasets, we found no significant correlations (Table 2). Glide is well-known for producing excellent conformations of bound substrates in proteins^44,70–72^ but it commonly fails to quantitatively rank the relative binding free energies of ligands, and thus this negative result was not unexpected^73^. Our hypothesis that K_m_ correlates with the binding free energy of the substrate in its active conformation is thus not supported by the Glide scoring, but this could also be due to the known weakness of the scoring functions.

**Table 2:** Correlation of experimental activities (log (activity)/pK_m_) with docking scores and MMGBSA binding energies at different pH conditions.

Ligand dataset	Protein/state	pH	Docking R^2^ (p value)	MMGBSA at various protein flexibilities around the ligand R^2^ (p value)					
			Glide XP	0 Å	4 Å	8 Å	12 Å	16 Å	20 Å
A	TvL/RO	7	0.02 (0.68)	0.05 (0.50)	0.04 (0.57)	0.07 (0.43)	0.06 (0.49)	0.03 (0.60)	0.05 (0.53)
		4.5	0.09 (0.36)	0.10 (0.34)	0.07 (0.45)	0.04 (0.55)	0.04 (0.58)	0.05 (0.51)	0.02 (0.68)
B	TvL/RO	7	0.001 (0.91)	0.29 (0.03)	0.16 (0.13)	0.09 (0.27)	0.06 (0.35)	0.06 (0.38)	0.04 (0.47)
		4.5	0.01 (0.79)	0.13 (0.17)	0.16 (0.12)	0.05 (0.41)	0.09 (0.25)	0.08 (0.28)	0.002 (0.88)
C	CuL/RO	7	0.05 (0.31)	0.20 (0.03)	0.31 (0.01)	0.30 (0.01)	0.19 (0.04)	0.15 (0.07)	0.19 (0.04)
		4.5	0.01 (0.71)	0.21 (0.03)	0.18 (0.05)	0.33 (0.004)	0.19 (0.04)	0.17 (0.05)	0.09 (0.16)

**Figure 4:**
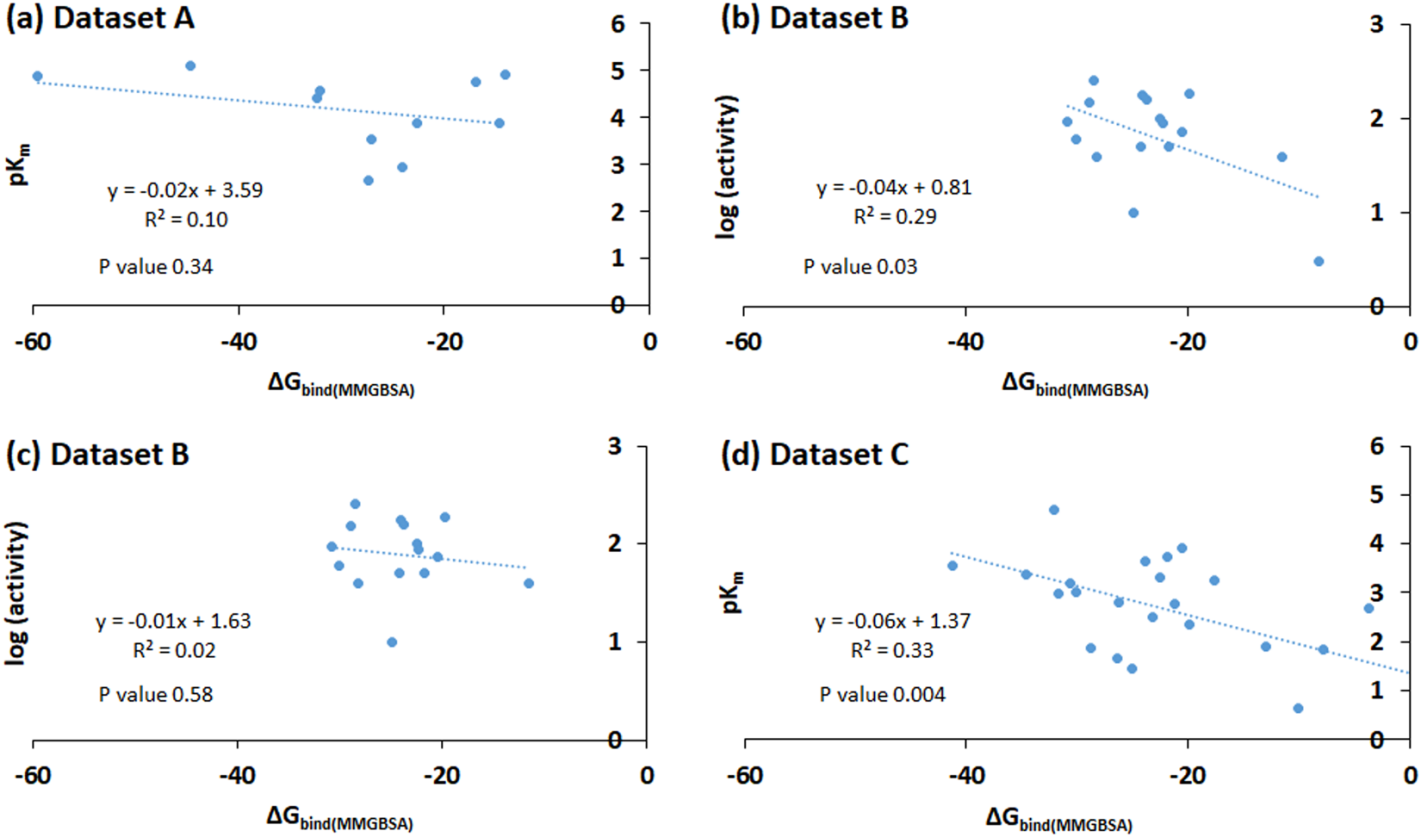
Plots of the maximum correlation observed for the three datasets (A, B and C) between ∆G_bind(MMGBSA)_ (kcal/mol) and pK_m_ or relative reported activity. (**a**) Dataset A compounds showed a R^2^ value of 0.10 at pH 4.5 and 0 Å flexibility. (**b**) For dataset B, a maximum R^2^ value of 0.29 was observed. (**c**) When the outlier compound *p*-hydroxybenzoic acid was removed from the dataset B, the correlation was reduced to 0.02. (**d**) The dataset C showed the highest R^2^ value of 0.33 at pH 4.5 with 8 Å protein flexibility.

Therefore, we also carried out more advanced and typically more accurate MMGBSA binding energy calculations (in kcal/mol)^74^ using six different degrees of protein flexibility for the three datasets and analyzed the correlation with the experimental data (Table 2, Fig. 4, and Supplementary Fig. S4–S9). We performed a comparative analysis of the MMGBSA scores at two pH i.e. 4.5 and 7.0. Interestingly, as a general trend, the correlation increased with a decrease in the protein flexibility (i.e. how much of the protein is allowed to be flexible and adjust upon substrate binding). For dataset A, a maximum observed R^2^ of 0.1 was seen at pH 4.5 when no flexibility was assigned to the protein (Fig. 4a). At pH 7.0, no correlation was observed for dataset A. A good correlation was not expected as this dataset contains structurally diverse compounds and the K_m_ values were derived by different research groups, which introduces heterogeneity and noise into the dataset. However, the direction of the correlation follows the expected outcome, i.e. as the pK_m_ increases, the MMGBSA score becomes more negative.

For dataset B, we found a good correlation at smaller flexibility, with a maximum correlation of R^2^ = 0.29 at pH 7.0 (0 Å flexibility). The correlation was more pronounced than at pH 4.5 (R^2^ = 0.16 at 4 Å flexibility), and the direction of correlation was meaningful. The low R^2^ at pH 4.5 possibly reflect that the experimental activities were reported at pH 9.0. At pH 7.0 (0 Å flexibility), *p*-hydroxybenzoic acid was an lone point in the data range; if removed, the entire correlation was lost (R^2^ = 0.02, Fig. 4b,c). Thus, this correlation is very dependent on only one data point because of an uneven coverage within the data range, and we can therefore not trust this observation very much, although we note that the direction of the correlation is meaningful (stronger binding reflects smaller K_m_). The experimentally reported relative activity values are plausibly approximately equivalent to k_cat_/K_m_ under Michaelis-Menten conditions of an assay, and thus we would expect binding affinity to only explain part of this activity (the remaining part being explained by the electron transfer properties affecting the rate).

For dataset C, we found that the binding free energy estimated by MMGBSA and the experimental pK_m_ values correlated significantly (p < 0.05) at lower flexibility distances, with a maximal correlation of R^2^ = 0.33 at pH 4.5 using 8 Å flexibility (Fig. 4d). Similar correlations were also observed at pH 7.0 using 4 Å (R^2^ = 0.31) and 8 Å (R^2^ = 0.30) protein flexibilities. Here, we observed very similar R^2^ values at pH 4.5 and 7.0, with the actual K_m_ observed at pH 4.5. The pK_m_ values increased (K_m_ decreased) with decrease in the binding energy, as expected if smaller K_m_ reflects stronger substrate-enzyme binding. We therefore conclude that the MMGBSA binding free energy is a very valuable descriptor of experimental K_m_values, but the descriptor is less important for explaining relative activity, which also depends on features that contribute to k_cat_. The lower data quality may explain some of the poor performance of datasets A and B in Table 2. We also conclude that low flexibility that maintains the protein geometries close to the crystal structure explain the experimental data better than computationally relaxed proteins, although the optimal protein relaxation depends on the substrate. Dataset B contains smaller substrates (phenol analogs) and therefore favors little protein relaxation. In contrast, Dataset C includes large compounds such as sodium-1-naphtyl phosphate, *n*-(1-naphthyl) ethylendiamine, D-catechin and ABTS, and therefore, prefers some protein flexibility for proper alignment and removal of steric clashes.

### QSAR models of laccase activity

In order to determine whether laccase activity can be rationally predicted and explained, QSAR was performed for dataset B and C compounds that contained most data and were derived from only a single laboratory each, which is expected to reduce noise; the fact that dataset C is most well-behaved partly emerges already from the MMGBSA study of dataset C vs. A (both are K_m_ data but only dataset C shows good correlation). Binding affinity is generally thought to contribute to observed K_m_ values, as confirmed above for dataset C, and thus the MMGBSA binding free energy was included as a descriptor during QSAR modeling. Because the binding poses within the protein may represent essential structural information that differs from the free ligand state, as required for the e^−^ transfer and activity, both free and bound ligand conformations were used for computation of the descriptors. The comparison of QSAR results obtained with free and bound conformations is of technical interest on its own as it outlines the importance of generic ligand features vs. features specific to the protein-ligand complex.

**Figure 5:**
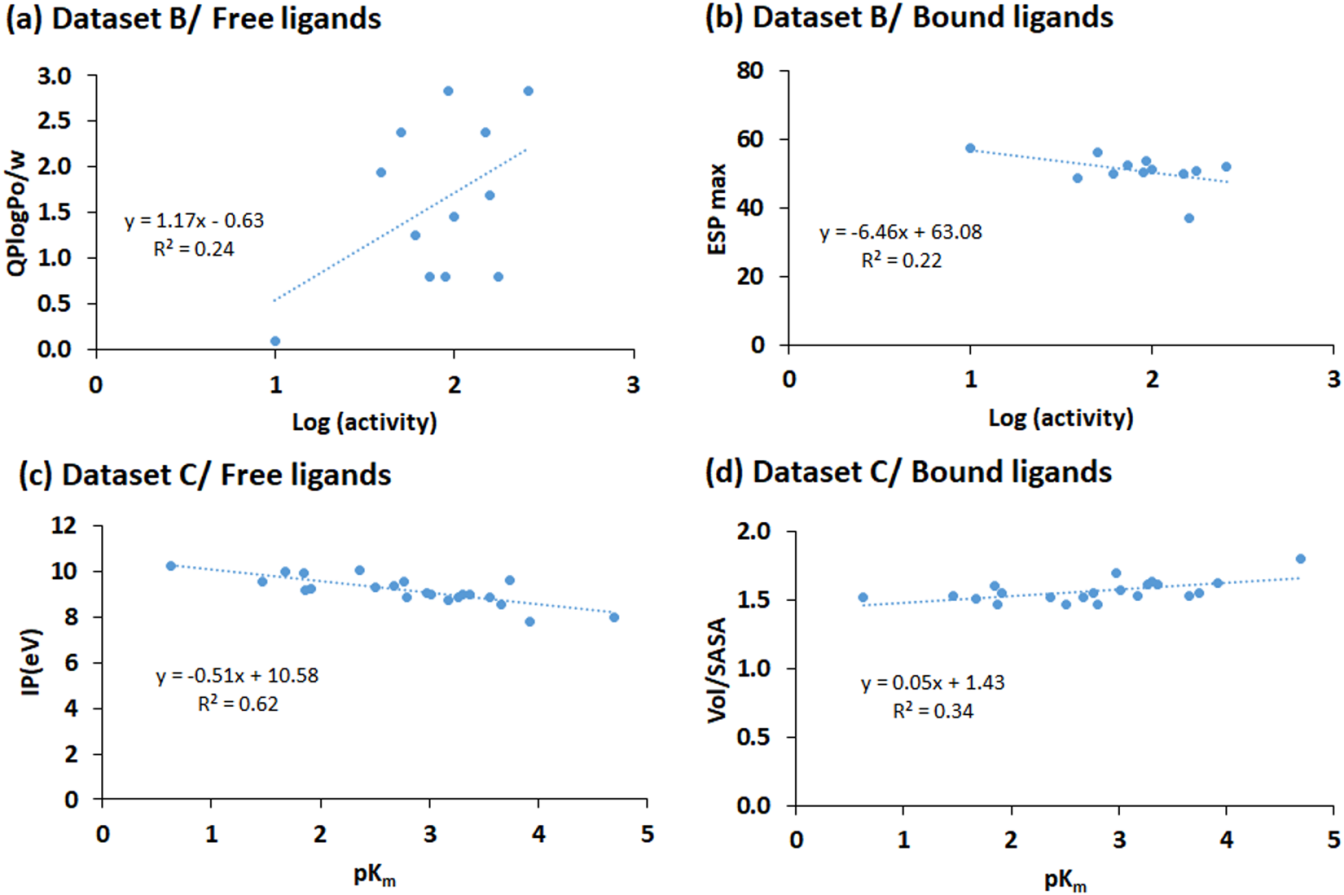
Scatter plots of the descriptors (computed values without normalization) of the QSAR models showing significant correlation (>95% confidence) with log(activity) or pK_m_. (**a**) QPlogPo/w found in the QSAR of free ligand states of dataset B. (**b**) ESP max showed a better correlation with log (activity) of dataset B when using the bound state than when using free ligand states. (**c**) IP (in units of eV) displayed a good correlation with pK_m_ of dataset C. (**d**) Vol/SASA of the bound state of dataset C compounds showed a good correlation with pK_m_.

All the QSAR models developed in the present study are shown in Supplementary Table S10. The descriptors of these models are not normalized; however, the descriptors of the models discussed in the present study (models 1 to 7 discussed below) are normalized. These descriptors are explained in Supplementary Table S11, and the scatter plots of the correlation between these descriptors (without normalization) and log (activity) or pK_m_ are shown in Supplementary Fig. S10–S15. The representative descriptors showing high correlation with log (activity) or pK_m_ are shown in Fig. 5. Toluidine, which is an amine, was present in dataset B, which otherwise consisted of phenols. Accordingly, it was found to be an outlier in the QSAR modeling, i.e. its inclusion made the dataset too noisy. Therefore, for global, QM and SE models of dataset B, the number of data points in the training set was n = 12 and for ADMET models, n = 15.

The best global QSAR model using the free ligands of dataset B was (R^2^ = 0.76; Q^2^ = 0.57; standard error = 0.21):

1 $$\mathrm{log}\,({\rm{activity}})=1.38+1.36\,{\rm{QPlogPo}}/{\rm{w}}+0.88\,{\rm{QPlogS}}-0.97\,{\rm{ESP}}\,{\rm{\max }}$$

In model 1 (Eq. 1), the maximum value of the electrostatic potential energy (ESP max, measured in kcal/mol) was an important descriptor; it correlated inversely with the reported activity of dataset B. In addition, the hydrophobicity of the ligands as measured by the common octanol-water partition coefficient (QPlogPo/w) and the water solubility (QPlogS) showed positive regression coefficients in the QSAR model. The QPlogPo/w had a major effect with R^2^ = 0.24 with log (activity), whereas QPlogS showed only a small contribution towards activity. The highly active catechol has the highest QPlogS value, and the weakly active pyrogallol has low solubility. The fairly active compounds catechol, hydroquinone, and resorcinol (with two phenolic OH groups) exhibited both hydrophobicity and water solubility, whereas the much less active compound pyrogallol (with three phenolic OH groups) did not. This suggests that a good laccase substrate should exhibit an optimal balance of hydrophobicity and hydrophilicity.

In addition, a 3-descriptor ADMET model was derived (R^2^ = 0.74; Q^2^ = 0.50; standard error = 0.30):

2 $$\mathrm{log}({\rm{activity}})=3.6-1.89\,{\rm{FISA}}-2.64\,{\rm{glob}}+1.41\,{\rm{QPlogS}}$$

Model 2 (Eq. 2) includes the hydrophilic solvent accessible surface area (FISA), the globularity of the substrate (glob), and QPlogS as descriptors. FISA showed inverse correlation with the experimental activity. As above, it implies that the phenol substrates need to be optimally hydrophobic to maximize activity. As the globularity of the compounds decreases, activity increases, correlating well with the extended nature of the substrate-binding site. The globularity parameter, which is surface area to SASA ratio, showed inverse correlation (−0.93) with the Vol/SASA ratio of the substrates. The solubility parameter QPlogS correlated positively with activity, in good agreement with the above observation for the global model.

It was surprising and encouraging to us that the experimental activity of the phenols of dataset B can be described so well by a simple 3-parameter model; the features driving laccase activity toward phenolic substrates according to this model, hydrophobicity, solubility, shape and the electrostatic potential energy of the substrates, suggests that the substrates require optimum hydrophobic packing and solubility to reach the T1 site, but then additional favorable electronic properties to engage in the electron transfer. This double requirement (hydrophobic association, but also electronic alignment) may be of importance to future optimization of laccase activity toward phenolic substrates, perhaps even including large phenolic constituents of lignin, although this remains to be investigated further.

The above models were obtained using conventional free ligand conformations. As explained we also wanted to study the bound ligand conformations separately. The best general 3-descriptor QSAR model for these conformations was (R^2^ = 0.80; Q^2^ = 0.59; standard error = 0.20):

3 $$\mathrm{log}\,({\rm{activity}})=1.98+0.94\,{\rm{PISA}}+1.00\,{\rm{EA}}({\rm{eV}})-1.23\,{\rm{ESP}}\,{\rm{\max }}$$

Model 3 includes ESP max (as in Model 1), the electron affinity (EA, measured in eV) and the solvent exposed π surface contributed by the carbon atoms, which is also an electronic property of the substrate (PISA). ESP max again exhibited inverse correlation with the experimental activity. In contrast, PISA and EA(eV) exhibited positive correlation, which revealed that as the solvent-exposed π surface of the carbon atoms and attached hydrogen and electron affinity increased, the activity also increased.

The ADMET model 4 (Eq. 4) showed that in addition to QPlogPo/w and QPlogS, Vol/SASA contributes to activity. Vol/SASA showed similar inverse correlation with the globularity (glob) as for the free ligand states. The ADMET model equation was (R^2^ = 0.82; Q^2^ = 0.67; standard error = 0.24):

4 $$\mathrm{log}\,({\rm{activity}})=-\,0.44+1.72\,{\rm{QPlogPo}}/{\rm{w}}+1.87\,{\rm{QPlogS}}+1.91\,{\rm{Vol}}/{\rm{SASA}}$$

We conclude that a slightly better correlation is obtained using descriptors computed for the bound ligands than for the free ligands, but importantly, similar models are achieved in both cases, i.e. our results are not dependent on the approximations made in the ligand conformations. Furthermore, a simple 3-descriptor model can describe the experimental activity of these phenolic laccase substrates well. Thus, electronic properties of the substrates (ESP max), as well as the hydrophobicity (QPlogPo/w), solubility (QPlogS) and shape (Vol/SASA and glob) largely determine the activity of TvL toward phenolic substrates (both in free and bound ligand QSAR models). This should be of interest in future optimization of laccases toward phenolic substrates.

To understand the basis of laccase activity further, we also developed QSAR models using dataset C with known K_m_ values. For free ligand states, sodium-1-naphtyl phosphate (Supplementary Table S3), which exhibited a very high K_m_, was considered an outlier and therefore removed. In the global models, the ionization potential (IP, measured in eV) was identified as the most important parameter (Eq. 5) that showed inverse correlation with pK_m_.

The best models obtained using free ligands and dataset C were:

5 $${{\rm{pK}}}_{{\rm{m}}}=4.43-2.93\,{\rm{IP}}({\rm{eV}});\,({{\rm{R}}}^{2}=0.62;\,{{\rm{Q}}}^{2}=0.53;\,{\rm{standard}}\,{\rm{error}}=0.60)$$

6 $${{\rm{pK}}}_{{\rm{m}}}=2.69+1.99\,{\rm{accptHB}}-2.57\,{\rm{IP}}({\rm{eV}})\,+1.51\,{{\rm{E}}}_{\mathrm{Solv}(\mathrm{PBF})};\,({{\rm{R}}}^{2}=0.77;\,{{\rm{Q}}}^{2}=0.66;\,{\rm{standard}}\,{\rm{error}}=0.49)$$

The solvation energy (E_Solv(PBF)_, computed from the PBF solvent model in kcal/mol) and the number of hydrogen bond acceptors (accptHB) are important parameters for pK_m_ in dataset C (Eq. 6). The solvation energy and number of hydrogen bond acceptors correlated positively with pK_m_. The interpretation of the recommended models 5 and 6 is that the laccase substrates “start” with a favorable generic positive pK_m_ common to all phenols, which is then increased further (in model 6) by favorable hydrogen bonds and dehydration from the water phase. In both models 5 and 6, pK_m_ is impaired by the ionization potential indicating that a large cost of removing the electron impairs observed K_m_, i.e. the electron transfer is entangled with the K_m_ as it only reflects the catalytically active state.

It was again encouraging that the experimental K_m_ of dataset C can be described by simple models. The solvation energy and the number of hydrogen bond acceptors suggest that the substrates require favorable binding at the T1 site; but the importance of the ionization potential suggests that the measured K_m_, in addition to the binding, also carries information about the actual electron transfer at the T1 site, probably because K_m_ values are not solely interpretable (and separable) as binding affinities due to the nature of the Michaelis-Menten kinetics^75^.

Using the bound ligand conformations of dataset C, a 3-descriptor global model was derived (Eq. 7); the experimental oxidation peak (E_o_(expt)), the shape (Vol/SASA) and the hydrophilic surface area (FISA) of the substrates were the important parameters. The best global model equation was (R^2^ = 0.74; Q^2^ = 0.60; standard error = 0.53):

7 $${{\rm{pK}}}_{{\rm{m}}}=3.04-1.14\,{\rm{FISA}}-1.79\,{{\rm{E}}}_{{\rm{o}}}({\rm{expt}})+3.98\,{\rm{Vol}}/{\rm{SASA}}$$

Vol/SASA correlated positively, whereas E_o_(expt) and FISA correlated inversely with pK_m_. The correlation of FISA with pK_m_ was similar to as observed with log (activity) of the dataset B compounds. This model consistently suggests that the oxidation half potential, shape and hydrophilicity of the substrates contribute to the experimentally measured K_m_; the latter two can be related to formation of the active poses engaged in electron transfer, whereas the later relates to an activity effect on the measured K_m_ that cannot be disentangled from the binding affinity.

Our interpretation of these results is that favorable binding of the substrates at the T1 site of laccase is required for exhibiting a good (low) K_m_ value. In addition, the involvement of the ionization potential and oxidation potential of the substrate indicates that the K_m_ also reflects some information on the e^−^ transfer activity occurring at the T1 site, *i*.*e*. *real measured K*_*m*_ *represents an active binding state of the substrate rather than just the average binding affinity*^75^. Furthermore, the observation that simple QSAR models describe the experimental K_m_ values of the laccase for diverse substrates with only a few descriptors is very encouraging. These conclusions may be of the interest in further studies of enzymatic K_m_ optimization in general and laccase optimization in particular.

### External validation of QSAR models

**Figure 6:**
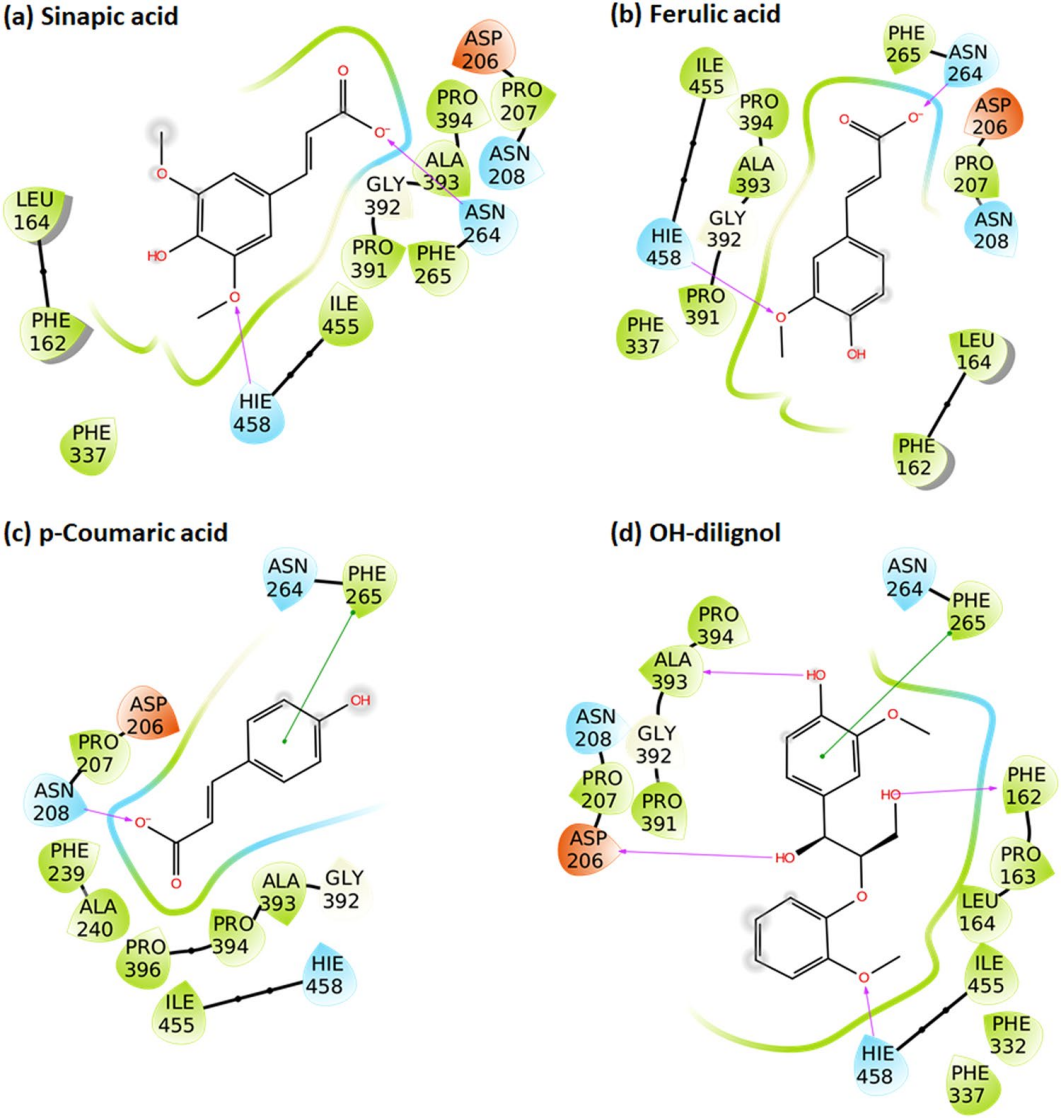
Binding modes of the in-house evaluated substrates.

To test whether our developed models have any predictive value, we compared our model performance against K_m_ values of four substrates previously obtained in-house using TvL under the same experimental conditions^37^. These four K_m_ values were analyzed with respect to the developed binding model and QSAR models. These compounds included sinapic acid, ferulic acid, *p*-coumaric acid and OH-dilignol. The important role of His-458 was clear from this analysis, as already discussed above. His-458 forms a coordination bond with T1 Cu along with two other residues (His and Cys) and helps the T1 Cu to attain a trigonal geometry^6^. Sinapic acid, ferulic acid and *p*-coumaric acid were very similar, differing only by the methoxy group at the ortho position of the phenolic OH (Fig. 6). The methoxy O-atom acts as a hydrogen bond acceptor and forms a hydrogen bond with the H-atom of N^ε^ on His-458. The distance between His-458 N^ε^ and the phenolic OH of these substrates was <5 Å. However, when this methoxy group is absent, as in *p*-coumaric acid, the hydrogen bond interaction with His-458 (TvL) is lost and the distance between His-458 N^ε^ and the phenolic OH increased to ~6 Å. Thus, the absence of the methoxy group in *p*-coumaric acid prevents the substrate in obtaining what we suggest is the active conformation required for e^−^ transfer. This finding is in agreement with our suggestion based on the K_m_ analysis that specific active conformations with short electron transfer distances are responsible for the real observed turnover in laccases.

**Figure 7:**
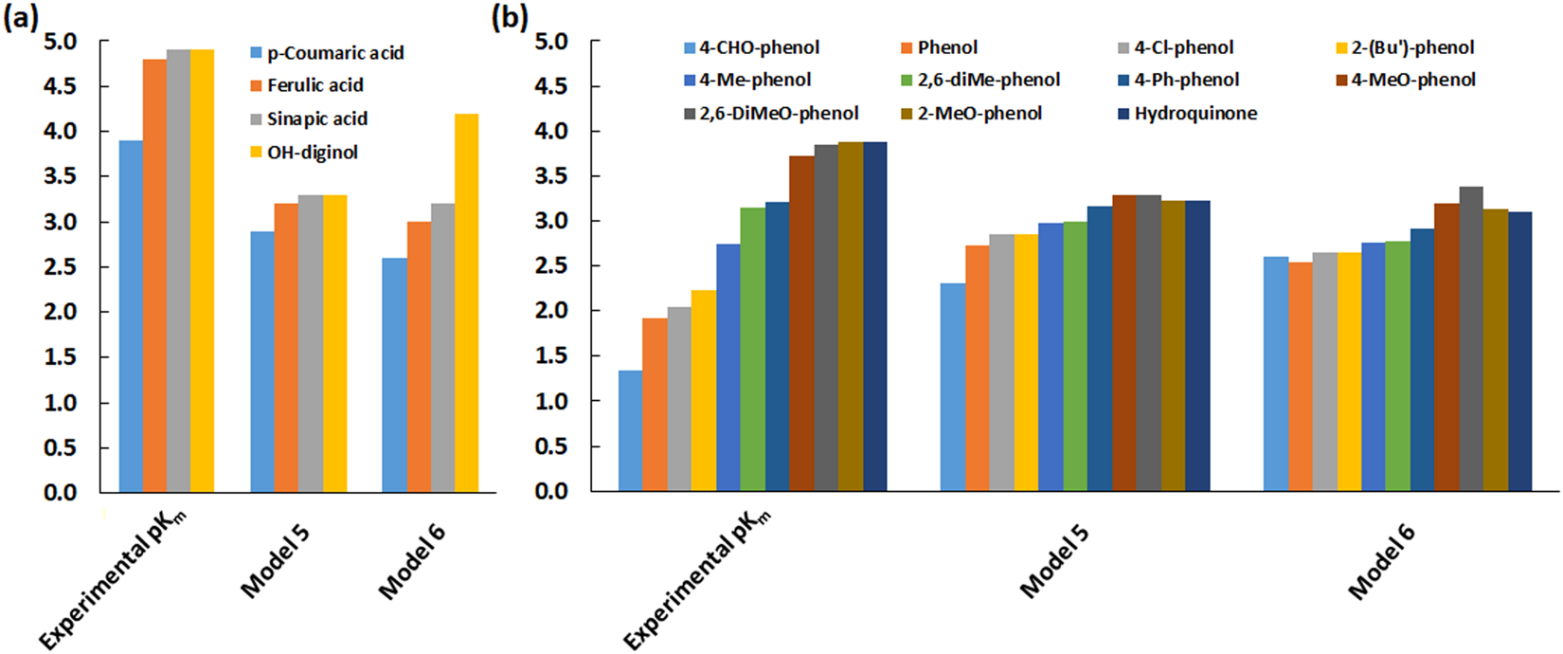
Prediction of the substrate activities using our recommended pK_m_ models 5 and 6. (**a**) Predictions on in-house evaluated compounds. For OH-dilignol, a range of predicted pK_m_ was obtained as shown in Supplementary Table S12. (**b**) Externally validated trend prediction using models 5 and 6 (please see text for details).

The pK_m_ values of the four test compounds were predicted using our recommended pK_m_ models developed using the best-quality dataset C (Fig. 7a and Supplementary Table S12). In model 7, the experimental oxidation potential is a descriptor, whose values were not available for the in-house compounds, and therefore, this model was not used. We found that the predicted and experimental pK_m_ values were in good trend agreement (Fig. 7a and Supplementary Table S12). Considering that the K_m_ values of the in-house compounds were obtained for TvL, this confirms the generality of the models for both the TvL and CuL datasets B and C. The relatively smaller pK_m_ predicted for the *p*-coumaric acid (having the lowest experimental pK_m_) may hopefully indicate the ability of the QSAR models in differentiating substrates based on pK_m_. OH-dilignol was structurally more diverse than the other three compounds of the dataset and therefore, could plausibly be considered as outlier. In this case, the single descriptor QSAR model using only the ionization potential best reproduces the experimental relative pK_m_ of the compounds.

To critically test our models, we also analyzed another external dataset of 12 substrates with reported K_m_ for a laccase from *Trametes villosa* (Fig. 7b and Supplementary Figure S16)^76^, using our recommended pK_m_ models 5 and 6. All compounds in this dataset except 2,6-dimethylaniline are phenolic and thus suitable for testing our models once the aniline was removed (please see Supplementary Figure S16 for results with the aniline included; the models are clearly only suitable for phenolic substrates with the proper hydrogen bonding as explained above). We observed good trend agreement between the actual and predicted pK_m_ values, even though the activities were reported for *Trametes villosa* laccase and the models were developed based on data for CuL, indicating again that local phenolic alignment near T1 is largely generic. We obtain R^2^ = 0.90 and 0.83 for predicted vs. observed pK_m_ values using models 5 and 6, respectively. This confirms that our models can be used for broader predictions of phenolic turnover by laccases.

### Protein-state specific molecular dynamics

**Figure 8:**
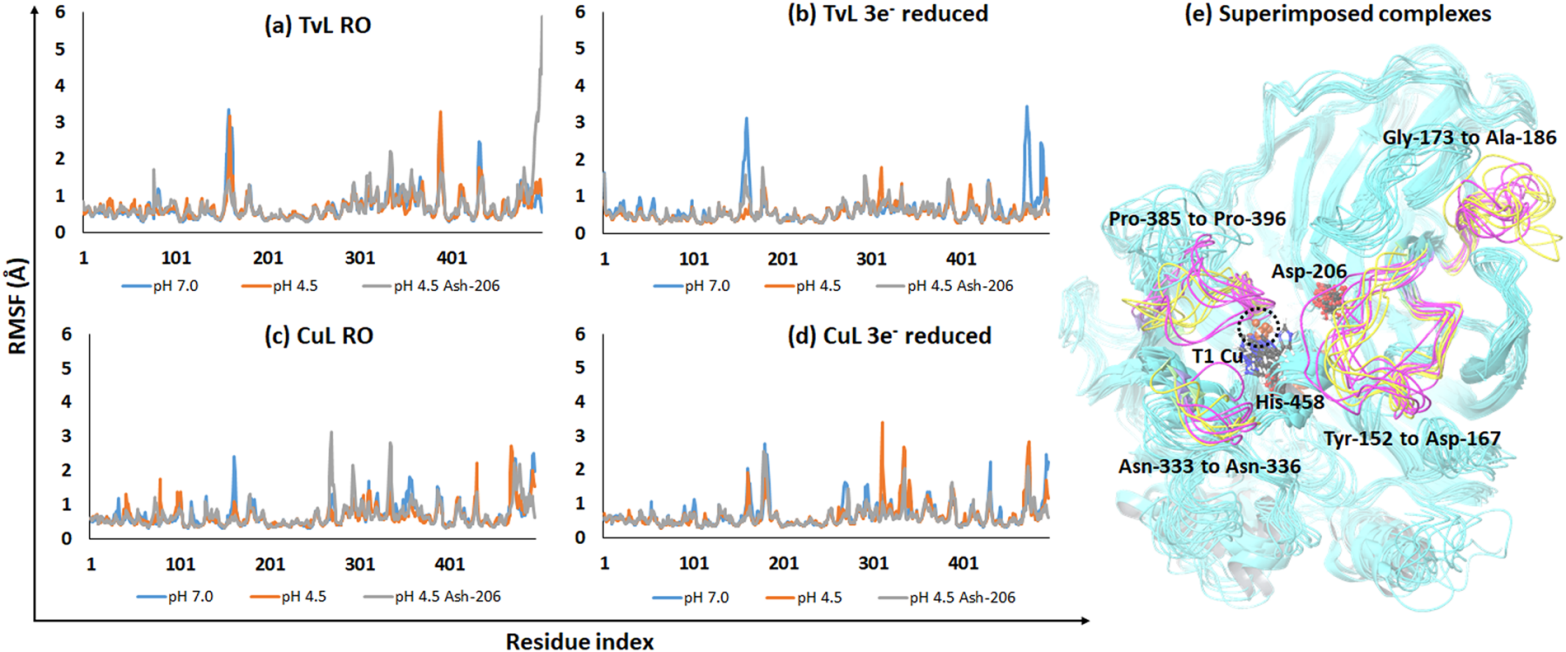
RMSF plots of the residues for the last 20 ns of MD simulations. (**a**) Plot of the RO state of TvL states. (**b**) Plot of the 3e^−^ reduced state of TvL states. (**c**) Plot of the RO state of CuL states. (**d**) Plot of the 3e^−^ reduced state of CuL states. (**e**) Loop regions of TvL (magenta colored) and CuL (yellow colored) states showing high fluctuations during MD and change in conformation in the representative MD structure. Residue numbering is according to the TvL structure (1GYC).

To understand whether protein oxidation and protonation state would change the dynamics and structure of the substrate binding cavities of the two proteins, we performed a series of 12 MD simulations of variable pH and oxidation state (see Methods). All trajectories displayed stable RMSD after 50 nanoseconds (Supplementary Fig. S17), consistent with previous findings for these proteins^77^. Root mean square fluctuation (RMSF) plots and B factors of the residues were analyzed for the last 20 ns trajectory (Fig. 8 and Supplementary Table S13). RMSF plots revealed high flexibility of some loops and the C-terminal residues, as expected. Importantly, conserved residues involved in interactions with the substrates were stable with very low RMSF values.

Asp-206 is located in the conserved loop region with the sequence Ile-Ser-Cys-Asp-Pro-Asn in both TvL and CuL. The residues of this region formed interactions with the substrates as reported above. The MD simulations show that this region is relatively stable in all TvL and CuL states with ≤0.5 Å fluctuations; thus, the importance of Asp-206 in guiding substrate binding discussed above does not seem to be dependent on the dynamics of the proteins and does not change with protein oxidation or pH state.

Asn-264 and Phe-265 form important hydrogen bonding and π-π stacking interactions with substrates in TvL. Asn-264 was conserved in TvL and CuL, whereas Phe-265 was replaced by another hydrophobic residue Leu-265 in CuL. These residues are located in the loop region from Pro-263 to Asn-275, which forms part of the substrate-binding pocket. Except for two residues (TvL/CuL: Phe/Leu265 and Val/Thr268), this loop region was conserved. In TvL, this loop region fluctuated by less than 1.0 Å. However, in CuL, this segment exhibited higher fluctuations with a maximum RMSF seen for Gly-269 (3.15 Å) in the RO state of CuL (pH 4.5 Ash-206).

The TvL segment Ile-455 to His-458 and the corresponding CuL region Ile-451 to His-454 form part of the binding cavity, with His-458 (TvL numbering) involved in hydrogen bonding and π-π contacts with substrates and electron transfer to T1 Cu. This region was dynamically stable and showed RMSF <1.0 Å in all the studied protein states, indicating that our conclusions are not sensitive to the specific protonation and oxidation state of the proteins. The TvL loop region from Pro-391 to Pro-396 and the corresponding CuL segment Leu-389 to Pro-394 also form an important part of the substrate-binding cavity. These regions displayed RMSF values < 1.6 Å. However, the adjacent region Thr-387 to Ala-390 in TvL showed comparatively higher fluctuations (Fig. 8 and Supplementary Table S13).

In order to visualize the structural changes, clustering of the trajectories was applied to the last 20 ns of all 12 MD simulations. The centroid of the most populated cluster was chosen as a representative structure for each MD system. These structures at neutral and low pH were then compared. Considerable differences between low and neutral pH were seen, with RMSD >1.3 Å in both TvL and CuL states (Supplementary Table S14), which reflected a partial change in secondary structural elements and their conformations. This is of interest because experimental structural insight for the same proteins at different pH is not available, although the pH of experimental laccase assays vary substantially. The highest RMSD was observed between TvL RO (pH 7) and TvL RO (pH 4.5 Ash-206), where a major change was observed in the terminal residues, which attained a different conformation in the two structures.

The region Trp-484 to Pro-489 existed as α helix in TvL RO (pH 7), whereas it was a loop in TvL RO (pH 4.5 Ash-206). Similar structural differences were also observed between TvL RO (pH 4.5) and TvL RO (pH 4.5 Ash-206) and when analyzing secondary structures that existed >70% of the last 20 ns (Supplementary Table S13). Similarly, in the 3-electron-reduced state of TvL (pH 7 and 4.5), a major difference occurred in the region from Pro-481 to Leu-494. This segment was helical at pH 4.5, but more disordered (Lys-482 to Ile-490) at pH 7. The loop region Tyr-152 to Asp-167 attained similar conformations in 3-electron-reduced TvL (at pH 4.5 with both protonated and non-protonated Asp-206), but fluctuated at pH 7 (Fig. 8e). This region also fluctuated in TvL RO (pH 7 and 4.5) states. In all CuL states, the loop regions Tyr-152 to Asp-166 and Gly-172 to Ala-186 showed high flexibility and achieved different conformations (Fig. 8e).

We can conclude that the change in the protonation states of laccases (low and neutral pH) affect the dynamics of the protein, the three main loops surrounding the T1 site exhibit variable fluctuations (Fig. 8e), and the C-terminal residues generally show high mobility in all the TvL and CuL systems, as seen before^77^. However, the RO and 3e^−^ reduced states showed similar fluctuations of residues involved in substrate binding, i.e. the change in the copper oxidation states has no major effect on the protein dynamics of the binding sites, consistent with their buried and conserved nature. Thus, our QSAR results, which depend only on residues close to the substrates, will not depend on the protonation and oxidation state of the proteins.

## Conclusions

In this work, we have explored the molecular determinants of laccase activity by using three experimental datasets and a variety of computational chemistry techniques. We explored QSAR properties both for the bound and free conformations of the ligands to test the importance of substrate conformation in explaining the experimental data. As far as we know this is the first attempt to quantitatively correlate experimental activity data of laccases to molecular descriptors. We hypothesized that the electronic descriptors of the substrates are important for explaining real laccase activity due to the nature of the involved electron transfer from the bound substrate to T1 of the laccase.

We find that MMGBSA estimates of the binding free energy correlate with log K_m_ for the largest, most well-behaved dataset. This indicates that K_m_ values at least partly reflect the binding constant, but importantly other features contribute. Most of the docked phenolic substrates (14) displayed a hydrogen bond between the phenolic OH group and Asp-206 in a deprotonated state. We therefore conclude that this is the predominant active conformation of phenolic laccase substrates of importance to future optimization of laccase activity toward such substrates.

The docked conformations of six ortho methoxyphenols to TvL showed a hydrogen bond between the methoxy O-atom and His-458. This residue also formed a hydrogen bond with the same OH group of six phenolic substrates, which were involved in hydrogen bonding with Asp-206. Thus, His-458 partly determines the active conformation of the bound substrates contributing to the observed K_m_ values. All the docked phenolic substrates of dataset B displayed <5 Å distance between the phenolic OH and the His-458 N^ε^ H-atom, as His 458 is the plausible electron acceptor at the T1 site. This indicates that the bound phenolic substrates have active conformations suitable for electron transfer.

We show that simple QSAR models can describe the experimental activity parameters with good and predictive trend accuracy. We conclude that the phenolic laccase substrates mainly require an optimal shape, a good hydrophobic packing and an optimal water solubility to reach the T1 site, and then additional electronic features for the electron transfer. Our MD simulations show that changes in T2/T3 oxidation state and protonation state of the proteins, while substantially affecting some parts of the proteins, have little effect on the substrate binding site. Accordingly, our models are not sensitive to protein state, as could be hoped because the substrate site is relatively far (>12 Å) from the T2/T3 copper site.

Our results suggest that laccase substrates require favorable binding at the T1 site in order to achieve a low K_m_, but that additional electronic properties of the substrates should be optimized as they affect K_m_. This suggests that K_m_ contains some elements of a binding affinity of the substrate-enzyme complex, but also additional features relating to the electron transfer, in agreement with an interpretation of Michaelis-Menten kinetic parameters^75^. For laccases, this is particularly clear in the sense that only conformations that actively engage in electron transfer by optimal association with T1 manifest and contribute to observed kinetics, and this explains why K_m_ depend on both affinity but also electronic features of the substrates.

Finally, we tested the predictive capabilities of our recommended models 5 and 6 on a series of additional substrates. The trend prediction was accurate except for a few cases. The results show that the ortho methoxy group in sinapic acid and ferulic acid is the main cause of their low K_m_ as compared to their analog *p*-coumaric acid with high K_m_ and with no ortho methoxy group. This ortho methoxy group forms a hydrogen bond with His-458 of TvL and aids in stabilizing the identified active conformations, which probably accounts for real laccase turnover. Our results also show that a model of laccase turnover should largely account for the active substrate conformations rather than details of the T2/T3 site.

## Electronic supplementary material

Supplementary information

## Data Availability

The data required to reproduce the present computational work are included in the file named “Suppinfo.pdf”. It includes information about the ligand datasets A, B and C, ligand interaction diagrams, details on QSAR models, definitions of the model descriptors, RMSF plots from MD simulations, Ramachandran plot of the CuL model, sequence alignment of laccases, hydrophobicity plots of TvL and CuL, scatter plots of experimental log(relative-activity) and pK_m_ versus ΔG_bind(MMGBSA)_, QSAR descriptor correlation with pK_m_ and log(relative-activity), prediction of the external dataset using QSAR models 5 and 6, and RMSD plots.
