# Supplementary material for: A structural-chemical explanation of fungal laccase activity
Source: Sci Rep. 2018 Nov 23;8:17285. doi: 10.1038/s41598-018-35633-8 (PMC6251875; doi:10.1038/s41598-018-35633-8)
Supplement: Supplementary file 1 — Supplementary information [file 41598_2018_35633_MOESM1_ESM.pdf]

SUPPORTING INFORMATION

A structural-chemical explanation of fungal laccase activity

Rukmankesh Mehra[1,2], Jan Muschiol[2], Anne Meyer[2,*], and Kasper P. Kepp[1,*]

[1] Technical University of Denmark, DTU Chemistry, Building 206, 2800 Kgs. Lyngby, Denmark. [2] Technical University of Denmark, DTU Bioengineering, Building 221, 2800 Kgs. Lyngby, Denmark.

Corresponding Authors

[*] Kasper P. Kepp, E-mail: kpj@kemi.dtu.dk

[*] Anne S. Meyer, E-mail: asme@dtu.dk

ORCID of Authors

0000-0001-6010-1514, 0000-0002-2852-6747, 0000-0001-8910-9931, 0000-0002-6754-7348

Table S1 Dataset A compounds

Compound name	PubChem CID	K_m (µM)	pK_m
Syringaldazine	5379425	8.0	5.10
Sinapic acid	637775	12.1	4.92
OH-Dilignol[a]	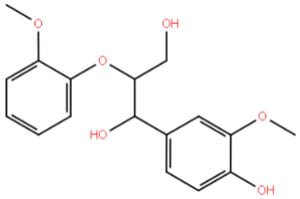	12.9	4.89
Ferulic acid	445858	17.2	4.77
2,6-Dimethoxyphenol	7041	26.0	4.59
ABTS	9570474	38.0	4.42
Syringic acid	10742	130.0	3.89
p-Coumaric acid	637542	130.3	3.89
Vanillic acid	8468	280.0	3.55
Catechol	289	1110.0	2.96
Dopamine	681	2100.0	2.68

[a] Structure of the compound OH-Dilignol was not available in PubChem and therefore, drawn using Maestro.

Table S2 Dataset B compounds

Compound name	PubChem CID	Relative activity	log (activity)
2-6-Dichlorophenol	6899	258	2.41
Hydrocaffeic acid	348154	185	2.27
Catechol	289	176	2.25
2,6-Dimethoxyphenol	7041	159	2.20
o-Chlorophenol	7245	150	2.18
Phenol	996	100	2.00
2-4-Dichlorophenol	8449	93	1.97
Hydroquinone	785	89	1.95
Resorcinol	5054	73	1.86
Guaiacol	460	61	1.79
p-Toluidine	7813	51	1.71
m-Chlorophenol	7933	50	1.70
Caffeic acid	689043	39	1.59
o-Cresol	335	39	1.59
Pyrogallol	1057	10	1.00
p-Hydroxybenzoic acid	135	3	0.48

Table S3 Dataset C compounds

**Compound name**	**Compound code**	**PubChem CID**	**K_m (uM)**	**pK_m**
2,2´-Azino-bis (3-ethylbenzo-thiazoline-6-sulfonic acid)	ABTS	5464076	20	4.70
n-(1-Naphthyl) ethylendiamine	NEDA	15107	120	3.92
3-Amino-4-hydroxybenzenesulfonic acid	AHBS	7385	180	3.75
2-Methoxyhydroquinone	MeHQ	69988	220	3.66
Pyrogallol	Pyr	1057	280	3.55
Epinephrine	Eph	5816	430	3.37
Acetosyringone	ACS	17198	490	3.31
3-(3,4-Dihydroxyphenyl)-L-alanine	DHPAla	6047	540	3.27
4-Methoxyphenol	4MxPh	9015	660	3.18
Norepinephrine	Neph	439260	960	3.02
D-Catechin	CatN	9064	1040	2.98
Catechol	Cat	289	1590	2.80
2-Amino-3-methoxybenzoic acid	AMxBA	255720	1730	2.76
3,4-Dihydroxybenzoic acid	3-4DHB	72	2130	2.67
2-Aminophenol	2Aph	5801	3120	2.51
3-Aminobenzoic acid	MABA	7419	4370	2.36
4-Methylaminobenzoic acid	PMAB	66345	12330	1.91
4-Aminophenol	4Aph	403	13540	1.87
4,5-Dihydroxy-1,3-benzene-disulfonic acid	dHBdSA	1549062	14390	1.84
4-Aminobenzoic acid	PABA	978	21210	1.67
Anthranilamide	AntAmi	6942	34540	1.46
Anthranilic acid	AntAcid	227	234800	0.63
Sodium-1-naphtyl phosphate	SNP	2114	307200	0.51

Table S4 Preparation of binding complexes of laccases

Protein state	Protein	OH^-/ H_2O	T1 Cu state	T3 Cu α state	T3 Cu β state	T2 Cu state
Resting oxidized (RO)	TvL	$2OH^-$, $4H_2O$	+2	+2	+2	+2
	CuL	$2OH^-$, $4H_2O$	+2	+2	+2	+2
$3e^-$ reduced	TvL	$6H_2O$	+2	+1	+1	+1
	CuL	$6H_2O$	+2	+1	+1	+1

Table S5 Charge on protein complexes at different pH

**Protein state**	**Protein**	**Protein charge at pH 7.0**	**Protein charge at pH 4.5**	**Protein charge at pH 4.5 Ash-206**
Resting oxidized (RO)	TvL	-3	+3	+4
	CuL	-22	-18	-17
3e- reduced	TvL	-4	+2	+3
	CuL	-23	-19	-18

Table S6 Number of atoms, counterions, water molecules and volume of each solvated protein system of MD simulation

		**Protein**			
		**TvL**		**CuL**	
		**RO**	**$3e^-$ reduced**	**RO**	**$3e^-$ reduced**
pH 7.0	Atoms	47690	47675	47099	47081
	Counterions	3 Na^+	4 Na^+	22 Na^+	23 Na^+
	Water molecules	13405	13401	13245	13240
	Box Volume ($Å^3$)	494214	494253	494151	494185
pH 4.5	Atoms	47750	47766	47168	47174
	Counterions	3 Cl^-	2 Cl^-	18 Na^+	19 Na^+
	Water molecules	13423	13430	13268	13271
	Box Volume ($Å^3$)	494097	494083	494088	494130
pH 4.5 Ash-206	Atoms	47728	47747	47189	47180
	Counterions	4 Cl^-	3 Cl^-	17 Na^+	18 Na^+
	Water molecules	13415	13423	13275	13273
	Box Volume ($Å^3$)	494097	494083	494088	494130

Table S7 Ligand interaction diagram of dataset A compounds

<table>
<tr><th>Ligand interaction diagram</th><th>Ligand interaction diagram</th></tr>
<tr><td>
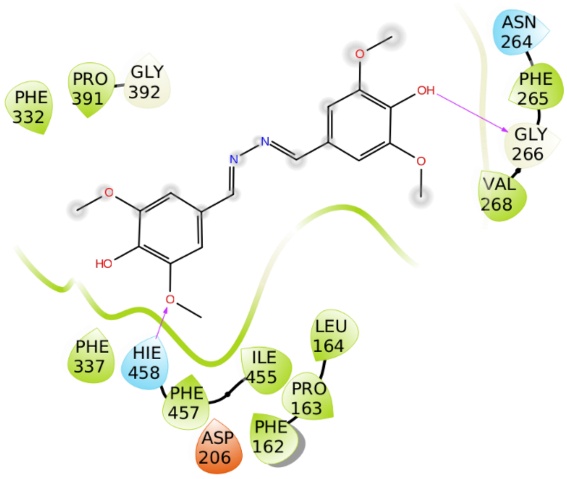

Syringaldazine
</td><td>
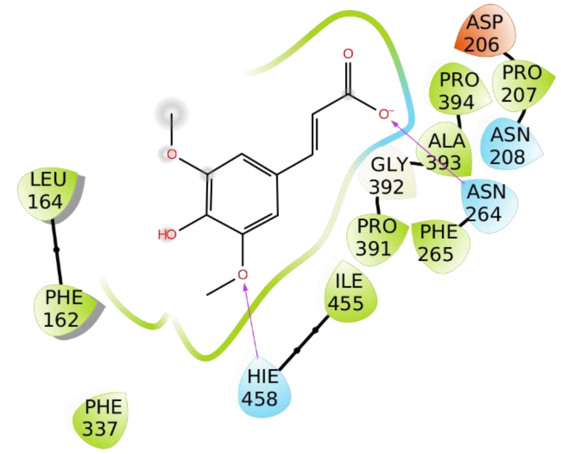

Sinapic acid
</td></tr>
<tr><td>
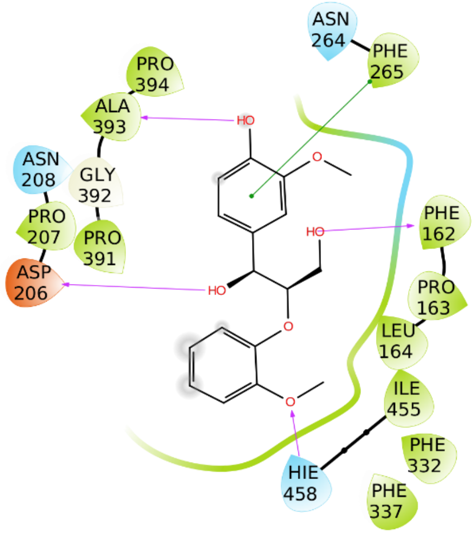

OH-Dilignol
</td><td>
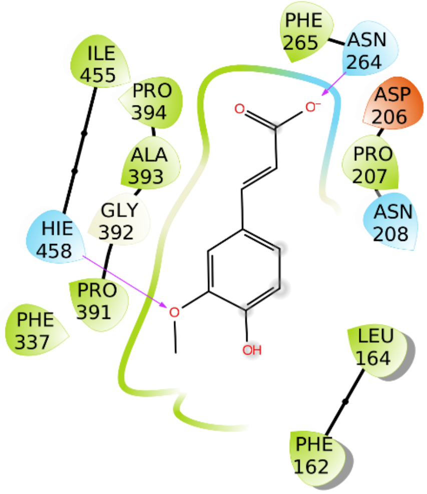

Ferulic acid
</td></tr>
</table>

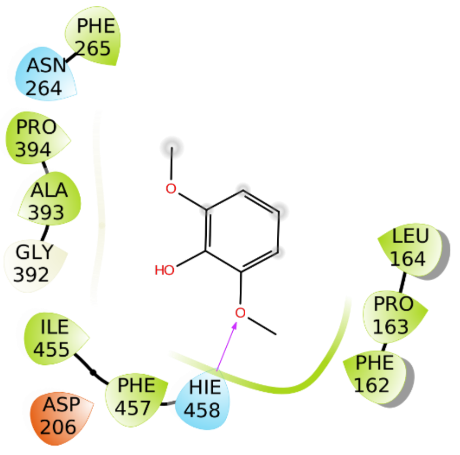

2,6-Dimethoxyphenol

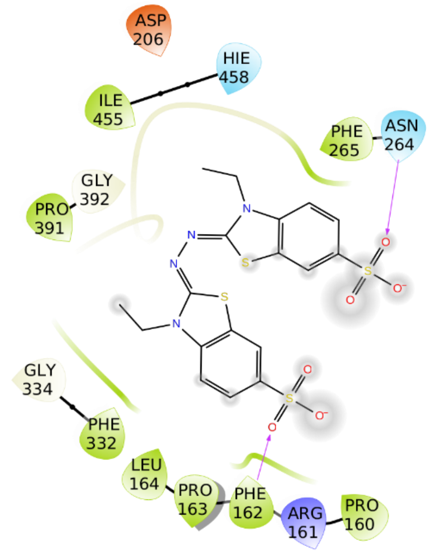

ABTS

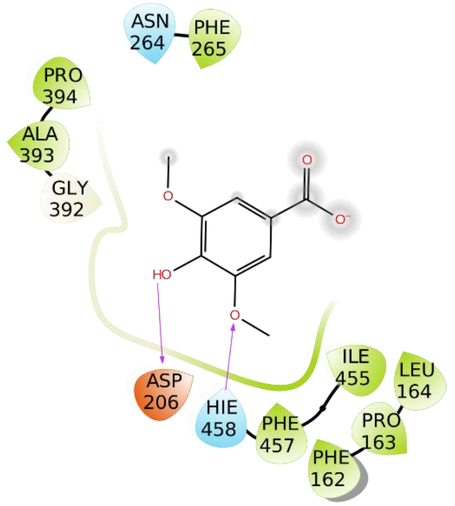

Syringic acid

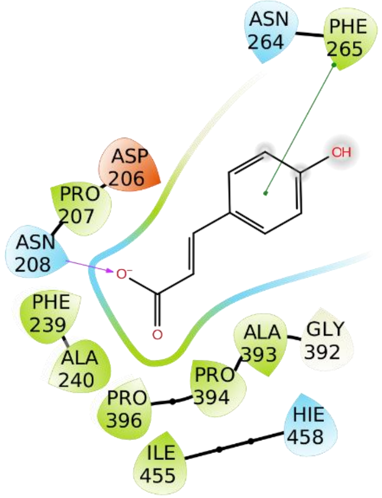

p-Coumaric acid

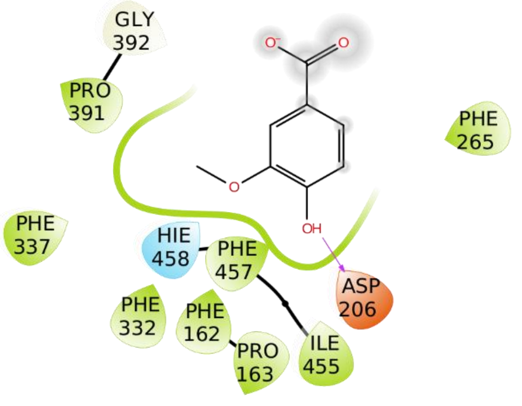

Vanillic acid

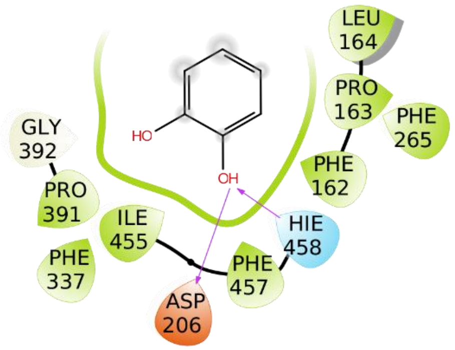

Catechol

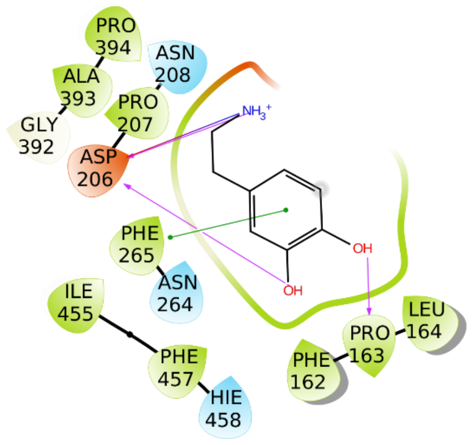PRO 394<br>ASN 208<br>ALA 393<br>PRO 207<br>GLY 392<br>ASP 206<br>NH3+<br>PHE 265<br>ASN 264<br>OH<br>OH<br>ILE 455<br>PHE 457<br>HIE 458<br>LEU 164<br>PRO 163<br>PHE 162<br>Dopamine	

Table S8 Ligand interaction diagram of dataset B compounds

**Ligand interaction diagram**	**Ligand interaction diagram**
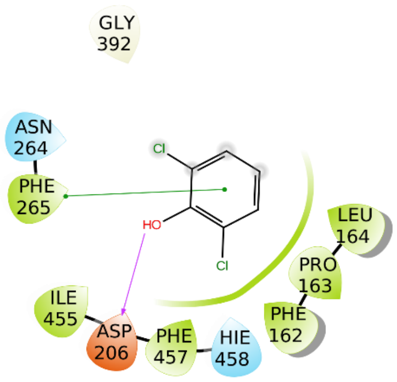<br>2,6-Dichlorophenol	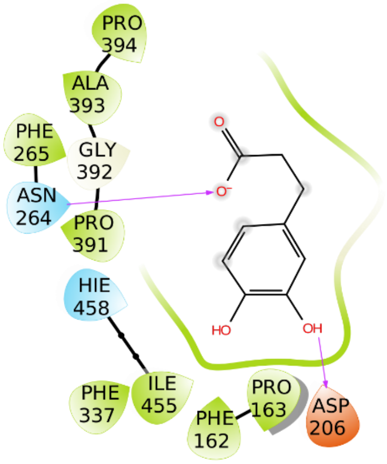<br>Hydrocaffeic acid
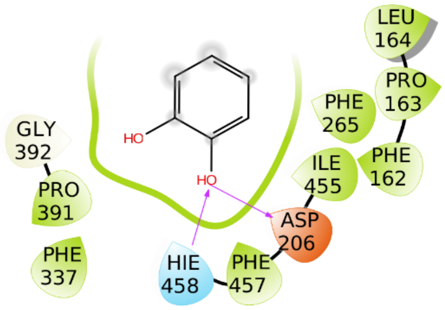<br>Catechol	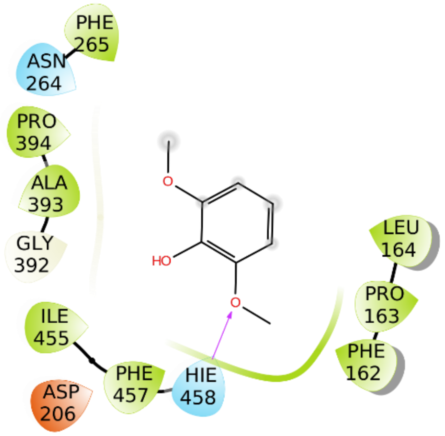<br>2,6-Dimethoxyphenol
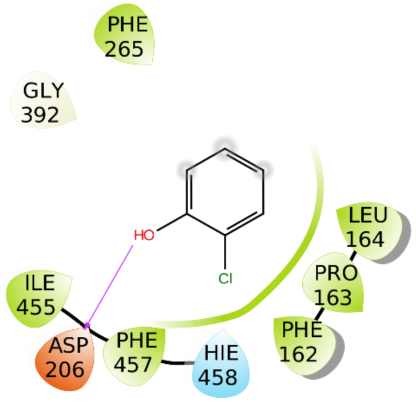<br>*o*-Chlorophenol	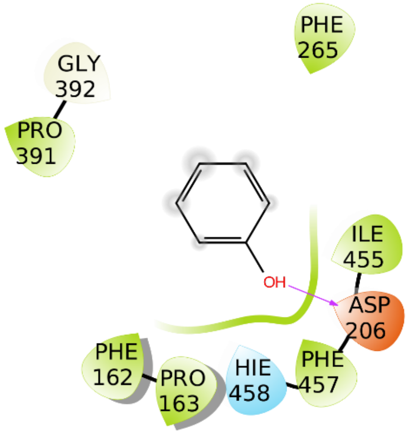<br>Phenol

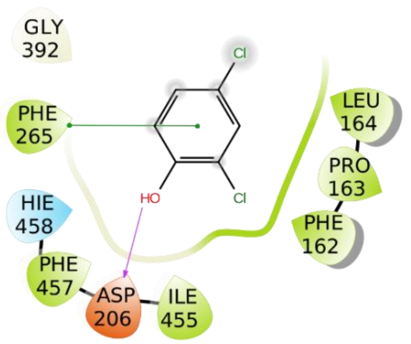

2-4-Dichlorophenol

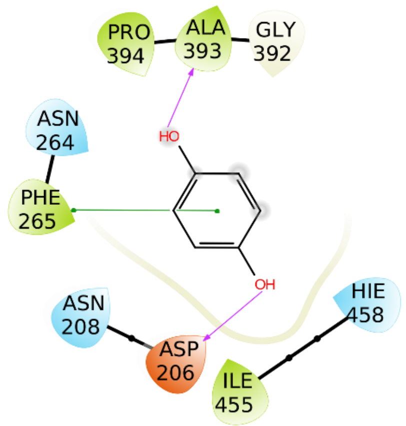

Hydroquinone

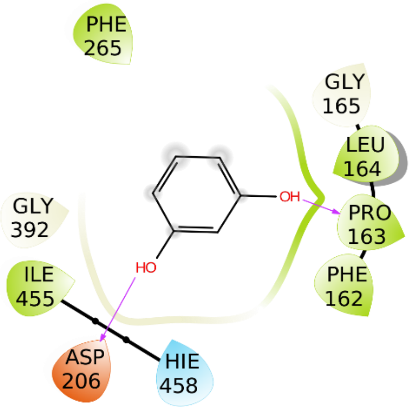

Resorcinol

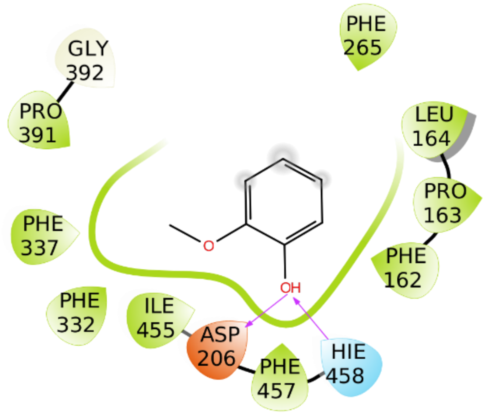

Guaiacol

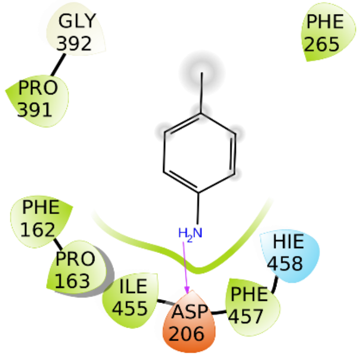

p-Toluidine

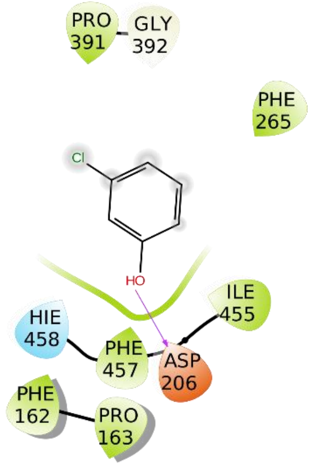

m-Chlorophenol

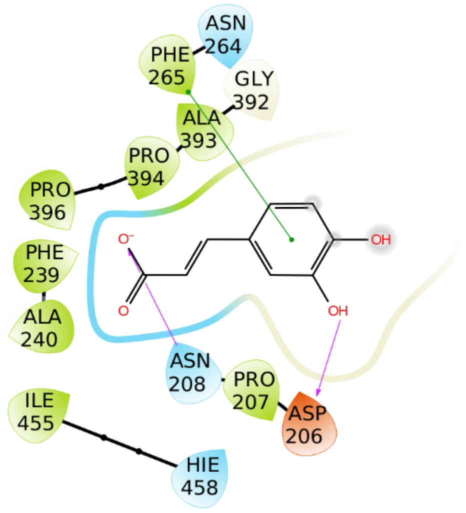

Caffeic acid

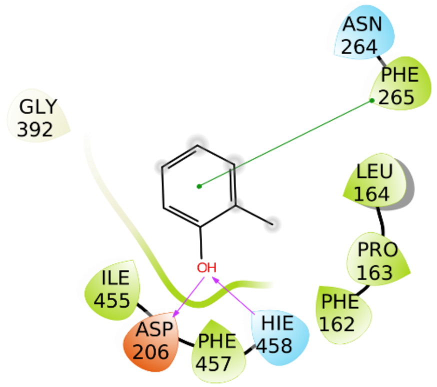

o-Cresol

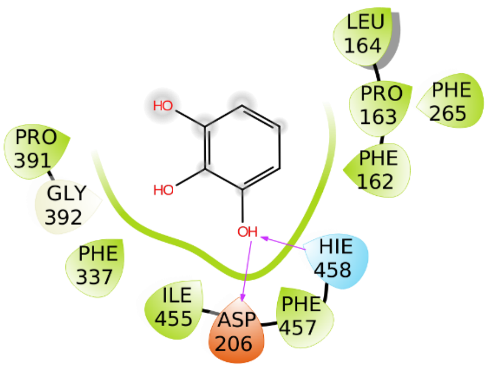

Pyrogallol

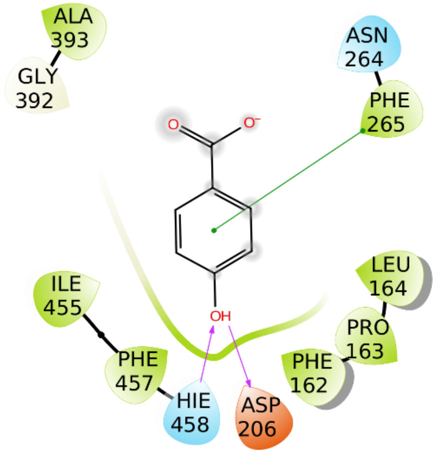

p-Hydroxybenzoic acid

Table S9 Ligand interaction diagram of dataset C compounds

<table>
<tr><th>Ligand interaction diagram</th><th>Ligand interaction diagram</th></tr>
<tr><td>
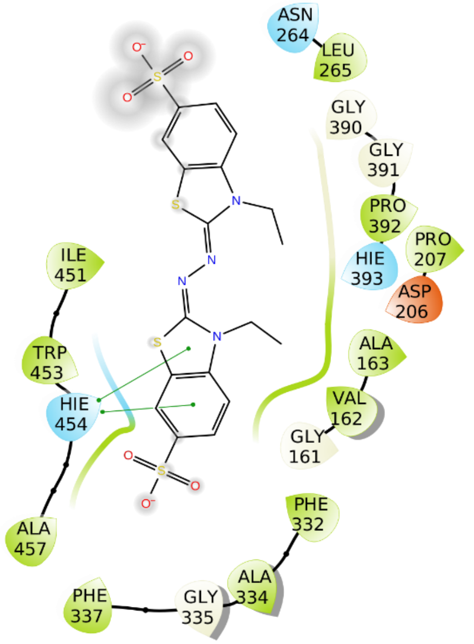

ABTS
</td><td>
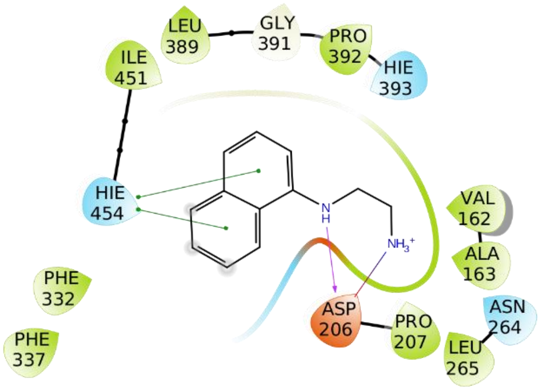

NEDA
</td></tr>
<tr><td>
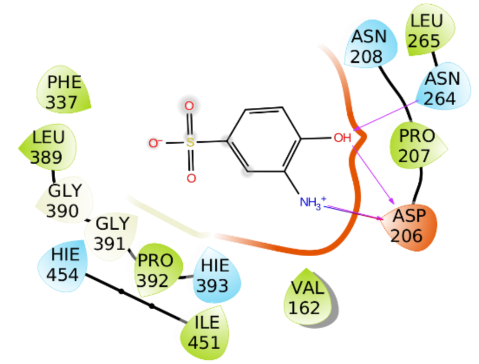

AHBS
</td><td>
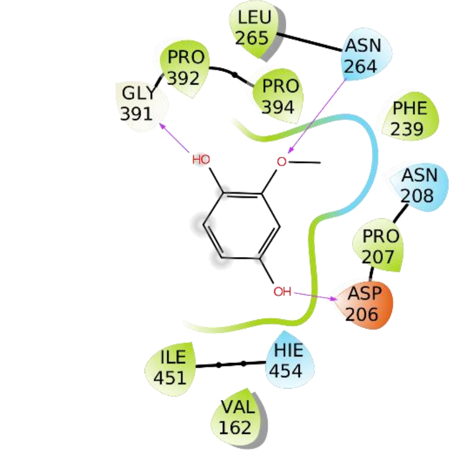

MeHQ
</td></tr>
<tr><td>
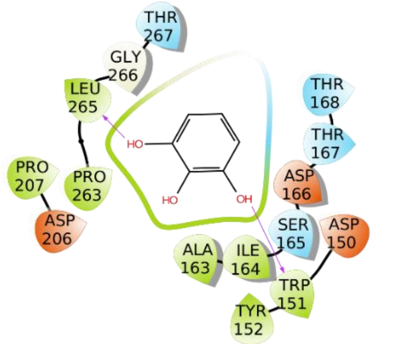

Pyr
</td><td>
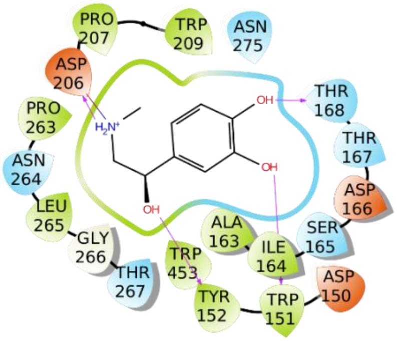

Eph
</td></tr>
</table>

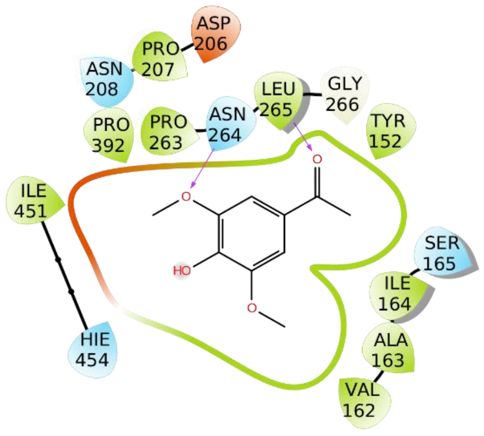

ACS

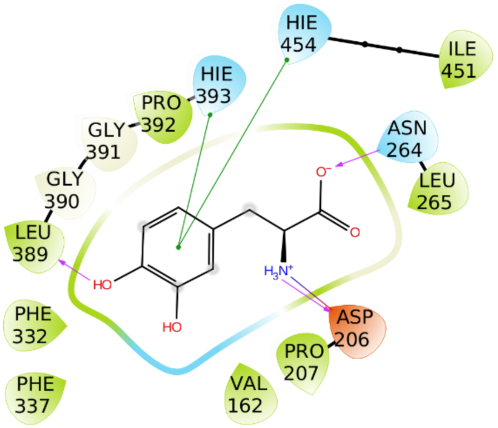

DHPAla

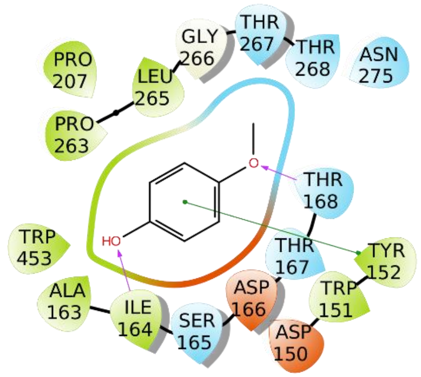

4MxPh

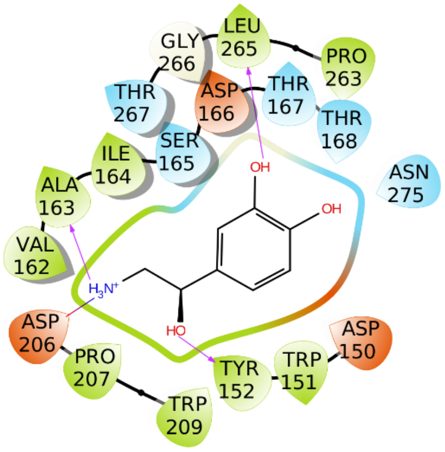

Neph

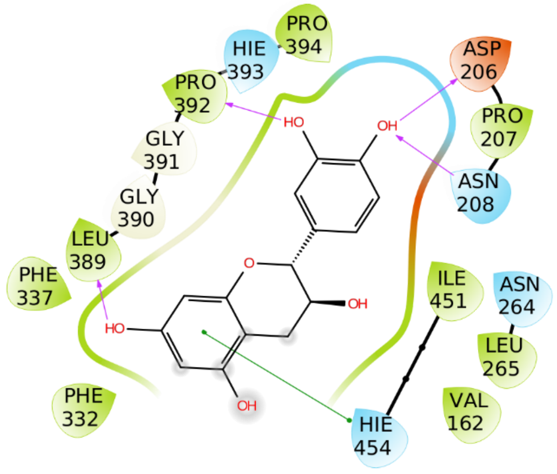

CatN

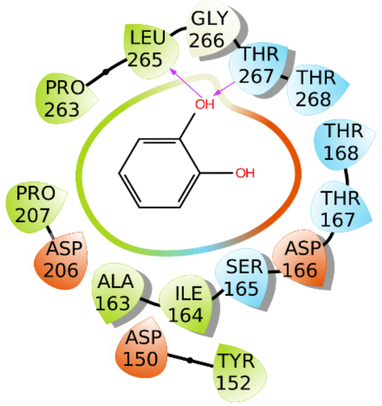

Cat

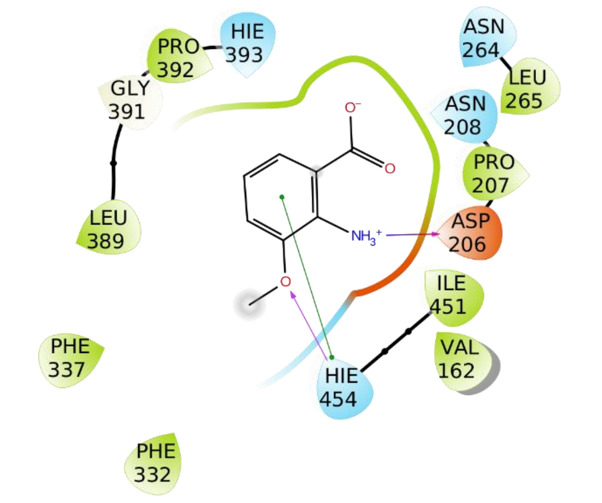

AMxBA

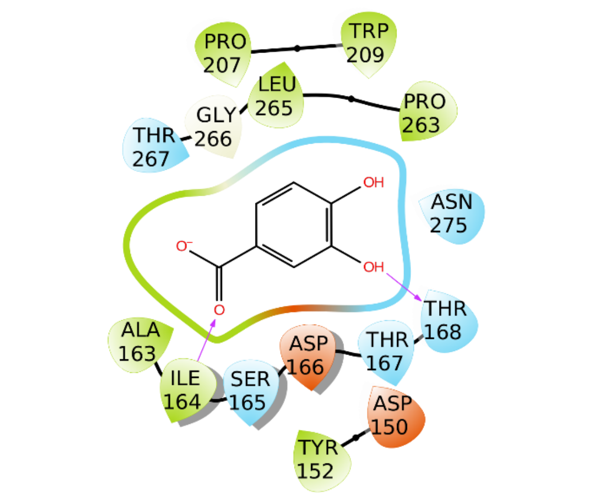

3-4DHB

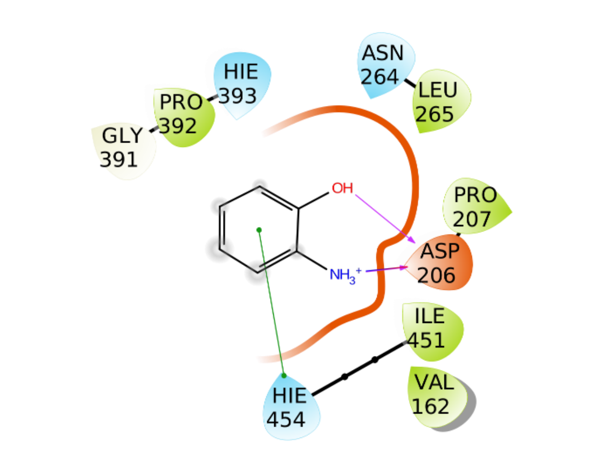

2Aph

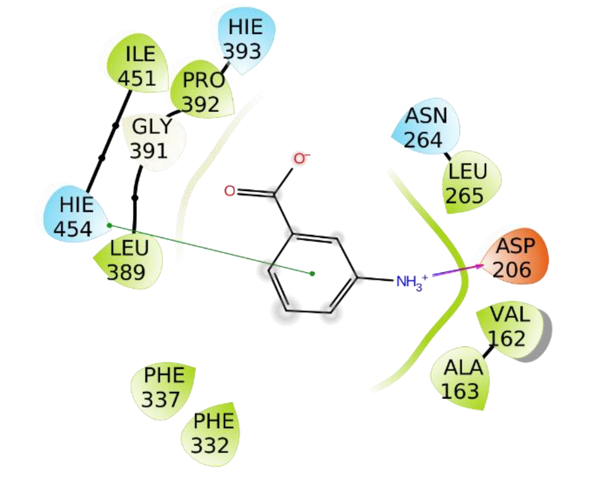

MABA

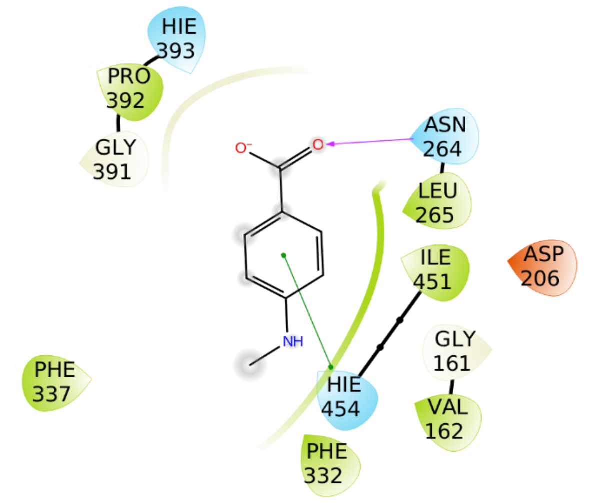

PMAB

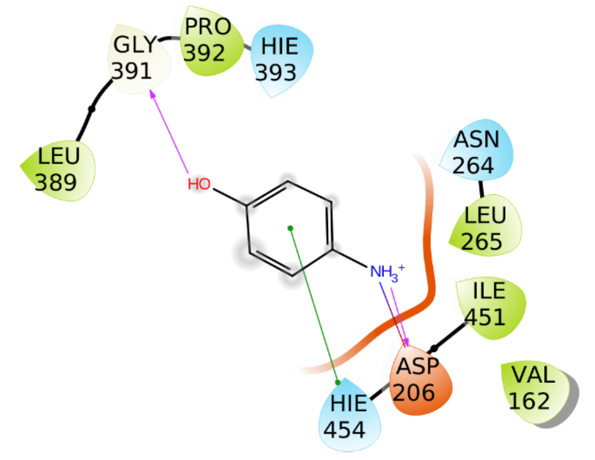

4Aph

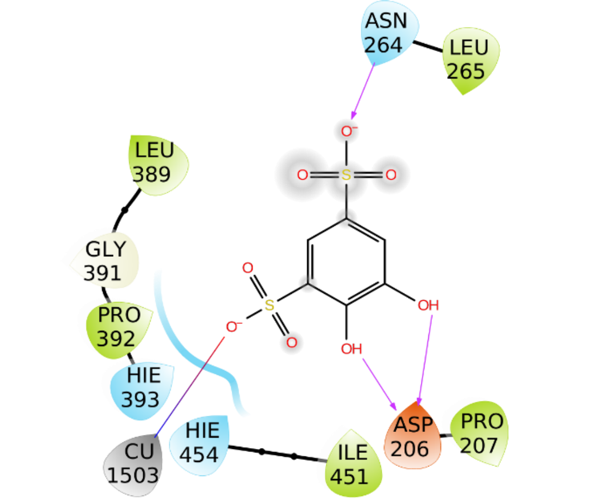

dHBdSA

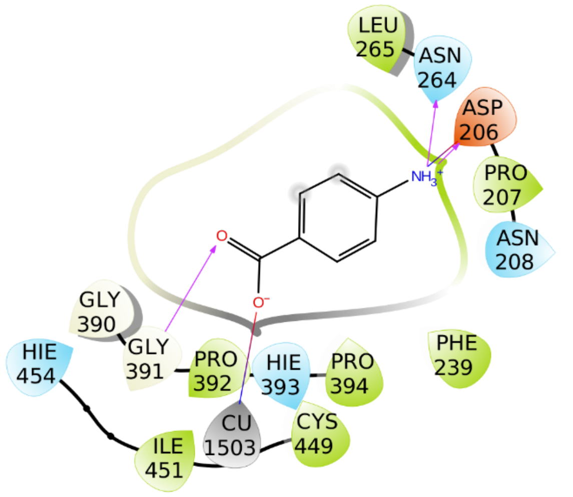

PABA

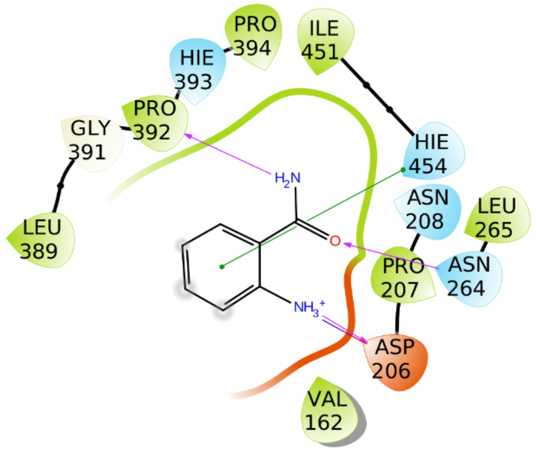

AntAmi

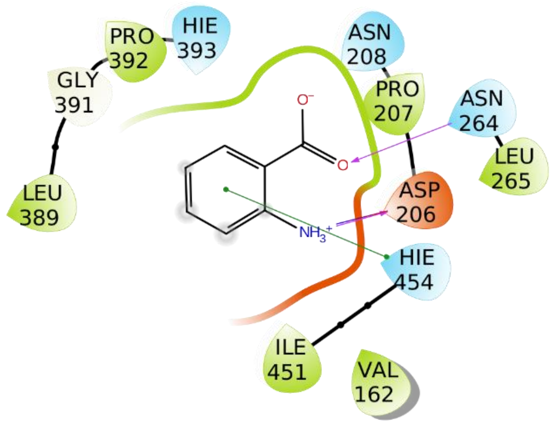

AntAcid

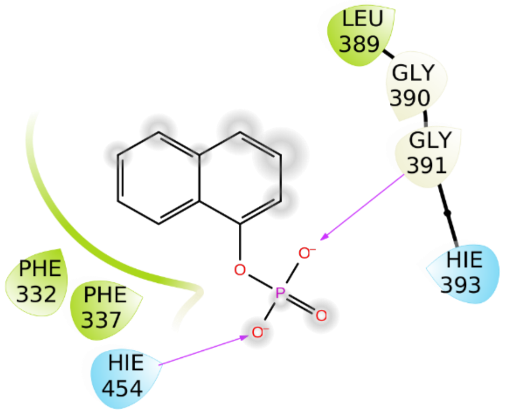

SNP

Table S10 QSAR models

S.No.	Descriptor type	Length	Equation (Activity/pK_m =)	R^2	Q^2
Dataset B / Free ligands (n = 12[a] for Global, QM and SE models; n = 15[b] for ADMET models)					
1	Global	2	4.12e+00<br>-1.73e+01 LUMO<br>-4.36e-02 ESP max	0.65	0.39
2	Global	3	3.32e+00<br>+ 4.96e-01 QPlogPo/w<br>+ 3.95e-01 QPlogS<br>-3.91e-02 ESP max	0.76	0.57
3	ADMET	2	2.62e+01<br>-2.26e+01 dip^2/V<br>-2.55e+01 glob	0.40	0.03
4	ADMET	3	-2.28e+01<br>+ 6.51e-01 QPlogPo/w<br>+ 9.25e-01 QPlogS<br>+ 1.62e+01 Vol/SASA	0.79	0.62
5	ADMET	3	4.65e+01<br>-1.09e-02 FISA<br>-4.64e+01 glob<br>+ 6.31e-01 QPlogS	0.74	0.50
6	ADMET	3	4.84e+01<br>-4.84e+01 glob<br>+ 5.81e-01 QPlogS<br>-2.60e-02 PSA	0.74	0.47
7	ADMET	3	4.12e+01<br>-1.23e+02 ACxDN^.5/SA<br>-4.10e+01 glob<br>+ 5.38e-01 QPlogS	0.704	0.41
8	QM	2	4.12e+00<br>-1.73e+01 LUMO<br>-4.36e-02 ESP max	0.65	0.39
9	QM	2	2.30e+00<br>-3.90e-02 ESP max	0.47	0.18

			+ 1.43e+01 Electronegativity (IP + EA)/2		
10	QM	2	-7.13e-01<br>-4.27e-02 ESP max<br>+ 1.64e+01 IP Total internal energy difference (au)	0.47	0.21
11	QM	3	3.94e+00<br>-4.89e-02 ESP max<br>+ 2.30e+01 Gap (HOMO-LUMO)<br>+ 1.84e+01 IP Total internal energy difference (au)	0.69	0.32
12	SE	2	8.18e+00<br>-1.67e+00 Max ESP On Mol Surface<br>+ 3.83e-02 Min ALEA On Mol Surface	0.61	0.38
13	SE	3	1.29e+01<br>+ 7.52e-01 Total Electrophilic Superdelocalizability<br>-2.64e+00 Max ESP On Mol Surface<br>+ 5.43e-02 Min ALEA On Mol Surface	0.83	0.49
Dataset B / Bound ligands (n = 12[a] for Global, QM and SE models; n = 15[b] for ADMET models)					
14	Global	3	3.44e+00<br>+ 9.70e-03 PISA<br>+ 1.70e+00 EA(eV)<br>-6.05e-02 ESP max	0.80	0.59
15	Global	3	3.57e+01<br>+ 2.44e-02 mol MW<br>-2.06e+01 Vol/SASA<br>-1.22e-01 ESP max	0.82	0.50
16	Global	3	2.55e+01<br>-7.87e-01 CIQPlogS<br>-1.33e+01 Vol/SASA<br>-9.93e-02 ESP max	0.80	0.63
17	Global	3	1.95e+01<br>+ 1.54e+00 EA(eV)<br>-8.65e+00 Vol/SASA<br>-9.04e-02 ESP max	0.79	0.51
18	Global	3	3.50e+00	0.70	0.50

			+ 4.42e-01 QPlogPo/w<br>+ 3.60e-01 QPlogS<br>-4.18e-02 ESP max		
19	Global	3	1.03e+01<br>-1.43e-02 SASA<br>+ 1.67e+00 EA(eV)<br>-7.80e-02 ESP max	0.76	0.41
20	Global	2	9.43e+00<br>-1.06e-01 ESP max<br>-3.05e-02 Zero Point Energy (kcal/mol)	0.83	0.64
21	Global	3	2.88e+00<br>+ 8.94e-01 QPlogS<br>-1.34e-02 ESP pos variance (kcal/mol)**2<br>-1.89e-03 Total Free Energy at 298.15K (au)	0.92	0.80
22	Global	3	2.37e+01<br>-1.14e+01 Vol/SASA<br>-1.08e-01 ESP max<br>-1.12e-03 Total Free Energy at 298.15K (au)	0.83	0.63
23	Global	3	2.37e+01<br>-1.14e+01 Vol/SASA<br>-1.08e-01 ESP max<br>-1.12e-03 Total Enthalpy at 298.15K (au)	0.83	0.63
24	ADMET	2	1.25e+00<br>+ 7.70e-03 mol MW<br>-1.79e+01 dip^2/V	0.51	0.09
25	ADMET	3	-2.69e+00<br>+ 8.65e-03 volume<br>+ 6.56e-01 QPlogPo/w<br>+ 8.31e-01 QPlogS	0.81	0.65
26	ADMET<br>(same as found for ligprep poses)	3	-1.72e+01<br>+ 6.27e-01 QPlogPo/w<br>+ 8.36e-01 QPlogS<br>+ 1.25e+01 Vol/SASA	0.82	0.67
27	QM	2	same as Global		

28	QM	2	2.66e+00<br>-5.27e-02 ESP max<br>+ 1.64e+01 Electronegativity (IP + EA)/2	0.58	0.23
29	QM	2	1.18e-02<br>-4.87e-02 ESP max<br>+ 1.47e+01 IP Total free energy difference (au)	0.55	0.21
30	QM	3	5.07e+00<br>-6.07e-02 ESP max<br>+ 3.05e+01 Gap (HOMO-LUMO)<br>+ 2.20e+01 IP Total internal energy difference (au)	0.74	0.48
31	QM	3	4.78e+00<br>-5.88e-02 ESP max<br>+ 2.92e+01 Gap (HOMO-LUMO)<br>+ 2.18e+01 IP Total free energy difference (au)	0.74	0.46
32	QM	3	8.86e+00<br>-2.98e-02 MMGBSA dG bind 0A pose specific score<br>-1.08e-01 ESP max<br>-3.10e-02 Zero Point Energy (kcal/mol)	0.88	0.68
33	SE	2	6.60e+00<br>-1.74e+00 Max ESP On Mol Surface<br>+ 2.47e-02 Min ALEA On Mol Surface	0.55	0.34
34	SE	3	5.90e+00<br>-1.63e-01 Dipole ESP Y<br>-9.97e+00 Pos ESP Variance On Mol Surface<br>+ 2.91e-02 Min ALEA On Mol Surface	0.67	0.41
Dataset C / Free ligands (n = 22)[c]					
35	Global	1	1.39e+01<br>-1.21e+00 IP(eV)	0.62	0.53
36	Global	2	1.27e+01<br>-1.12e+00 IP(eV)<br>-1.41e-02 MMGBSA dG bind 8A top score	0.64	0.54
37	Global	2	1.39e+01<br>-1.22e+00 IP(eV)	0.62	0.51

			-2.68e-04 $E_{Solv(PBF)}$		
38	Global	3	1.22e+01<br>+ 2.34e-01 accptHB<br>-1.07e+00 IP(eV)<br>+ 8.13e-03 $E_{Solv(PBF)}$	0.77	0.66
39	Global	3	2.01e+01<br>-1.78e+01 glob<br>-3.46e-02 MMGBSA dG bind 8A top score<br>-2.04e-03 Eo(expt)	0.64	0.45
40	ADMET	2	1.42e+01<br>+ 3.40e+01 ACxDN^.5/SA<br>-1.31e+00 IP(eV)	0.70	0.58
41	ADMET	2	2.24e+01<br>-2.12e+01 glob<br>-1.08e+00 EA(eV)	0.54	0.38
42	ADMET	3	1.69e+01<br>-6.64e+00 glob<br>-9.21e-01 IP(eV)<br>-1.6237e-02 MMGBSA dG bind 8A top score	0.68	0.57
43	QM		-	-	-
44	SE		-	-	-
Dataset C / Bound ligands (n=21) [d]					
45	Global	2	-1.03e+01<br>-2.85e-03 Eo(expt)<br>+ 1.00e+01 Vol/SASA	0.67	0.54
46	Global	3	-1.25e+01<br>-3.89e-03 FISA<br>-2.84e-03 Eo(expt)<br>+ 1.18e+01 Vol/SASA	0.74	0.60
47	Global	3	-9.46e+00<br>-1.85e-02 MMGBSA dG bind pose specific score<br>-2.63e-03 Eo(expt)<br>+ 9.12e+00 Vol/SASA	0.69	0.54
48	ADMET	2	same as Global		

49	ADMET	3	same as Global		
50	ADMET	3	same as Global		
51	ADMET	3	2.97e+00<br>+ 1.90e-02 mol MW<br>-7.01e-01 QPlogPo/w<br>-4.20e-02 PSA	0.65	0.53
52	QM		-	-	-
53	SE		-	-	-

[a] Outlier compound Toluidine and three anions in the dataset were removed; [b] Outlier compound Toluidine was removed; [c] Outlier compound SNP was removed for calculations; [d] Outlier compounds SNP and Pyr were removed for calculations.

Table S11 Description of the descriptors found in the QSAR models

Descriptor	Description
**QikProp descriptors**	
QPlogPo/w	Predicted octanol/water partition coefficient.
QPlogS	Predicted aqueous solubility, log S. S in mol dm–3 is the concentration of the solute in a saturated solution that is in equilibrium with the crystalline solid.
dip^2/V	Square of the dipole moment divided by the molecular volume. This is the key term in the Kirkwood-Onsager equation for the free energy of solvation of a dipole with volume V.
glob	Globularity descriptor, $(4\pi r^2)$/(SASA), where *r* is the radius of a sphere with a volume equal to the molecular volume. Globularity is 1.0 for a spherical molecule.
FISA	Hydrophilic component of the SASA (SASA on N, O, H on heteroatoms, and carbonyl C).
PSA	Van der Waals surface area of polar nitrogen and oxygen atoms and carbonyl carbon atoms.
ACxDN^.5/SA	Index of cohesive interaction in solids.
PISA	π-(carbon and attached hydrogen) component of the SASA.
EA(eV)	PM3 calculated electron affinity (negative of LUMO energy).
mol MW	Molecular weight of the molecule.
CIQPlogS	Conformation-independent predicted aqueous solubility, log S. S in mol dm^{-3} is the concentration of the solute in a saturated solution that is in equilibrium with the crystalline solid.
SASA	Total solvent accessible surface area (SASA) in square angstroms using a probe with a 1.4 Å radius.
Volume	Total solvent-accessible volume in cubic angstroms using a probe with a 1.4 Å radius.
IP(eV)	PM3 calculated ionization potential (negative of HOMO energy).
accptHB	Estimated number of hydrogen bonds that would be accepted by the solute from water molecules in an aqueous solution. Values are averages taken over a number of configurations, so they can be non-integer.
**Jaguar descriptors**	
LUMO	Lowest unoccupied molecular orbital energy.
ESP max	Maximum of the electrostatic potential energy in kilocalorie per mole. The ESP (electrostatic potential) describes the potential energy experienced by a positive test charge at a point in space.
Zero Point Energy (kcal/mol)	Vibrational energy at 0 K calculated for a Harmonic

	Oscillator. It is the sum of the vibrational ground state energies for each normal mode in kilocalorie per mole.
ESP pos variance (kcal/mol)**2	Variance of positive contributions to Electrostatic Potential in kilocalorie per mole
Total Free Energy at 298.15K (au)	Total Gibbs free energy (G_{tot}) at 298.15 K (in atomic unit), including the SCF energy and zero point energy.
Total Enthalpy at 298.15K (au)	Total enthalpy (H_{tot}) at 298.15K (in atomic unit), including the SCF energy and zero point energy.
$E_{Solv(PBF)}$	Solvation Energy calculated using water solvent by Poisson–Boltzmann Finite (PBF) in kilocalorie per mole.
**NDDO semi-empirical descriptors**	
Max ESP On Mol Surface	Maximum electrostatic potential on molecular surface in kilocalorie per mole.
Min ALEA On Mol Surface	Minimum average local electron affinity on molecular surface.
Total Electrophilic Superdelocalizability	Total electrophilic superdelocalizability is the sum over all the atoms of the electrophilic superdelocalizability.
Dipole ESP Y	ESP Dipole moment in Y axis
Pos ESP Variance On Mol Surface	Positive variance of electrostatic potential on molecular surface.
**Manually calculated**	
Vol/SASA	Calculated using Qikprop descriptors as volume divided by the solvent accessible surface area.
Electronegativity (IP + EA)/2	Electronegativity calculated using Jaguar properties. It is sum calculated by the formula (Ionization potential + Electron affinity)/2
IP Total internal energy difference (au)	Ionization potential calculated by the difference in the total internal energies of cationic and neutral compounds. The energies were calculated using Jaguar.
IP Total free energy difference (au)	Ionization potential calculated by the difference in the total free energies of cationic and neutral compounds. The energies were calculated using Jaguar.
Gap (HOMO-LUMO)	Gap between HOMO and LUMO energy
MMGBSA dG bind 0A pose specific score	dG bind energy of the specific ligand poses selected for QSAR analysis calculated by MMGBSA at 0 Å flexibility.
MMGBSA dG bind 8A top score	Highest dG bind energy of a ligand calculated by MMGBSA at 8 Å flexibility
MMGBSA dG bind pose specific score	dG bind energy of the specific ligand poses selected for QSAR analysis calculated by MMGBSA at 8 Å flexibility.
**From literature**	
Eo(expt)	Oxidation peak reported in literature for the dataset C compounds in the original article for this dataset.

Table S12 Prediction of the in-house evaluated substrates using pK_m prediction models of dataset C

**Compounds**	**Experimental pK_m**	**Predicted pK_m**					
		**Free ligand Global[5]**	**Free ligand Global[6]**	**Free ligand Global[36]**	**Free ligand ADMET[40]**	**Free ligand ADMET[42]**	**Bound ligand ADMET [51]**
p-coumaric acid	3.9	2.9	2.6	2.8	2.7	2.8	2
Ferulic acid	4.8	3.2	3.0	3.1	3.1	3.1	2.3
Sinapic acid	4.9	3.3	3.2	3.0	3.2	3.3	2.5
OH-diginol	4.9	3.3-3.4	4.2-4.3	3.8	3.5	3.9-4.4	4

Superscripts [5] and [6] here represent the QSAR models 5 and 6 of the main article used for the predictions. Superscripts [36, 40, 42] and [51] represent the predictions using QSAR models at S. no. 36, 40, 42 and 51 respectively, in the **Supplementary Table S10**. For the other pK_m models in **Supplementary Table 10**, either the Q^2 values were lower than these models or the experimental oxidation peak was used as a descriptor, as not available for these compounds. For the free ligand models, the LigPrep-generated conformations were used. For the bound-ligand QSAR model, the MMGBSA-generated bound conformations were applied.

Table S13 RMSF plots of Cα for the last 20 ns of the MD run. In plot, blue color represents β strand, red color shows α helix and white color indicate the loop region for more than 70% of last 20 ns simulation time. B factors of the residues are also shown with the scale to the right.

MD complex	RMSF plot
TvL/ RO/ pH 7.0	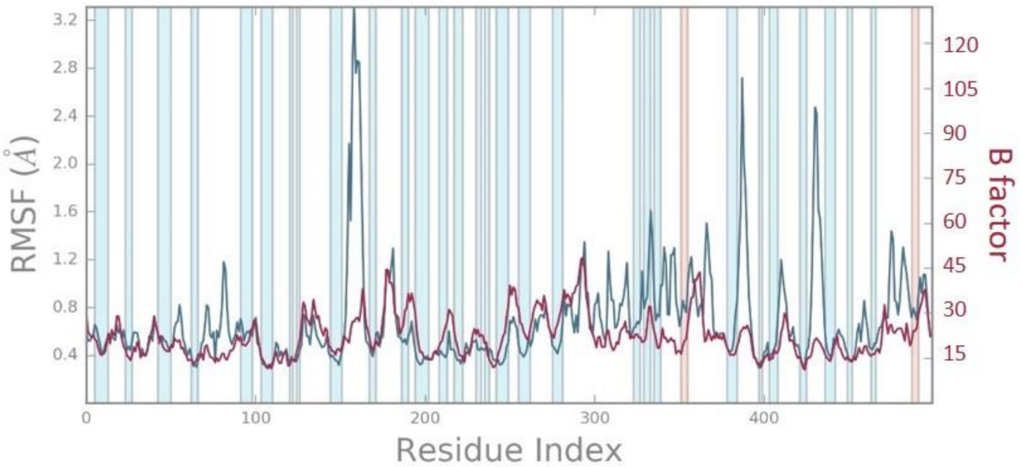
TvL/ RO/ pH 4.5	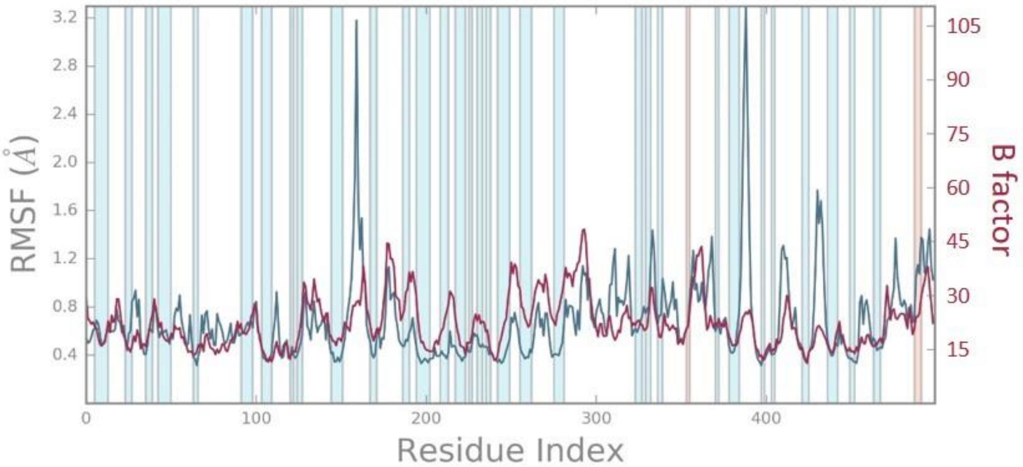

<table>
<tr><td>TvL/ RO/ pH 4.5 Ash 206</td><td>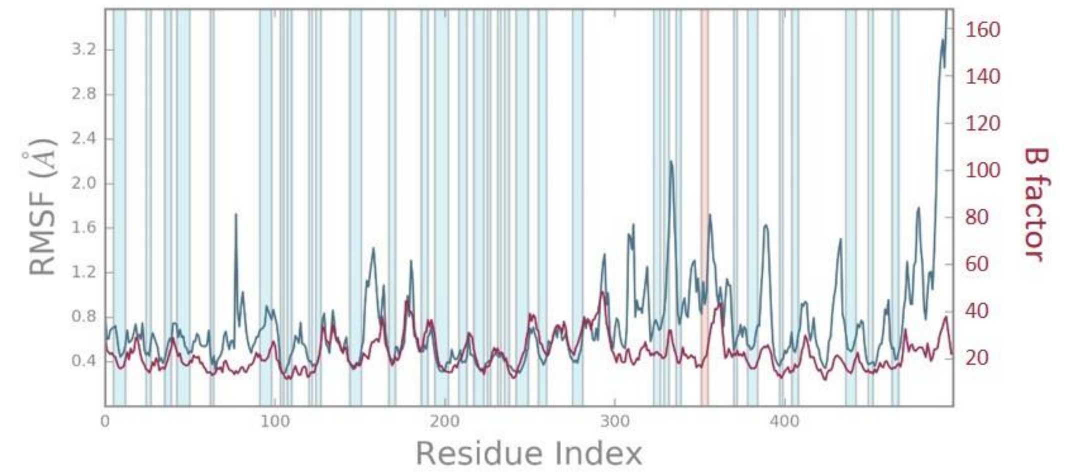
</td></tr>
<tr><td>TvL/ $3e^{-}$ reduced/ pH 7.0</td><td>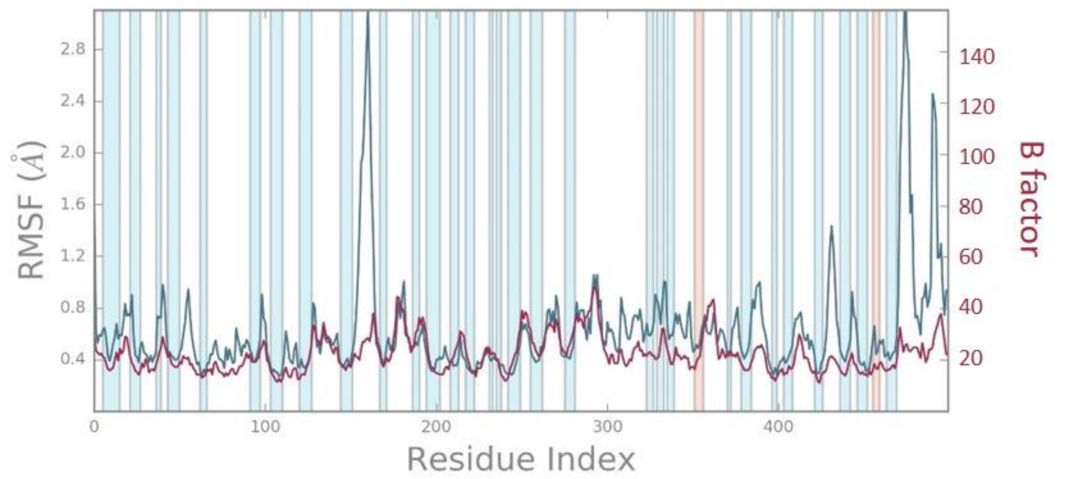
</td></tr>
<tr><td>TvL/ $3e^{-}$ reduced/ pH 4.5</td><td>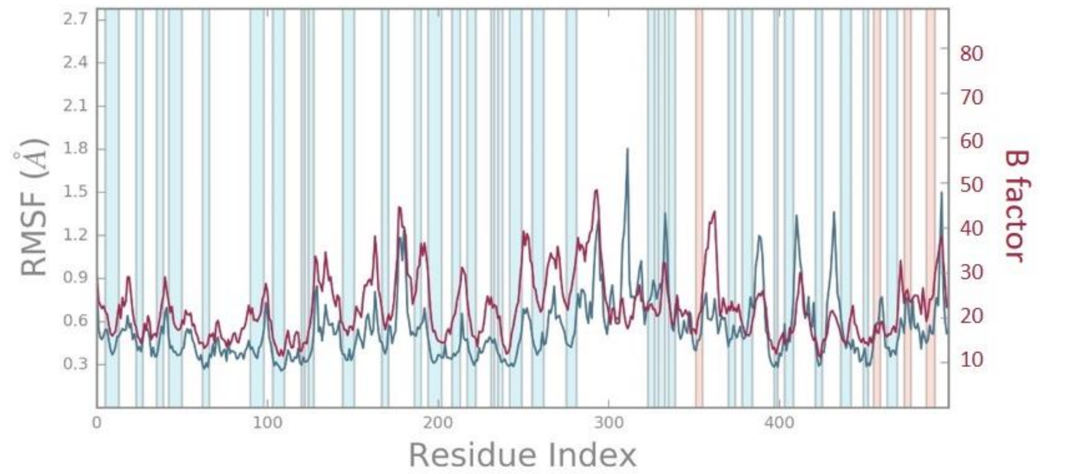
</td></tr>
</table>

<table>
<tr>
<td>TvL/ 3e$^-$ reduced/ pH 4.5 Ash206</td>
<td>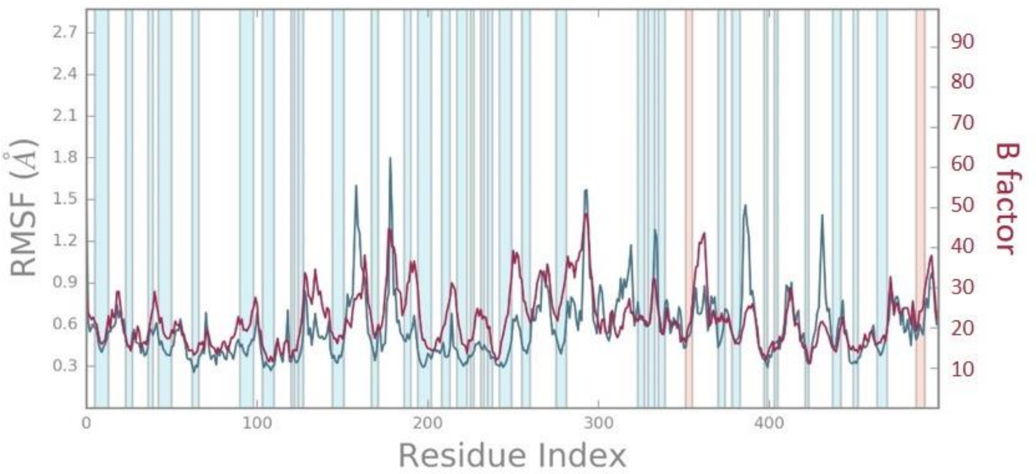
</td>
</tr>
<tr>
<td>CuL/ RO/ pH 7.0</td>
<td>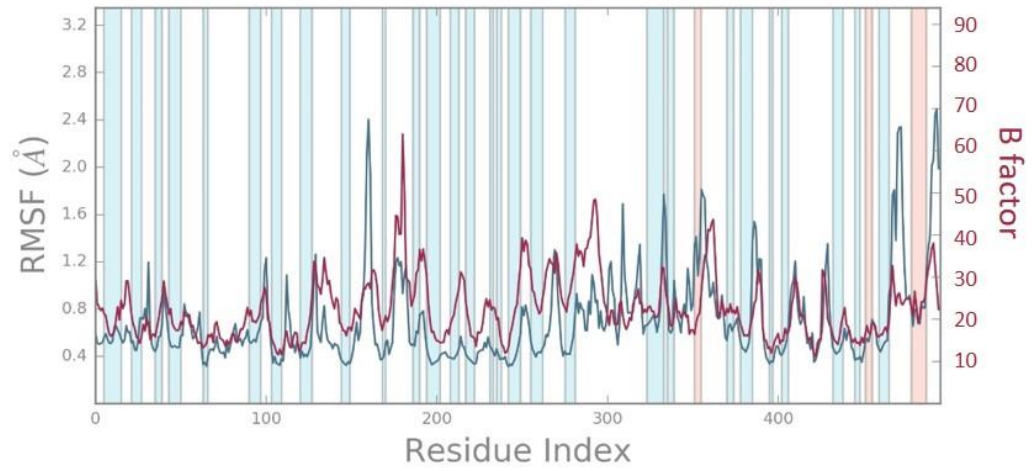
</td>
</tr>
<tr>
<td>CuL/ RO/ pH 4.5</td>
<td>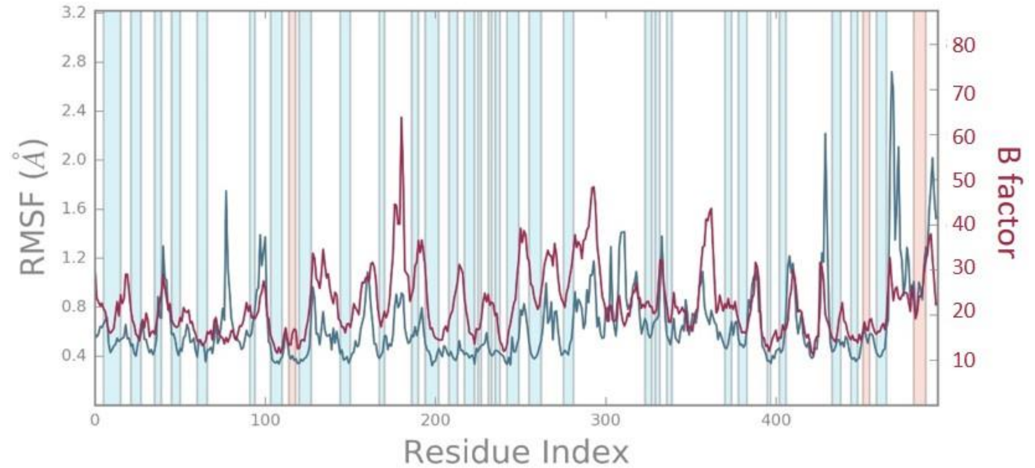
</td>
</tr>
</table>

<table>
<tr>
<td>CuL/ RO/ pH 4.5 Ash 206</td>
<td>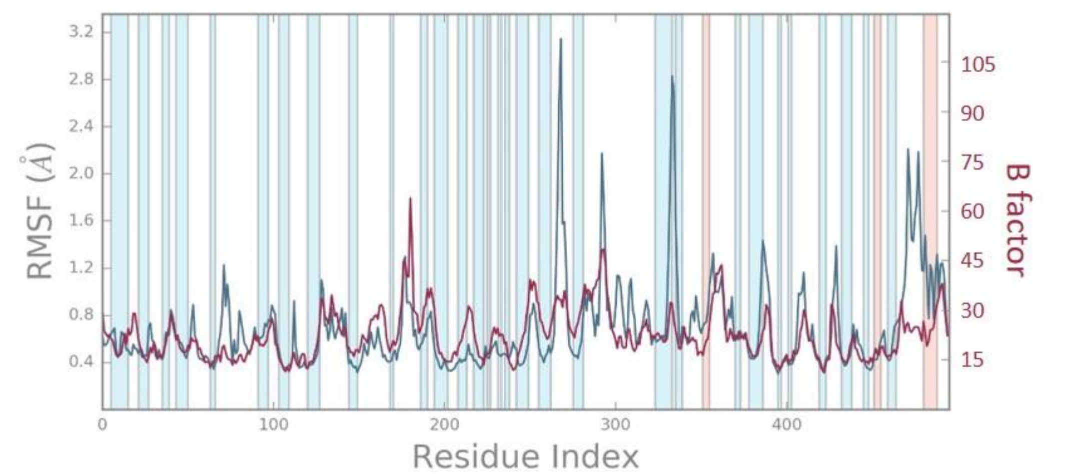
</td>
</tr>
<tr>
<td>CuL/ 3e$^-$ reduced/ O pH 7.0</td>
<td>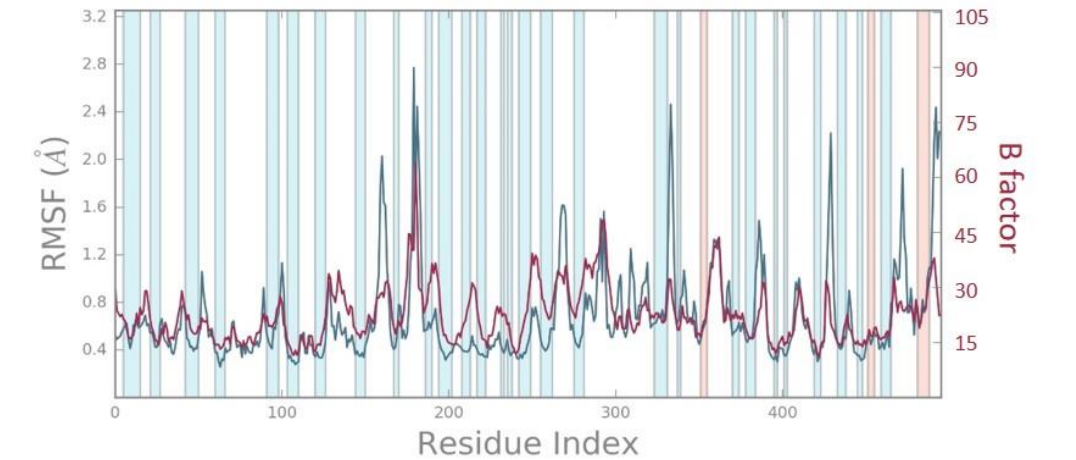
</td>
</tr>
<tr>
<td>CuL/ 3e$^-$ reduced/ pH 4.5</td>
<td>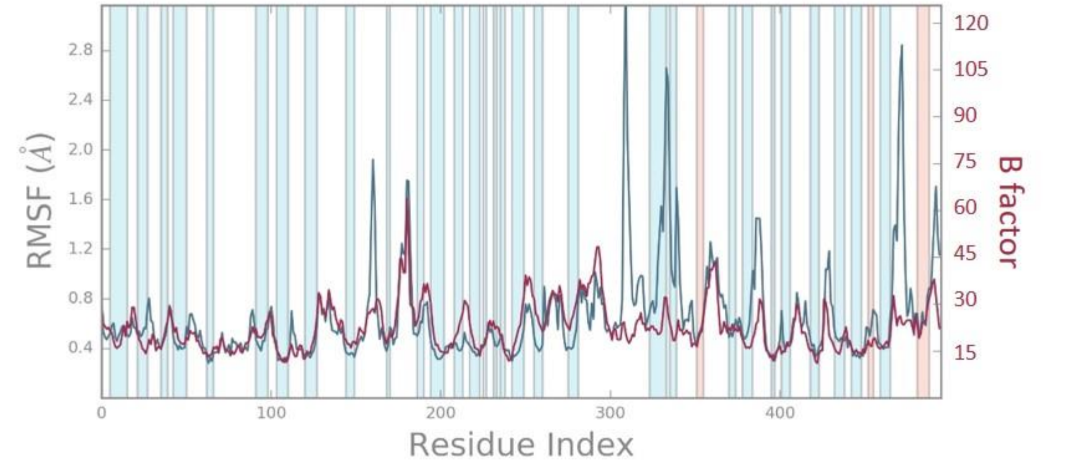
</td>
</tr>
</table>

CuL/ $3e^-$ reduced/ pH 4.5 Ash 206	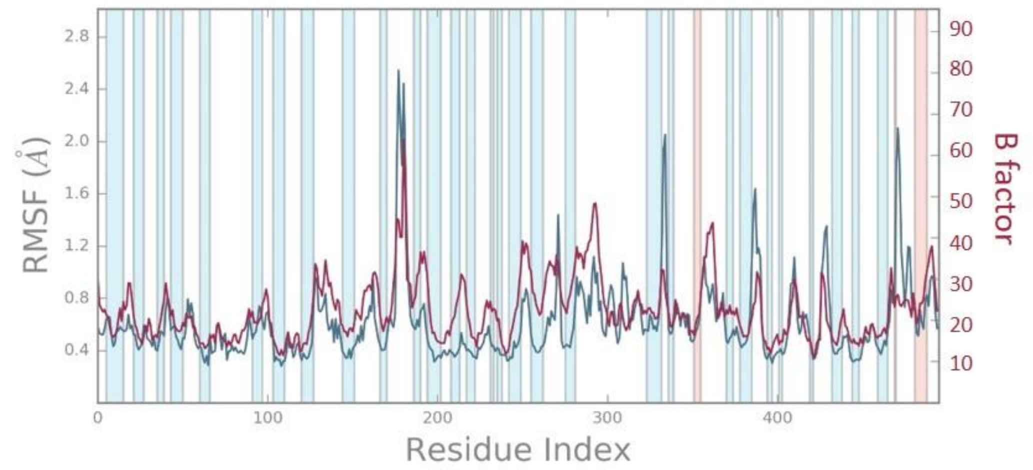

Table S14. Structural comparison (RMSD analysis) of representative structures from MD at neutral and low pH

Protein/ state	Conditions	RMSD (Å)		
		pH 7.0	pH 4.5	pH 4.5 Ash 206
TvL/RO	pH 7.0	-	1.81	2.31
	pH 4.5	-	-	2.20
	pH 4.5 Ash-206	-	-	-
TvL/$3e^-$ reduced	pH 7.0	-	1.99	1.62
	pH 4.5	-	-	1.48
	pH 4.5 Ash-206	-	-	-
CuL/RO	pH 7.0	-	1.84	1.59
	pH 4.5	-	-	1.85
	pH 4.5 Ash-206	-	-	-
CuL/$3e^-$ reduced	pH 7.0	-	1.34	1.69
	pH 4.5	-	-	1.67
	pH 4.5 Ash-206	-	-	-

Supplementary Figures

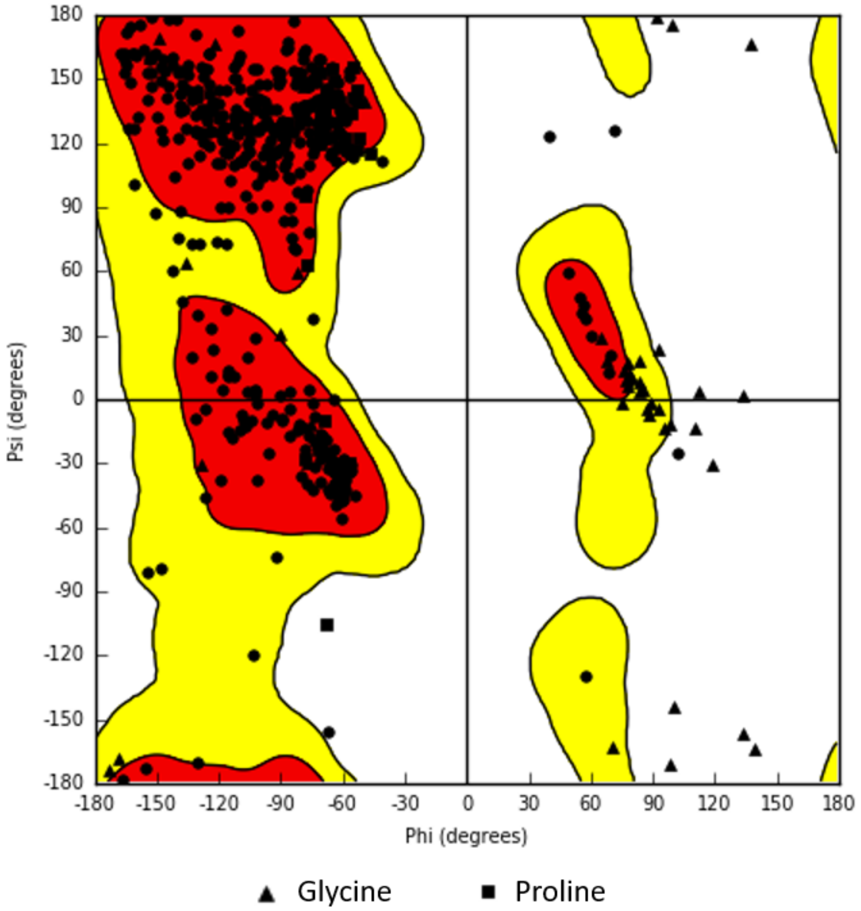

Fig. S1 Ramachandran plot of the CuL structure modeled using Prime homology modeling.

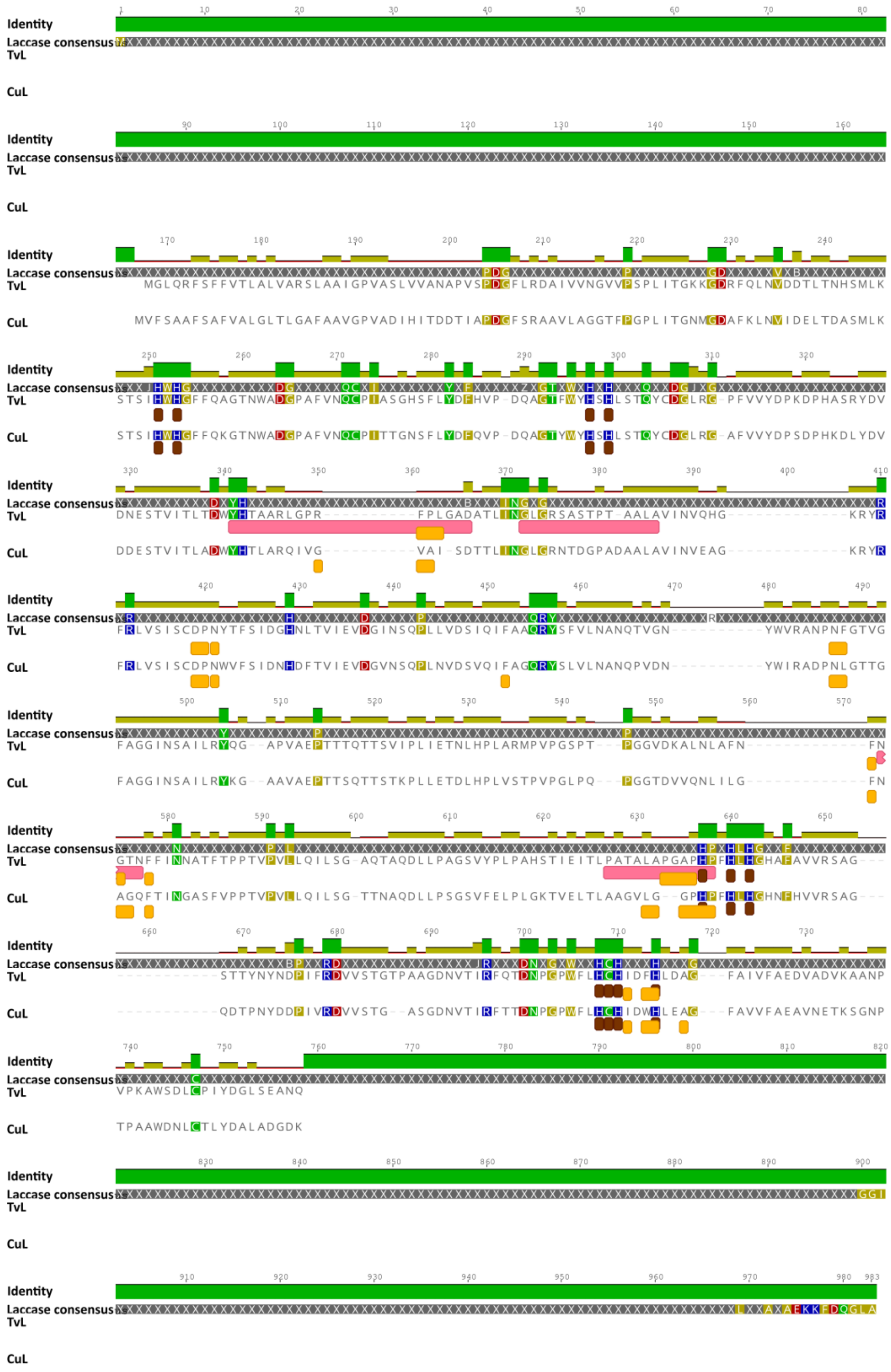

Fig. S2 Sequence alignment of TvL, CuL and the consensus sequence of 924 laccases from basidomycetes. Important features are highlighted below or above the respective sequence: copper binding sites (brown), substrate binding residues (orange), flexible loops close to substrate binding site (red); amino acid residues in agreement with the consensus are highlighted with specific colours based on polarity (positive charge: blue; negative charge: red; polar uncharged: green; apolar uncharged: olive green).

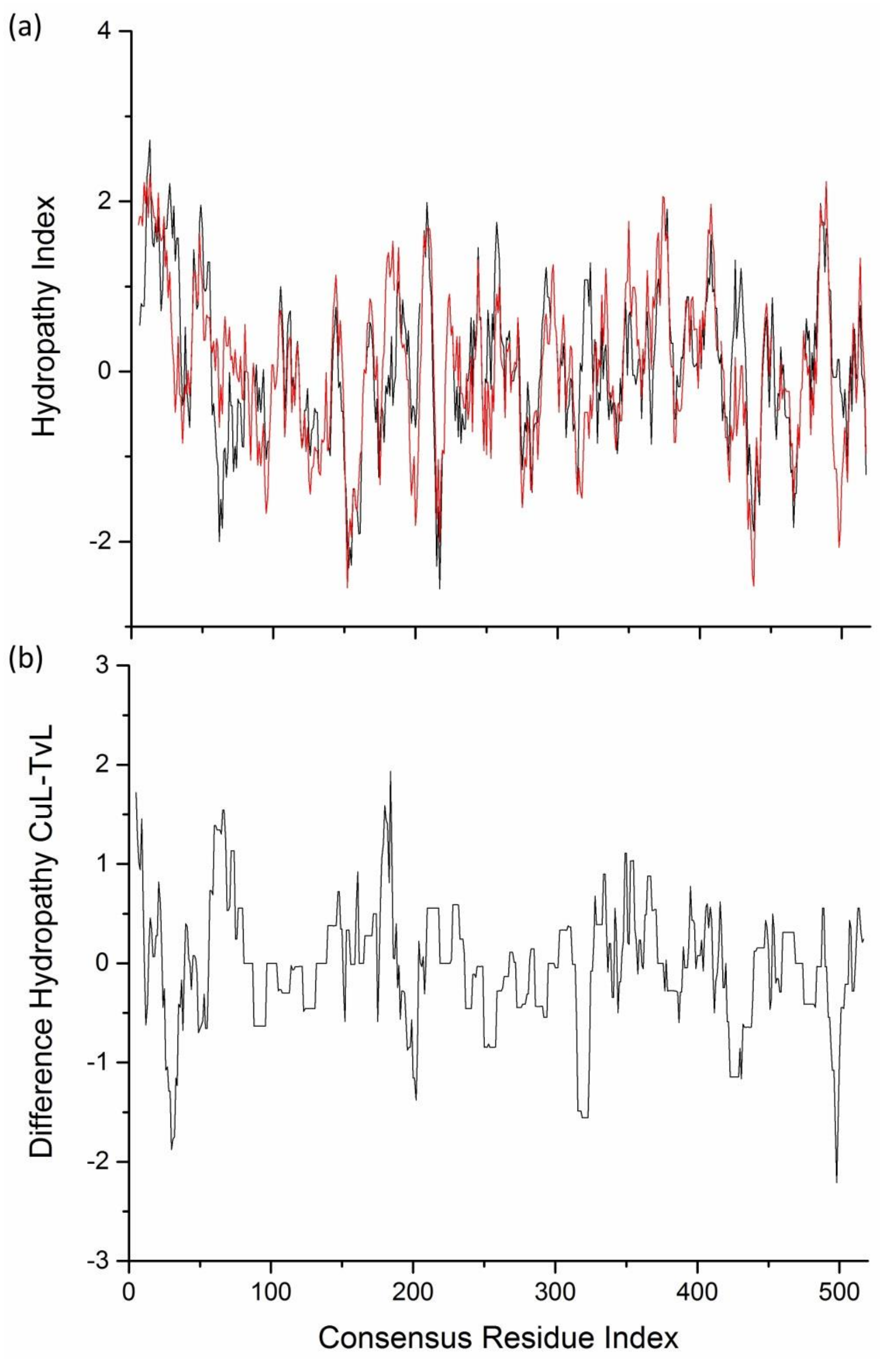

Fig. S3 (a) Hydrophobicity of TvL (black) and CuL (red); (b) Difference in hydrophobicity calculated by CuL-TvL.

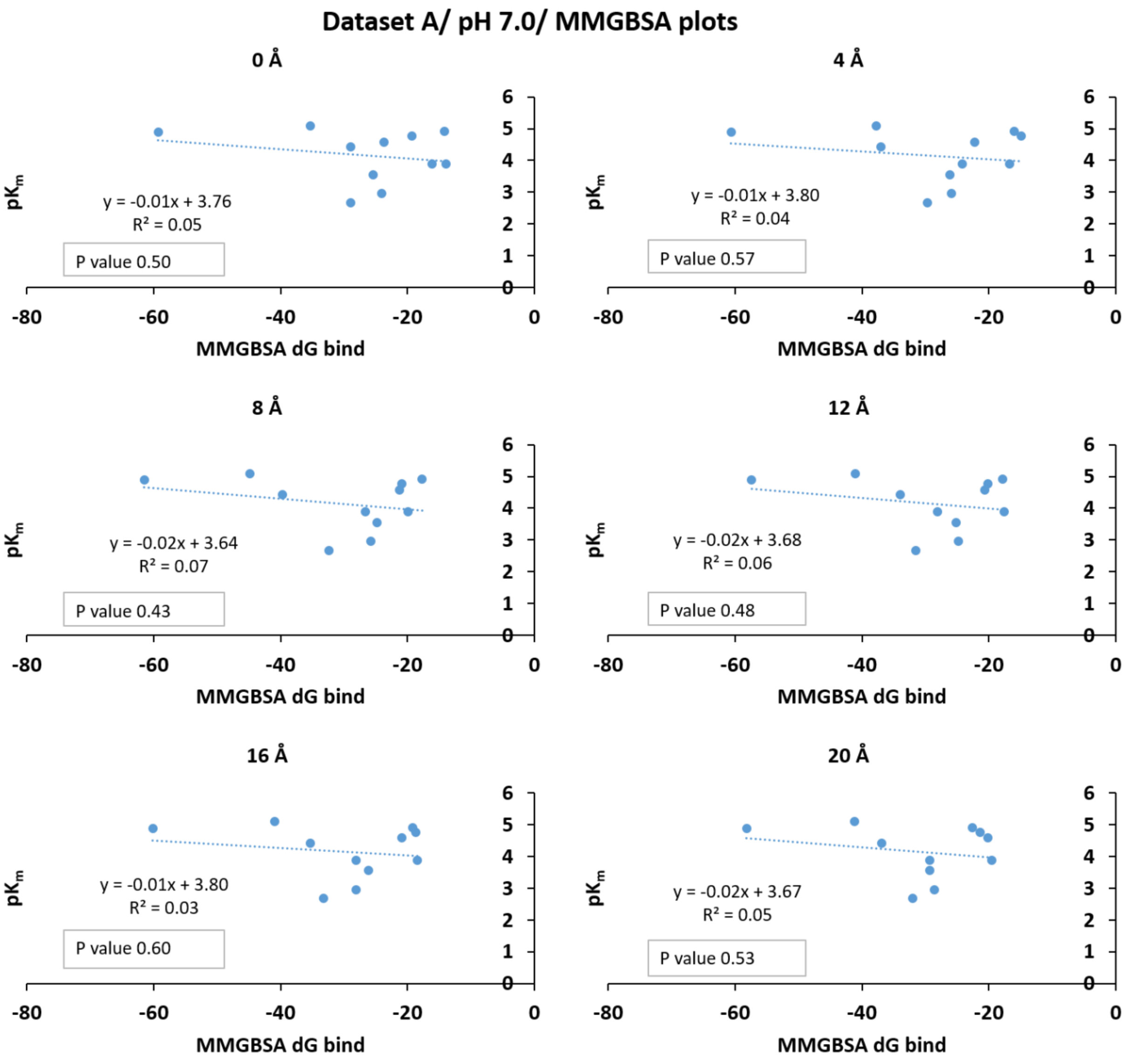

Fig. S4 Correlation of dataset A MMGBSA dG bind energies with experimental pK_m values at protein flexibility distances from 0 Å to 20 Å for the protein and ligands structures prepared at pH 7.0.

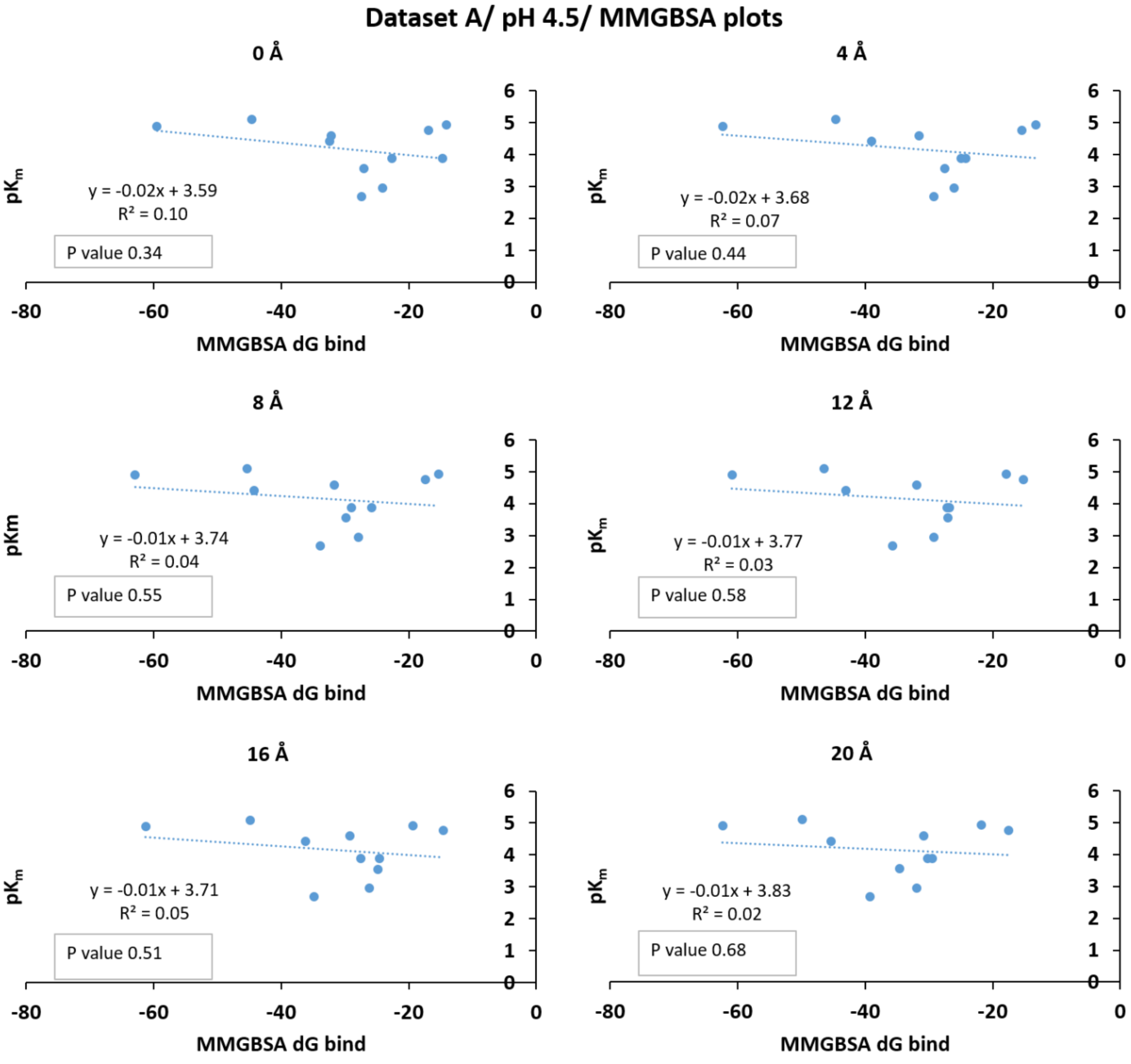

Fig. S5 Correlation of dataset A MMGBSA dG bind energies with experimental pK_m values at protein flexibility distances from 0 Å to 20 Å for the protein and ligands structures prepared at pH 4.5.

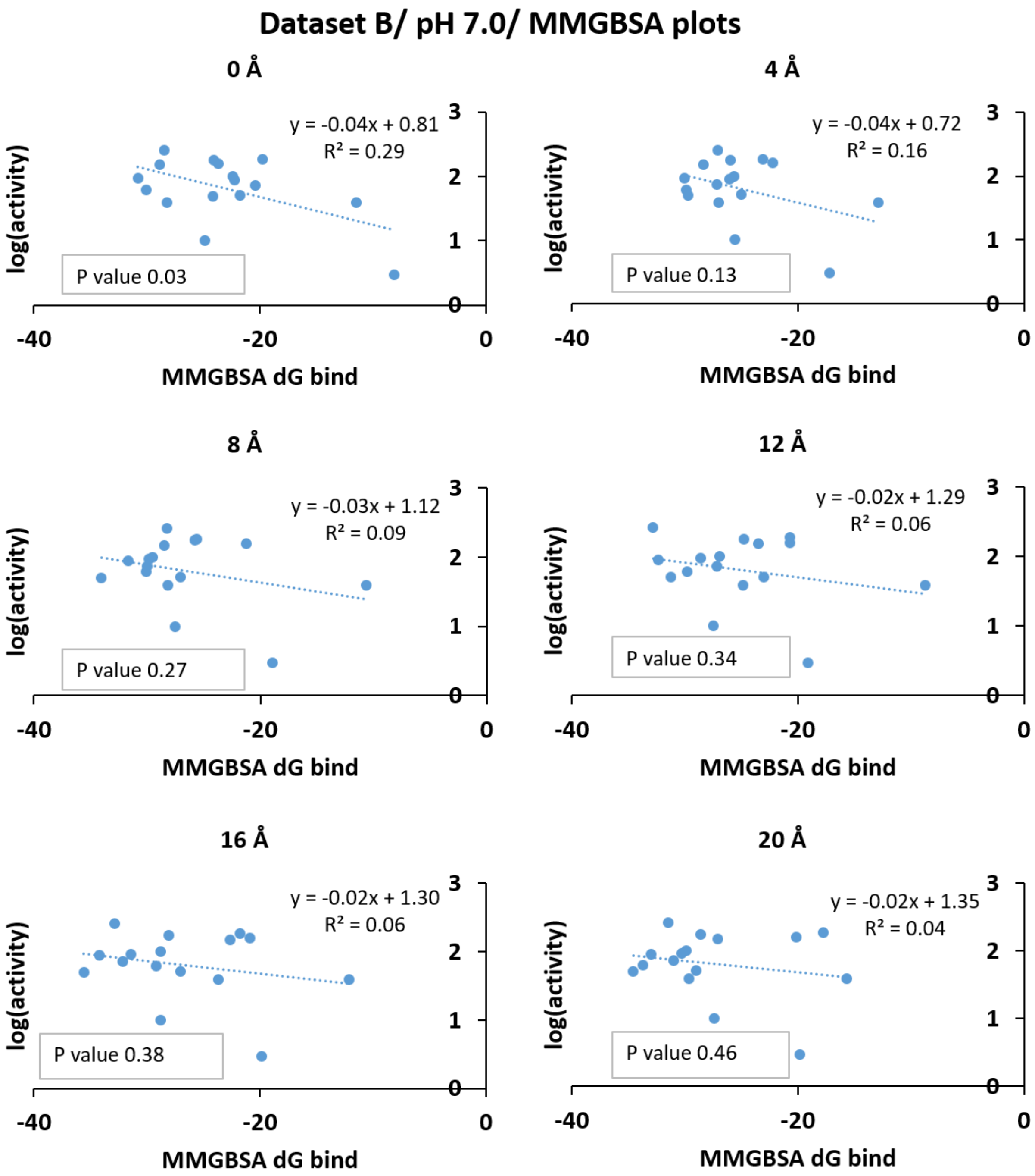

Fig. S6 Correlation of dataset B MMGBSA dG bind energies with log (relative activity) values at protein flexibility distances from 0 Å to 20 Å for the protein and ligands structures prepared at pH 7.0.

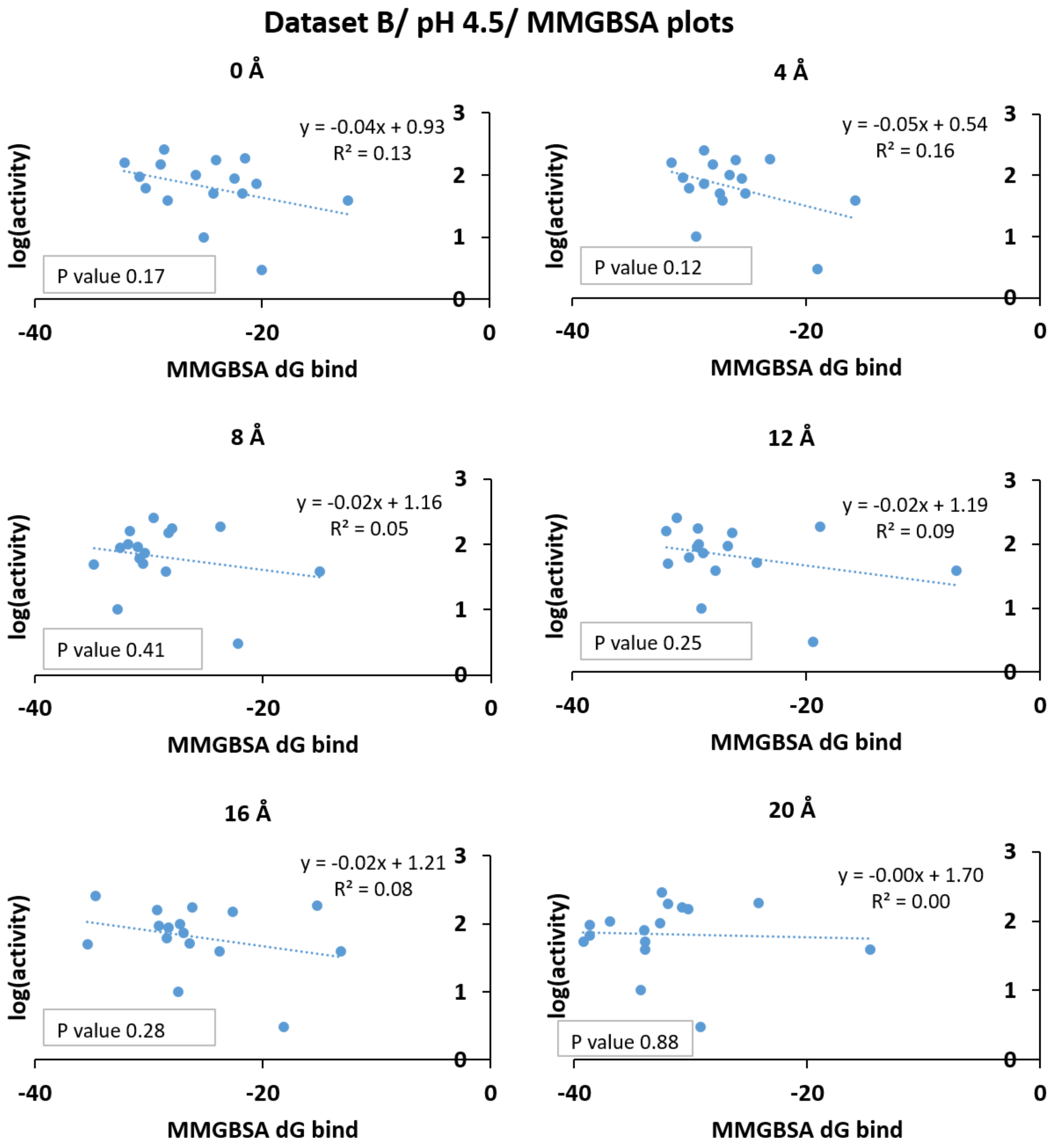

Fig. S7 Correlation of dataset B MMGBSA dG bind energies with log (relative activity) values at protein flexibility distances from 0 Å to 20 Å for the protein and ligands structures prepared at pH 4.5.

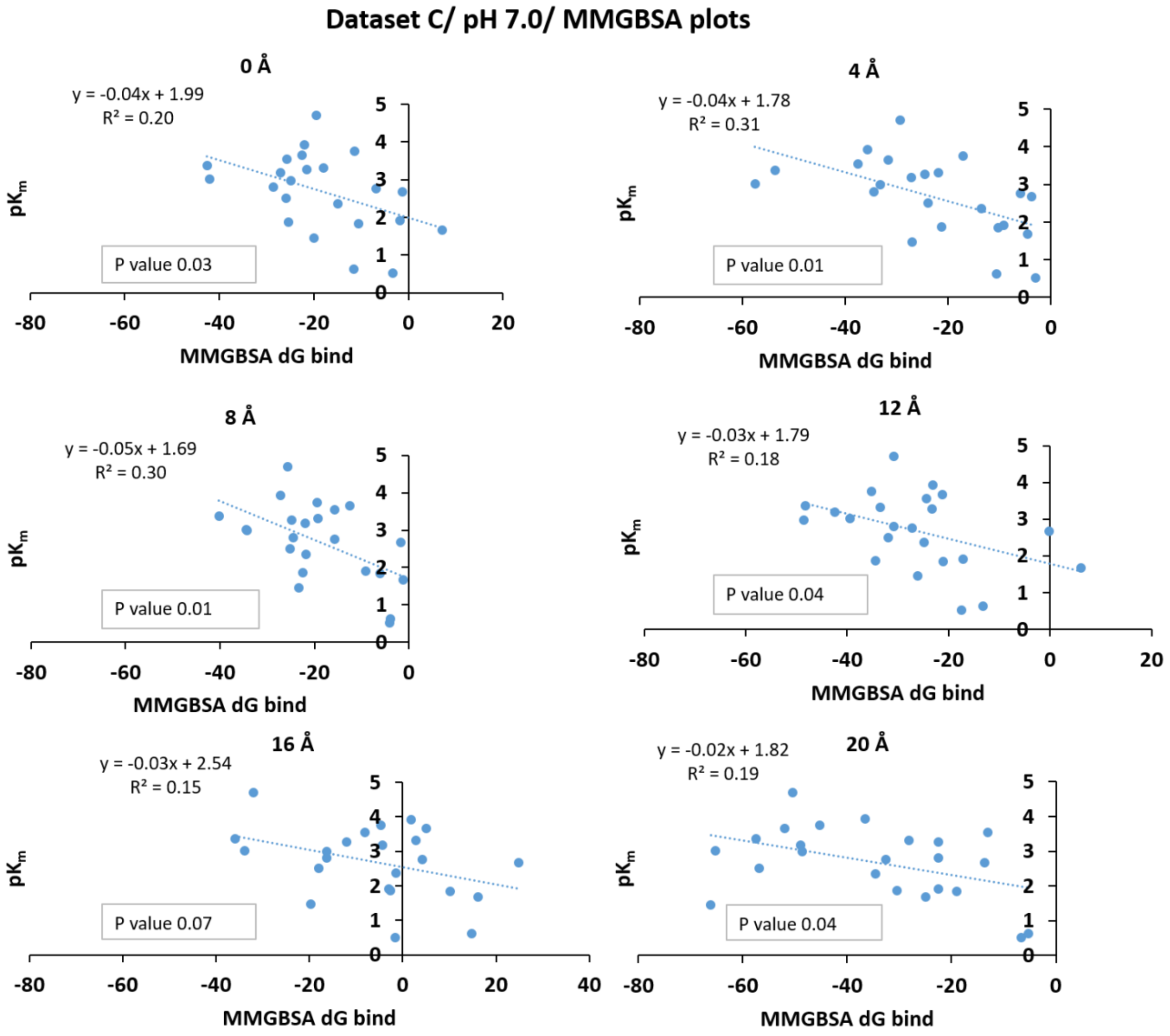

Fig. S8 Correlation of MMGBSA binding energies with pK_m values for dataset C at protein flexibility distances from 0 Å to 20 Å for the protein and ligands structures prepared at pH 7.0.

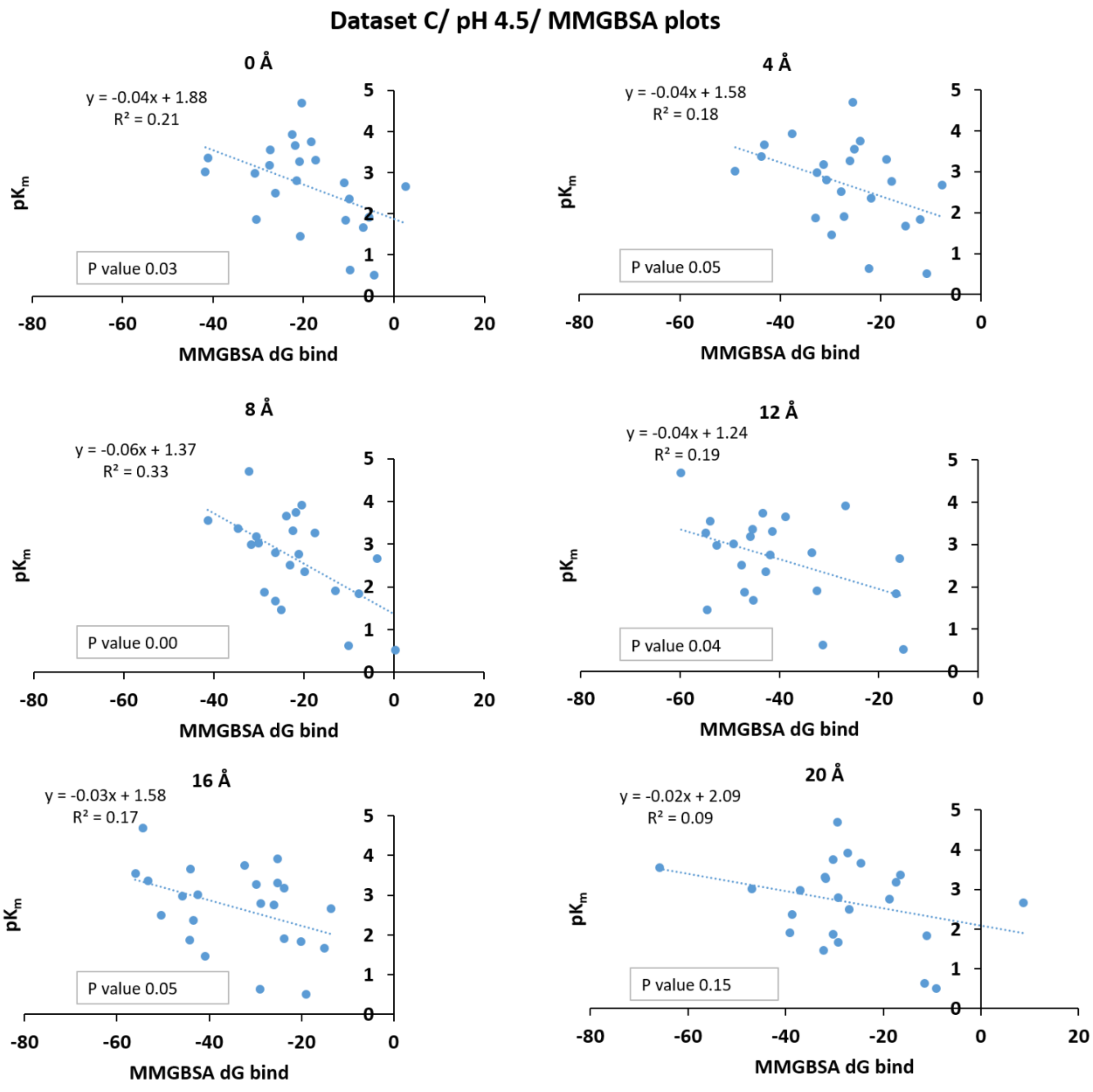

Fig. S9 Correlation of dataset C MMGBSA dG bind energies with pK_m values at protein flexibility distances from 0 Å to 20 Å for the protein and ligands structures prepared at pH 4.5.

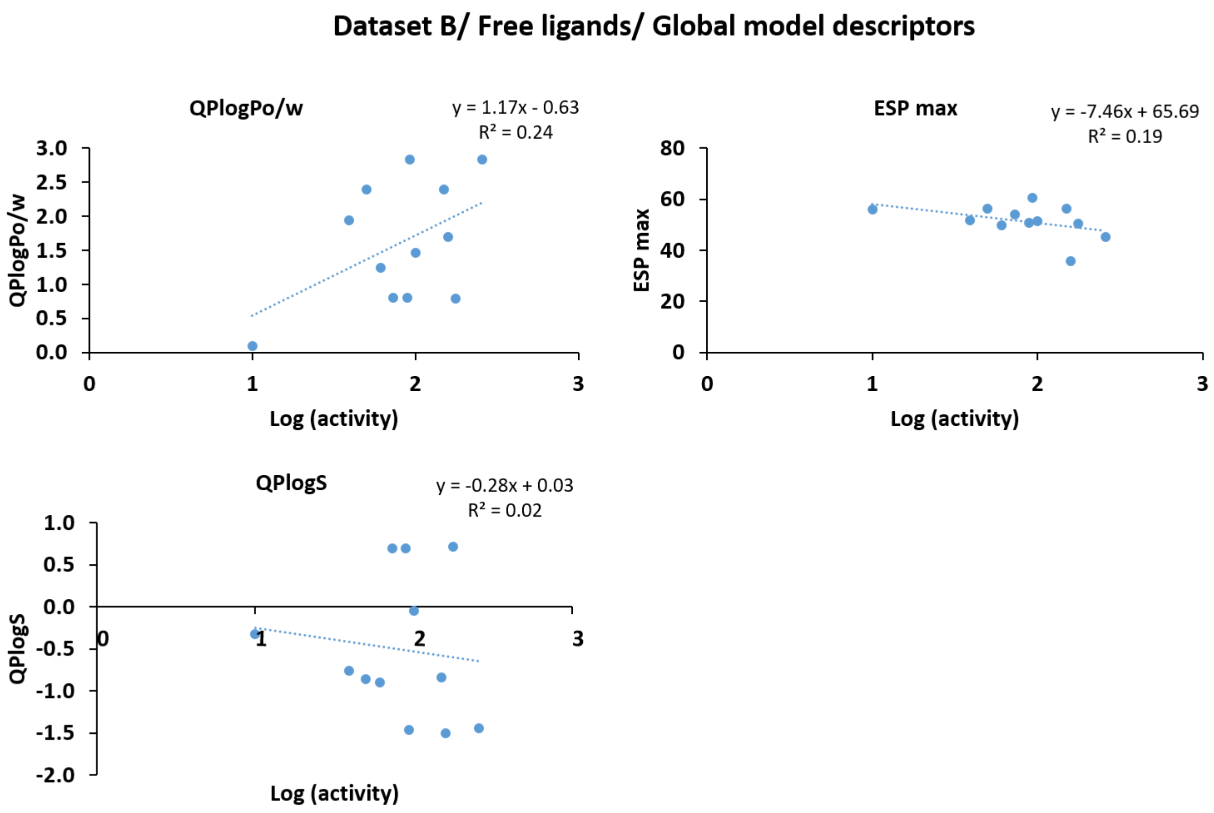

Fig. S10 Dataset B/ Free ligands/ Global model descriptor's correlation with log (relative activity).

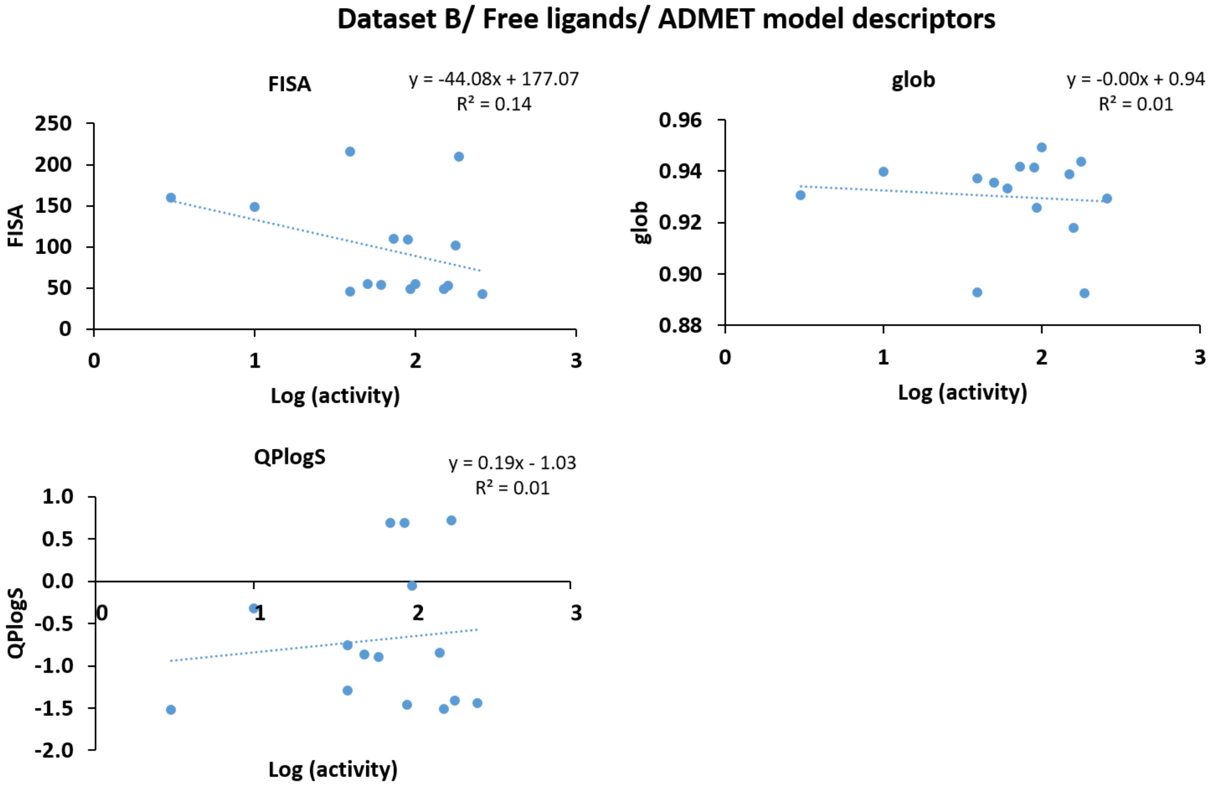

Fig. S11 Dataset B/ Free ligands/ ADMET model descriptor's correlation with log (relative activity).

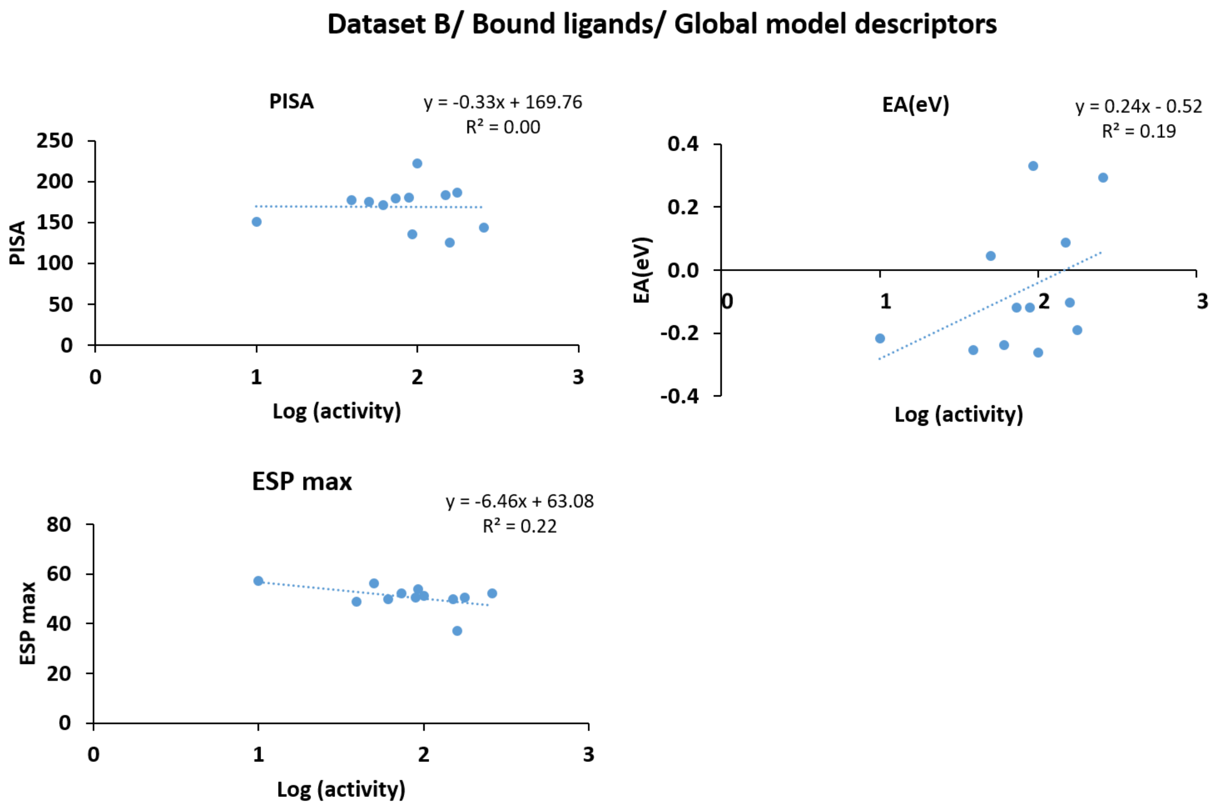

Fig. S12 Dataset B/ Bound ligands/ Global model descriptor's correlation with log (relative activity).

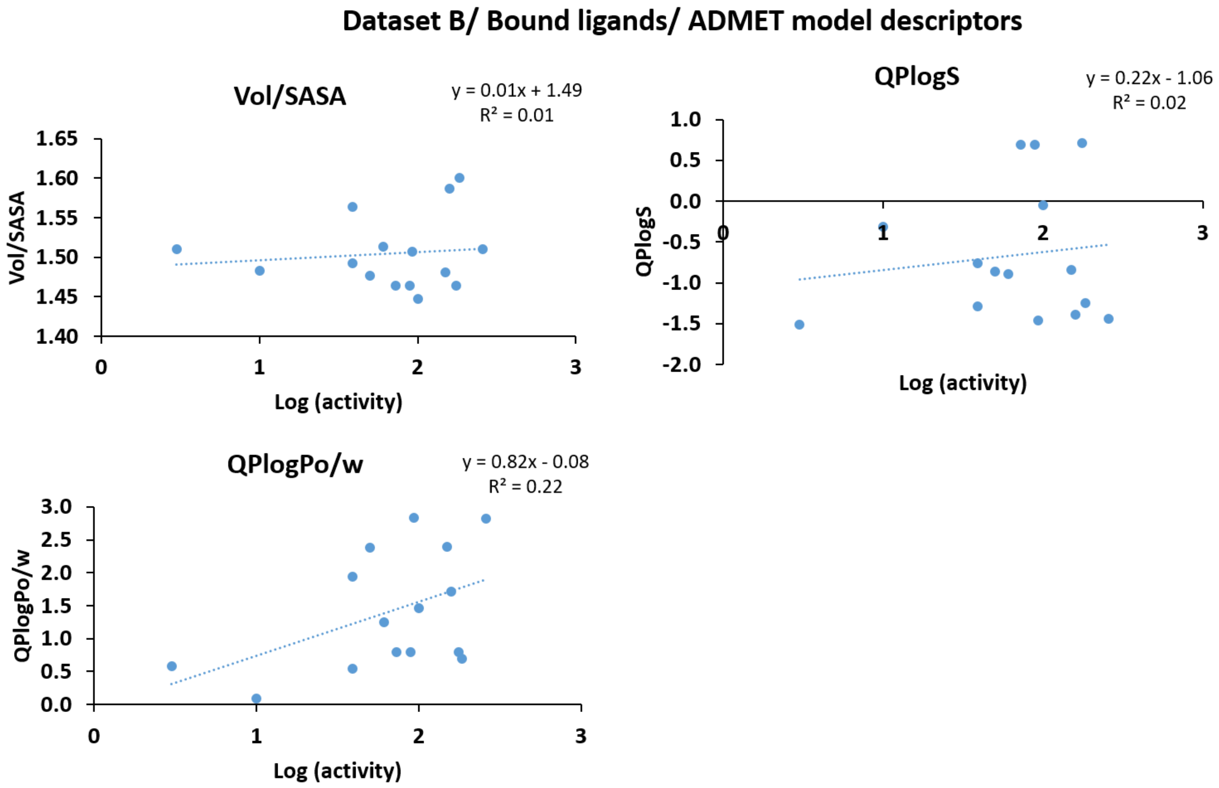

Fig. S13 Dataset B/ Bound ligands/ ADMET model descriptor's correlation with log (relative activity).

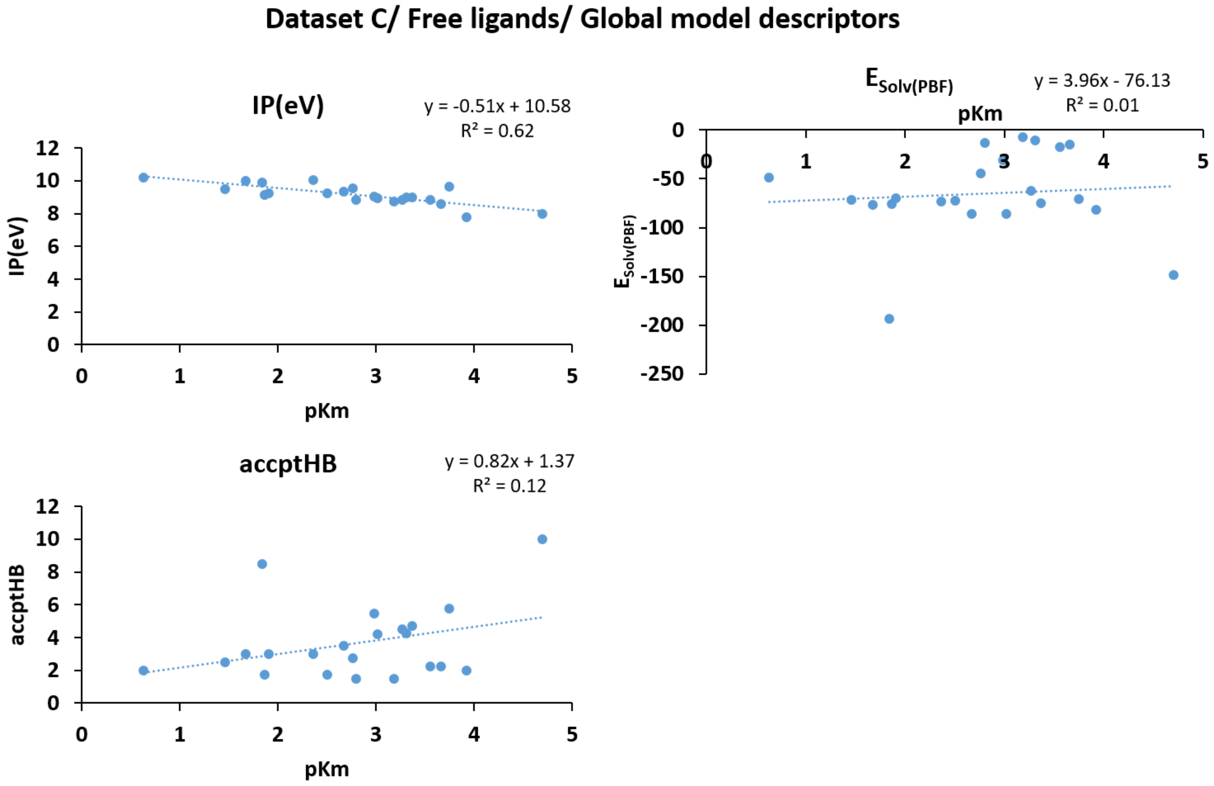

Fig. S14 Dataset C/ Free ligands/ Global model descriptor correlation with pK_m.

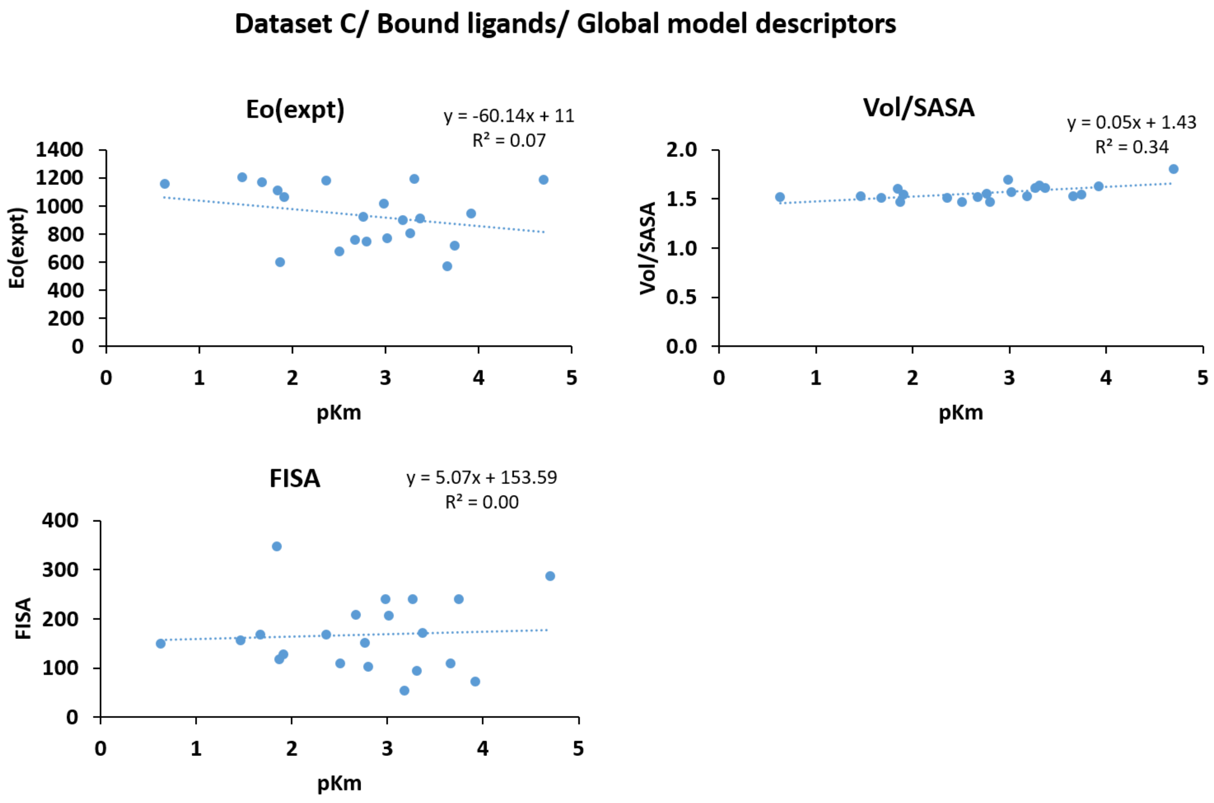

Fig. S15 Dataset C/ Bound ligands/ Global model descriptor correlation with pK_m.

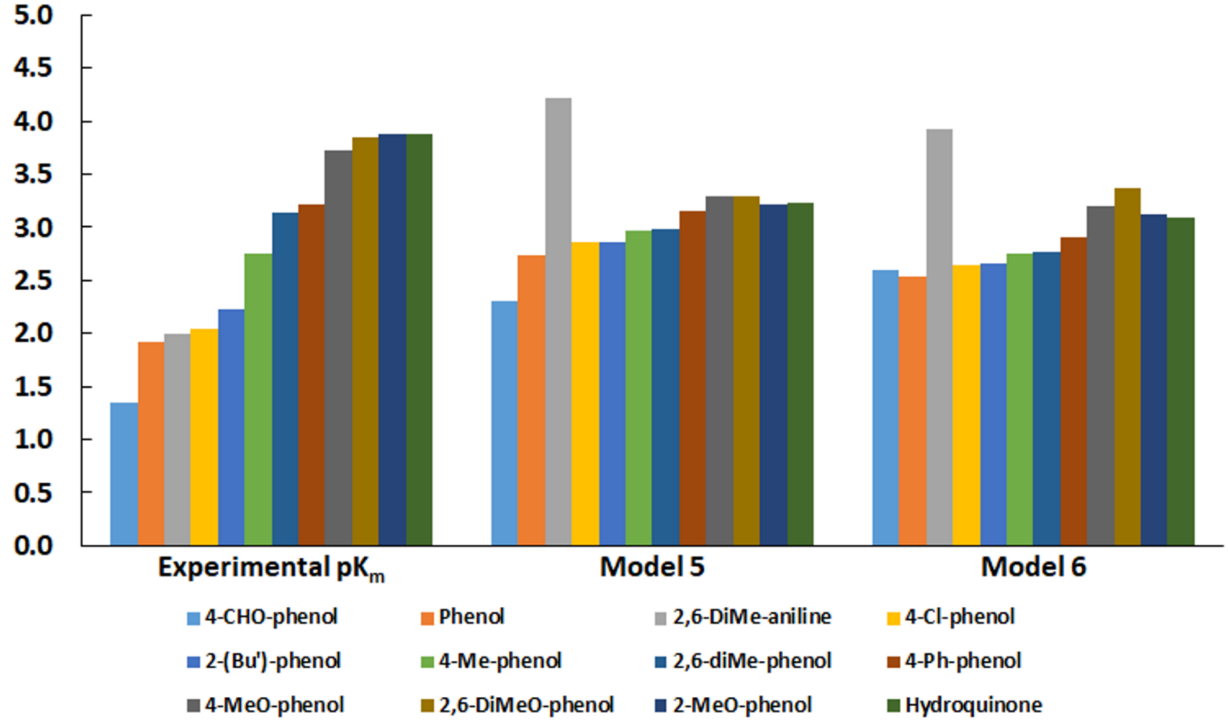

Fig. S16. Plot of the experimental and the predicted pK_m of the twelve laccase substrates using models 5 and 6 of the main article. The substrate 2,6-dimethylaniline is an outlier in the dataset (all others are phenols) and showed comparatively high predicted pK_m.

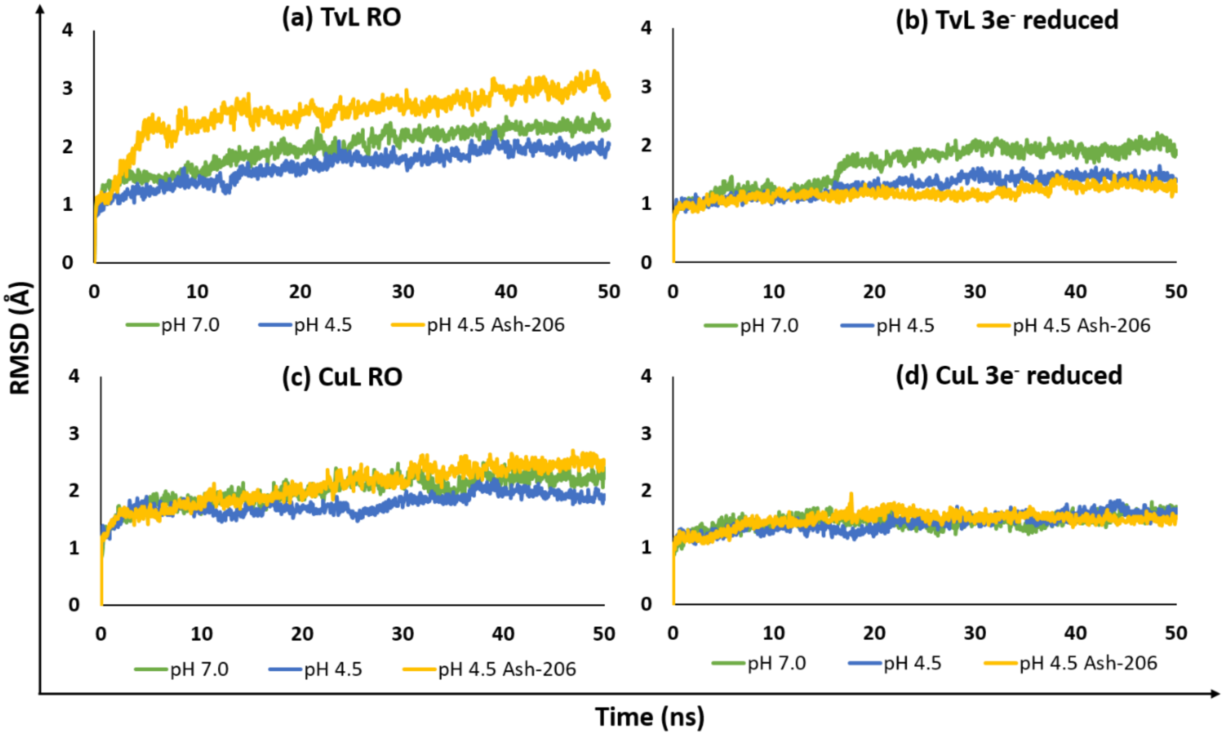

Fig. S17 RMSD plots of the twelve MD complexes for 50 ns. (a) Plot for the RO state of TvL complexes. (b) Plot for the 3e$^-$ reduced state of TvL. (c) CuL RO complexes. (d) CuL 3e$^-$ reduced complexes.
